# Pan-Cancer Analysis of ART1 and its Potential Value in Gastric Cancer

**DOI:** 10.7150/jca.96033

**Published:** 2024-05-13

**Authors:** Zhiping Wu, Siyuan Song, Jiayu Zhou, Qiling Zhang, Jiangyi Yu

**Affiliations:** 1 Department of Traditional chinese medicine, Jinjiang Municipal Hospital (Shanghai Sixth People's Hospital Fujian Campus), Jinjiang, China.; 2 Department of Endocrinology, Jiangsu Province Hospital of Chinese Medicine, Affiliated Hospital of Nanjing University of Chinese Medicine, Nanjing, China.; 3 Department of Oncology, Wuxi Hospital of Chinese Medicine, Affiliated Hospital of Nanjing University of Chinese Medicine, Wuxi, China.

**Keywords:** Mono-ADP-ribosyltransferases-1, Biomarker, Prognosis, Bayesian co-localization analysis, Experimental validation

## Abstract

**Objective:** To comprehensively explore the impact of Mono-ADP-ribosyltransferases-1 expression on both prognosis and the intricate landscape of the tumor immune microenvironment across diverse cancer types, our study seeks to delve into the multifaceted interplay between Mono-ADP-ribosyltransferases-1 expression levels and their implications for clinical outcomes and the dynamic milieu of immune responses within tumors.

**Methods:** Genomic, transcriptomic, and clinical datasets spanning diverse cancer types were meticulously curated from The Cancer Genome Atlas and Genotypic Tissue Expression repositories. Initially, our inquiry focused on discerning the prognostic significance and immunological implications of Mono-ADP-ribosyltransferases-1 expression across this heterogeneous spectrum of malignancies. Subsequently, we scrutinized the relationships between Mono-ADP-ribosyltransferases-1 expression levels and a spectrum of factors including RNA modification genes, genetic mutations, and the emergent concept of tumor stemness. Employing functional enrichment analyses, we endeavored to unravel the underlying mechanistic pathways modulated by Mono-ADP-ribosyltransferases-1. Leveraging Bayesian co-localization analysis, we sought to discern the spatial convergence of Mono-ADP-ribosyltransferases-1 expression particularly within the context of digestive tract tumors. Lastly, to corroborate our findings, we conducted *in vitro* experiments, specifically focusing on Gastric Cancer, thus corroborating the putative oncogenic role attributed to Mono-ADP-ribosyltransferases-1 in this malignancy.

**Results:** Across diverse tumor types, Mono-ADP-ribosyltransferases-1 expression exhibits distinctive patterns compared to normal and adjacent tissues, thereby intertwining with the prognostic outcomes of numerous cancer patients. Noteworthy findings from our immune role identification underscore the pivotal involvement of Mono-ADP-ribosyltransferases-1 in the landscape of tumor immunotherapy. Furthermore, Kyoto Encyclopedia of Genes and Genomes analysis elucidates the enrichment of Mono-ADP-ribosyltransferases-1-associated genes predominantly within the NF-kB, Foxo, and PI3K-Akt signaling cascades, shedding light on potential mechanistic pathways underlying its influence. Bayesian co-localization analysis unveils a compelling genetic correlation between Mono-ADP-ribosyltransferases-1 and digestive tract tumors, accentuating its relevance within this specific oncological domain. Importantly, experimental validation attests to the therapeutic promise of targeting Mono-ADP-ribosyltransferases-1 in the treatment paradigm of gastric cancer, thereby underscoring its potential as a viable therapeutic target deserving of further exploration and clinical translation.

**Conclusion:** This comprehensive pan-cancer analysis unveils crucial insights into the intricate role played by Mono-ADP-ribosyltransferases-1 in the tumorigenesis of diverse malignancies, thereby establishing a robust theoretical framework for subsequent in-depth investigations. Leveraging these insights, targeting Mono-ADP-ribosyltransferases-1-related signaling pathways within the dynamic tumor microenvironment emerges as a promising avenue for novel therapeutic interventions in the realm of tumor immunotherapy. By delineating the interplay between Mono-ADP-ribosyltransferases-1 expression and tumorigenic processes across various cancer types, this study paves the way for innovative therapeutic strategies aimed at disrupting oncogenic signaling cascades and bolstering immune-mediated antitumor responses.

## 1. Introduction

Cancer represents a formidable global health challenge, with its incidence steadily rising worldwide. Gastric cancer (GC), ranking as the fifth most prevalent malignancy globally, underscores the urgency of addressing its clinical complexities 1. Owing to the insidious onset of symptoms and the absence of reliable diagnostic modalities, GC frequently advances undetected, resulting in dismal prognoses, where the 5-year survival rate languishes between 20% to 30% 2. Presently, clinical management strategies for GC encompass a multifaceted approach, including surgical resection, adjuvant and neoadjuvant chemotherapy, radiotherapy, immunotherapy, and anti-angiogenesis therapy. Notably, the advent of immune checkpoint inhibitors (ICI) has heralded a transformative era, markedly enhancing outcomes across various malignancies. However, the inherent heterogeneity of GC engenders disparate responses to immunotherapeutic interventions among patients sharing similar disease stages 3, thereby posing challenges such as immune-related adverse events (irAE) and therapeutic resistance 4. Hence, the quest for robust biomarkers capable of accurately prognosticating outcomes and predicting responses to immunotherapy in GC assumes paramount significance. Leveraging the paradigm of pan-cancer analysis, which entails the systematic exploration of genes across diverse malignancies utilizing invaluable resources such as The Cancer Genome Atlas (TCGA), emerges as a potent avenue for identifying pivotal prognostic and immunotherapeutic determinants 5.

Mono-ADP-ribosyltransferases-1 (ART1), identified as a pivotal arginine-specific mono-ADP-ribosylase, holds a central position in cancer biology 6. Notably, the expression dynamics of ART1 exert profound regulatory effects encompassing crucial hallmarks of cancer progression, including proliferation, apoptosis, adhesion, migration, metastasis, and angiogenesis, particularly within the milieu of mouse colorectal cancer cells 7. Intriguingly, an upregulation of ART1 expression is discerned in human colorectal cancer tissues in comparison to control colorectal mucosal tissue, accentuating its putative oncogenic role in this context 8. Mechanistically, knockdown of ART1 orchestrates a cascade of molecular events culminating in the inhibition of the PI3K/Akt/NF-kB signaling axis, concomitant downregulation of BCL-xl and BCL-2 proteins, and upregulation of Bax protein expression, thereby fostering apoptosis in mouse CT26 cells 9. Further delineating its multifaceted role, ART1 catalyzes mono-ADP-ribosylation modification of arginine residues at positions 470 and 492 of GRP78 in cervical cancer cells, thereby modulating the endoplasmic reticulum stress response mediated by GRP78 10. These seminal observations underscore the pivotal involvement of ART1 in the initiation and progression of tumorigenesis. However, despite these illuminating insights, the expression patterns, clinical significance, and biological functions of ART1 in the context of GC remain largely unexplored, thus warranting comprehensive investigation to unravel its enigmatic role within this malignancy.

**Figure 1:**
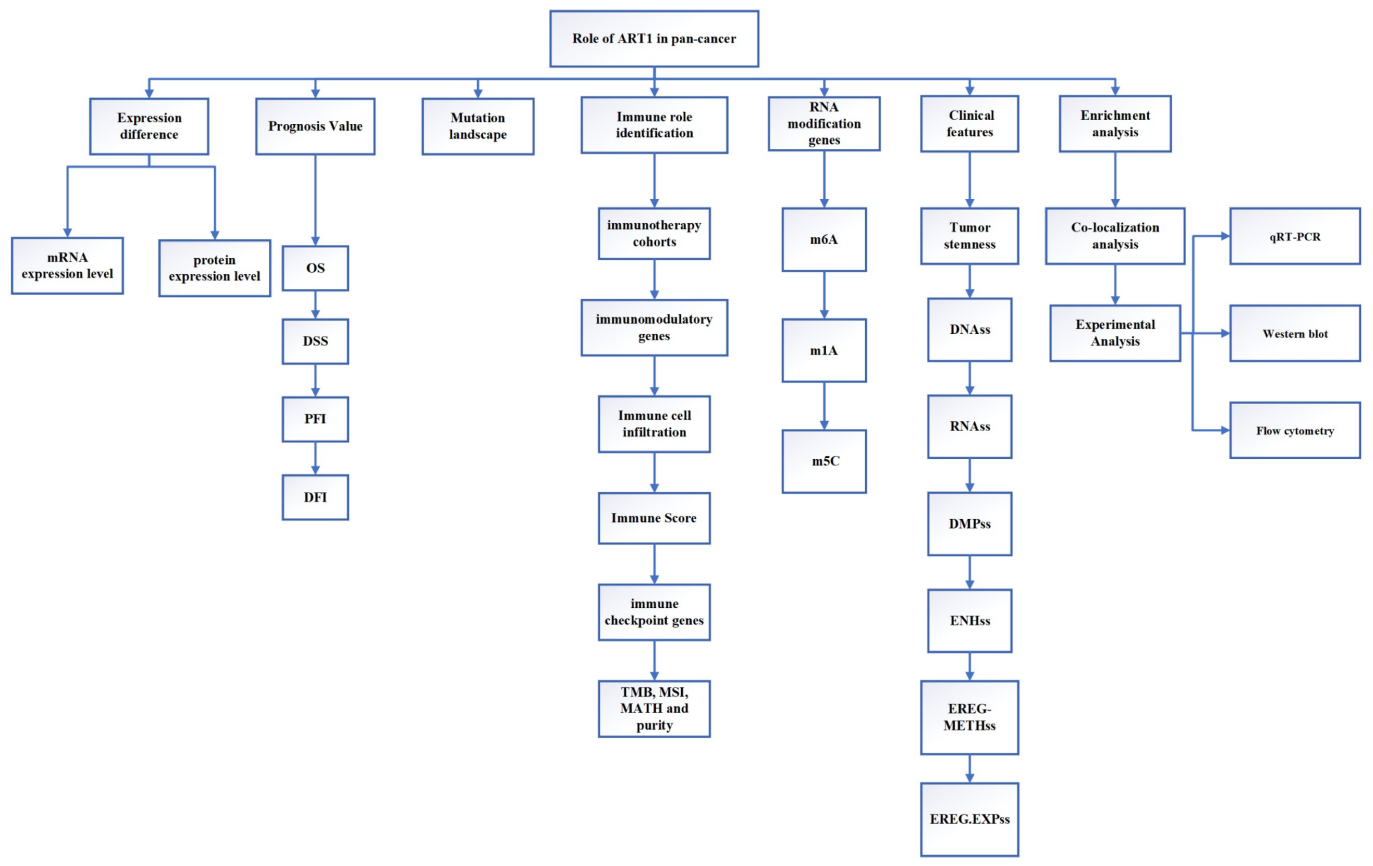
The flow chart of this study.

This study employed extensive transcriptional datasets sourced from the TCGA database to meticulously investigate the intricate interplay between ART1 expression and a spectrum of factors across diverse cancers. These factors encompassed crucial determinants such as prognosis, immune-related genes, immune score, tumor heterogeneity metrics (including TMB, MATH, MSI, and purity), RNA modification genes, clinical characteristics, and tumor stemness. Through this comprehensive analysis, we endeavored to unveil the nuanced role played by ART1 in driving cancer progression and shaping the TIME, thereby furnishing invaluable insights for the prospective exploration of ART1 as a promising target for tumor immunotherapy and prognostic biomarker development. The schematic depiction of our study's methodology and analytical framework is encapsulated in Figure 1, delineating the sequential flow of our investigative approach.

## 2. Materials and Methods

### 2.1 Bioinformatics analysis

#### 2.1.1 Data download and processing

The pan-cancer RNA sequencing data, survival data, and clinicopathological attributes spanning diverse cancer types were meticulously gathered from the online UCSC database (https://xena.ucsc.edu/), a comprehensive repository derived from the esteemed TCGA database (https://www.cancer.gov/ccg/research/genome-sequencing/tcga) 11. Each expression value underwent a Log2(x+0.001) transformation to ensure robustness in downstream analyses, with cancer types boasting fewer than three samples being excluded from the study cohort. Consequently, a total of 41 cancer types were scrutinized, including but not limited to ACC, BLCA, BRCA, CESC, CHOL, COAD, COADREAD, DLBC, ESCA, GBM, GBMLGG, HNSC, KICH, KIPAN, KIRC, KIRP, LAML, LGG, LIHC, LUAD, LUSC, MESO, OV, PAAD, PCPG, PRAD, READ, SARC, SKCM, STAD, STES, TGCT, THCA, THYM, UCEC, UCS, UVM, OS, ALL, NB, and WT. Given the study's focus on GC, specific clinical parameters from the TCGA-STAD cohort are delineated in Supplementary Table 1.

The differential mRNA expression patterns of ART1 between normal and tumor samples across each cancer type were meticulously assessed utilizing the unpaired Wilcoxon test method within the R software environment. Additionally, to comprehensively elucidate the differential expression profile of ART1 at the protein level, the Human Protein Atlas (HPA) database (https://www.proteinatlas.org/) was harnessed. Immunohistochemical (IHC) images capturing the differential expression of ART1 across a spectrum of tumor tissues and their corresponding normal counterparts were meticulously procured. The encompassed cancer types in this analysis included UCEC, THCA, LUAD, LUAD, STAD, BRCA, KIPAN, PAAD, CESC, and PRAD, thus affording a comprehensive portrayal of ART1 expression dynamics within diverse malignancies.

#### 2.1.2 ART1 prognostic and immune role identification

The dataset, initially sourced from TCGA-provided samples, was further augmented by supplementary TARGET follow-up data retrieved from UCSC, particularly for samples with a follow-up duration of fewer than 30 days 12. Leveraging the versatile "Forest Plot" R package, we meticulously scrutinized the association between ART1 expression and pan-cancer prognostic outcomes. Univariate Cox regression analysis was subsequently employed to ascertain the prognostic significance of ART1 in predicting Overall Survival (OS), Disease-Specific Survival (DSS), Disease-Free Interval (DFI), and Progression-Free Interval (PFI) across a diverse array of malignancies. Survival curves were thoughtfully generated utilizing the "survival" and "Survivminer" R packages, offering a comprehensive visualization of ART1's prognostic relevance across diverse cancer types.

In delineating ART1's potential utility as a pan-cancer immunotherapeutic target, we scrutinized its responsiveness within immunotherapy cohorts utilizing the TISMO database (http://tismo.cistrome.org). Immunological characteristics were comprehensively assessed, encompassing the expression profiles of immune regulatory genes, immune checkpoint genes, and the extent of immune cell infiltration. Immune modulatory genes, including chemokines, receptors, MHC molecules, immune inhibitors, and immune stimulators, were thoroughly interrogated 13, 14. Furthermore, the assessment of immune checkpoint genes, spanning both inhibitory and stimulatory types, contributed to a nuanced understanding of ART1's immunotherapeutic potential 15. To further delineate the complex interplay between ART1 expression and the TME, key biomarkers including Tumor Mutational Burden (TMB) and Microsatellite Instability (MSI) were meticulously assessed. Utilizing SangerBox (http://sangerbox.com/Tool), an intuitive online platform for TCGA data analysis, we explored the relationships between ART1 expression and crucial TME biomarkers including TMB, MSI, Mutant-allele tumor heterogeneity (MATH), and tumor purity 14, 16-18. TMB, a quantifiable indicator of immune response, was closely scrutinized for its association with the efficacy of PD-1/PD-L1 inhibitors 19, while MSI, stemming from deficient mismatch repair (MMR), held prognostic significance 20. Moreover, MATH, indicative of tumor heterogeneity, and tumor purity, reflecting tumor cell content, were assessed for their clinical implications 21, 22. Correlation analyses were meticulously conducted utilizing Spearman's rank test, offering insights into the intricate relationships between ART1 expression and the dynamic landscape of the TME biomarkers mentioned above 23.

#### 2.1.3 Mutation landscape

Mutation data and gene expression data from TCGA samples, meticulously processed utilizing MuTect2 software 24, were acquired from the Genomic Data Commons (GDC) platform (https://portal.gdc.cancer.gov/) for a comprehensive analysis of ART1 mutations. To unravel the genetic alteration landscape of ART1, we leveraged the versatile "Cancer Types Summary" module available on the cBioPortal website (https://www.cbioportal.org/). This comprehensive analysis facilitated the retrieval of essential metrics including alteration rates, mutation types, and copy number alterations (CNA) pertaining to ART1 across 33 distinct cancer types. Through this systematic interrogation, we sought to garner insights into the prevalence and diversity of genetic alterations affecting ART1 across a broad spectrum of malignancies, thereby offering a comprehensive perspective on its potential role in cancer pathogenesis.

#### 2.1.4 Correlation Analysis between ART1 Expression and RNA modification genes

Regulatory mechanisms governing m6A RNA methylation have garnered significant attention in the context of human diseases, particularly cancer 25. Similarly, m5C modification has emerged as a pivotal regulator exerting influence over various facets of gene expression, including RNA output, ribosomal assembly, translation, and RNA stability 26. Perturbations in m1A regulatory factors have been implicated in potentially impacting protein function within tumor biology 27. Leveraging expression data encompassing ART1 and 44 marker genes associated with three prominent types of RNA modifications (m1A, m5C, and m6A), sourced from the UCSC database, we meticulously examined their interplay within each sample. Subsequently, the Pearson correlation analysis was conducted to delineate the relationship between ART1 expression and five distinct immune pathway marker genes. To visually depict these intricate relationships, the R package “RcolorBrewer” was harnessed to generate a correlation heatmap, offering a comprehensive illustration of the associations between ART1 expression and RNA modification genes, thereby providing valuable insights into their potential regulatory roles within the context of immune pathways.

#### 2.1.5 Relationship between ART1 and clinical features, tumor stemness

Utilizing comprehensive clinical information extracted from various tumor samples, we embarked on assessing ART1 expression levels across distinct clinical subtypes prevalent in different tumors. To achieve this, we employed rigorous statistical analyses including t-tests for comparison between two groups and ANOVA for comparison among three or more groups, thus enabling a meticulous evaluation of ART1 expression dynamics within diverse clinical contexts. Furthermore, recognizing the pivotal role played by tumor stem cells in driving tumorigenesis through mechanisms of self-renewal and proliferation 28, we sought to explore their potential interplay with ART1 expression and its implications for cancer immunity. Notably, tumor stem cells have been implicated in the pathogenesis of various malignancies, with emerging evidence suggesting a significant negative correlation between tumor stemness and anti-cancer immunity 29. In line with this, we procured six distinct tumor stemness indices derived from mRNA expression and methylated signatures from seminal studies 30. These indices encompass a comprehensive spectrum of molecular features, including DNA methylation pattern (DNAss), RNA stemness score (RNAss), Epigenetically regulated RNA expression-based (EREG.EXPss), Epigenetically regulated DNA methylation-based (EREG-METHss), Differentially methylated probes-based (DMPss), and Enhancer Elements/DNA methylation-based (ENHss). By integrating these indices into our analysis framework, we aimed to gain deeper insights into the intricate relationship between tumor stemness and ART1 expression, thereby unraveling potential implications for cancer pathogenesis and immune evasion strategies.

#### 2.1.6 Functional Enrichment Analysis

To comprehensively explore the protein-protein interactions (PPIs) of ART1, we employed the STRING database (https://string-db.org/), a valuable resource for deciphering intricate molecular networks. Through this analysis, we aimed to uncover potential interactors of ART1 and elucidate the underlying biological pathways and functions associated with its activity. Subsequently, leveraging Gene Ontology (GO) and Kyoto Encyclopedia of Genes and Genomes (KEGG) analyses 31, we sought to delineate the biological processes and signaling pathways influenced by ART1. Employing a stringent threshold of *P* < 0.05 and False Discovery Rate (FDR) < 0.25, we identified significantly enriched pathways. To facilitate interpretation and visualization of the top 10 results, we utilized a combination of R programming and Cytoscape software, offering a comprehensive portrayal of the molecular landscape influenced by ART1 and its interacting partners. This integrative approach promises to unveil novel insights into the multifaceted roles of ART1 in cellular biology and disease pathogenesis.

### 2.2 Bayesian co-localization analysis

To delineate potential co-localization between ART1 and digestive tract tumors, we harnessed the “coloc” R package, a powerful tool for conducting co-localization analysis 32. This analysis facilitates the exploration of four hypothetical posterior probabilities, indicating whether a single variable is shared by two traits. A critical criterion for determining gene co-location is predicated on the assumption that the PPH4 score exceeds 0.8 33.

In this study, Genome-Wide Association Study (GWAS) data pertaining to Stomach Adenocarcinoma (STAD) (ebi-a-GCST90018849) and Colorectal Cancer (CRC) (ebi-a-GCST90018808) were meticulously sourced from the IEU OpenGWAS database (https://gwas.mrcieu.ac.uk/). Leveraging these invaluable datasets, we embarked on a systematic exploration of potential co-localization between ART1 and digestive tract tumors, thus shedding light on putative shared genetic factors underlying these malignancies.

### 2.3 Experimental Analysis

#### 2.3.1 Cell Culture

GC cell lines HGC-27, MKN-45, AGS, along with normal human gastric epithelial cell lines GSE, were procured from Nanjing KGI Biotechnology Company for experimental investigations. Morphological characteristics and cell density were meticulously observed under a microscope prior to initiating the experiments. The culture medium in the original flask was carefully aspirated, followed by the addition of 2 mL of Phosphate Buffered Saline (PBS). Subsequently, 1 mL of 0.25% trypsin was introduced for static digestion, with an incubation duration of 1-2 minutes. To halt the digestion process, an equivalent volume of complete culture medium was added as the 0.25% trypsin solution. The resulting cell suspension was transferred to a centrifuge tube and centrifuged at 1000 revolutions per minute (r/min) for 5 minutes. Upon removal of the supernatant, 3-4 mL of fully cultured cells were suspended and subsequently transferred to a culture flask. The cells were then incubated in a controlled environment of a 37°C cell incubator with 5% CO2 to facilitate optimal growth conditions.

#### 2.3.2 Quantitative Real-Time PCR

Total RNA was isolated from the cultured cells utilizing a high-quality RNA extraction kit, adhering to the manufacturer's instructions. Subsequently, mRNA reverse transcription was carried out using a reliable reverse transcription kit, ensuring fidelity and accuracy in the transcription process. For the evaluation of gene expression levels, a state-of-the-art q225 fluorescent quantitative PCR instrument was employed, leveraging the highly sensitive SYBR GreenMix PCR kit from Roche. The reaction conditions were meticulously specified in accordance with the manufacturer's guidelines. The thermal cycling parameters were meticulously programmed, commencing with an initial denaturation step at 95°C for 300 seconds, followed by 40 cycles of denaturation at 95°C for 10 seconds and annealing/extension at 60°C for 30 seconds. Quantitative PCR assays were conducted with 3 replicates per reaction to ensure robustness and reliability of the results. GAPDH served as the internal reference for mRNA normalization. Subsequently, gene expression levels were quantified utilizing the 2^-ΔΔCt^ method, allowing for accurate determination of relative gene expression levels compared to the internal reference. The specific sequences of the primers employed in this study are meticulously detailed in Supplementary Table 2, ensuring transparency and reproducibility in the experimental procedures.

#### 2.3.3 Western blot

The protein extraction process entails the application of a protein lysis solution to the cell lysate, meticulously carried out in an ice bath to preserve protein integrity. Subsequently, the total protein concentration is quantitatively determined utilizing the BCA method, ensuring accurate assessment of protein levels. For protein analysis, the protein samples are separated through either 10% or 12% SDS-PAGE electrophoresis, depending on the specific experimental requirements. Following electrophoresis, the proteins are transferred onto a PVDF membrane, facilitating subsequent immunoblotting procedures. The PVDF membrane is then incubated in a shaking solution containing 5% skimmed milk powder, effectively blocking non-specific binding sites and enhancing signal specificity. Following blocking, the membrane is probed with a protein-specific primary antibody, ensuring specific detection of the target protein of interest. Incubation with the primary antibody is conducted at 4°C for an extended duration, typically 13 hours, to facilitate optimal antibody binding. Subsequently, the membrane undergoes additional incubation with the corresponding secondary antibody, typically for 1.5 hours, further amplifying the signal for enhanced detection sensitivity. Following secondary antibody incubation, protein bands are visualized using an ECL luminous kit, enabling detection of antibody-bound protein bands on the membrane. Finally, images of the protein bands are captured utilizing an imaging system, ensuring accurate documentation of experimental results. Subsequent analysis and quantification of protein band intensities are performed using Image J software, facilitating data interpretation and comparison across experimental conditions. This comprehensive procedure ensures robust and reliable protein analysis, essential for elucidating molecular mechanisms underlying biological processes.

#### 2.3.4 Flow cytometry cell apoptosis assay

The overexpression plasmid for ART1 (OE-ART1) was acquired from Shanghai Jikai Biotechnology Company in China. To commence the experiment, HGC-27 cells were initially seeded in a 6-well plate and allowed to incubate for 24 hours under standard conditions of 37℃ and 5% CO2 to facilitate adherence and cellular recovery. Subsequently, the cells were transfected with either an empty plasmid (control) or the OE-ART1 plasmid, following meticulous adherence to the manufacturer's instructions for transfection protocols. During the logarithmic growth phase of HGC-27 cells, a single-cell suspension was meticulously prepared and subsequently re-seeded into a six-well plate at a density of 5×105 cells per well. The cells were then cultured in a constant-temperature incubator at 37℃ to promote optimal growth conditions. Upon reaching a cell density of 80-90%, indicative of robust cellular growth, the cells were trypsinized, washed with PBS, and suspended in 400 μL of Binding Buffer. Subsequently, the cells were stained with 5 μL of Annexin V-FITC and incubated in the dark at 4℃ for 15 minutes to facilitate annexin binding to phosphatidylserine residues exposed during early apoptosis. Following this, 10 μL of Propidium Iodide (PI) was added to the cell suspension and incubated for an additional 5 minutes in the absence of direct light. Finally, apoptosis was assessed utilizing flow cytometry, enabling quantitative analysis of apoptotic cell populations based on Annexin V-FITC and PI staining patterns. This comprehensive experimental procedure allows for the elucidation of apoptosis induction upon ART1 overexpression, providing valuable insights into its biological role in cellular processes.

#### 2.3.5 Statistical Analysis

The database used in this study was shown in Supplementary Table 3. Group differences were assessed using a *t*-test. Correlation analysis was conducted using Spearman's test. The analysis was carried out using SPSS 17.0, and significance was defined as *P* < 0.05.

## 3. Results

### 3.1 Bioinformatics analysis

#### 3.1.1 Expression of ART1 in pan-cancer

**Figure 2:**
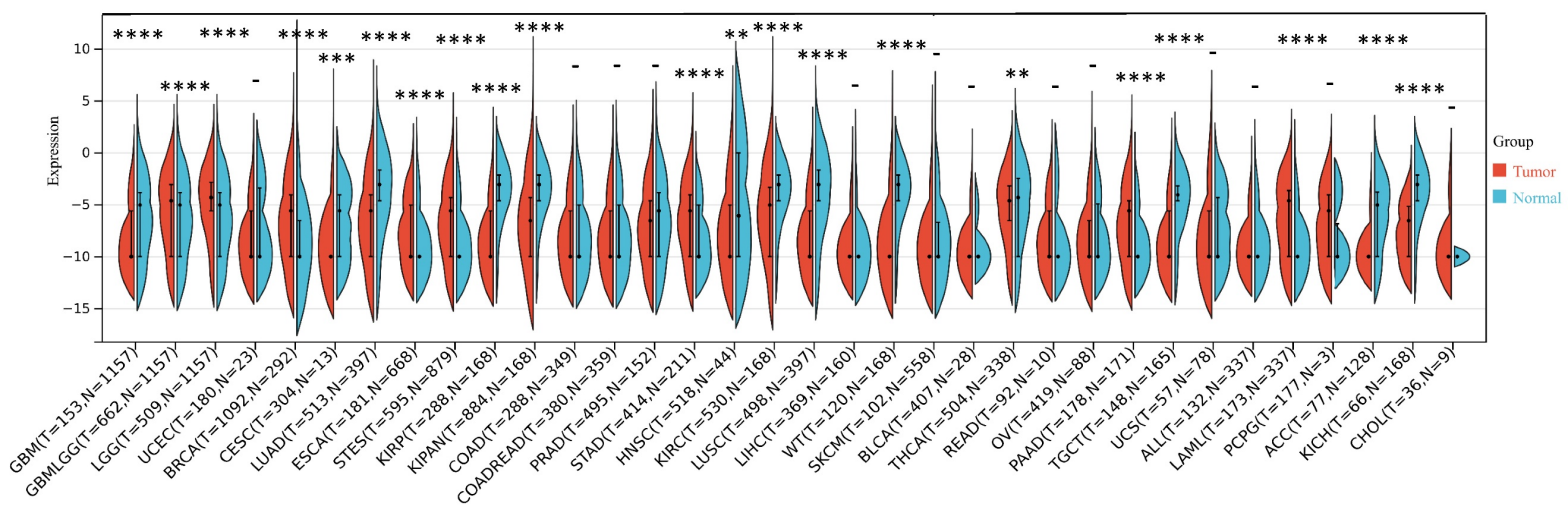
**The differential mRNA expression of ART1 in various tumors** (*P < 0.05; ** *P* < 0.01; *** *P* < 0.001 and **** *P* < 0.0001; -, not significant).

**Figure 3:**
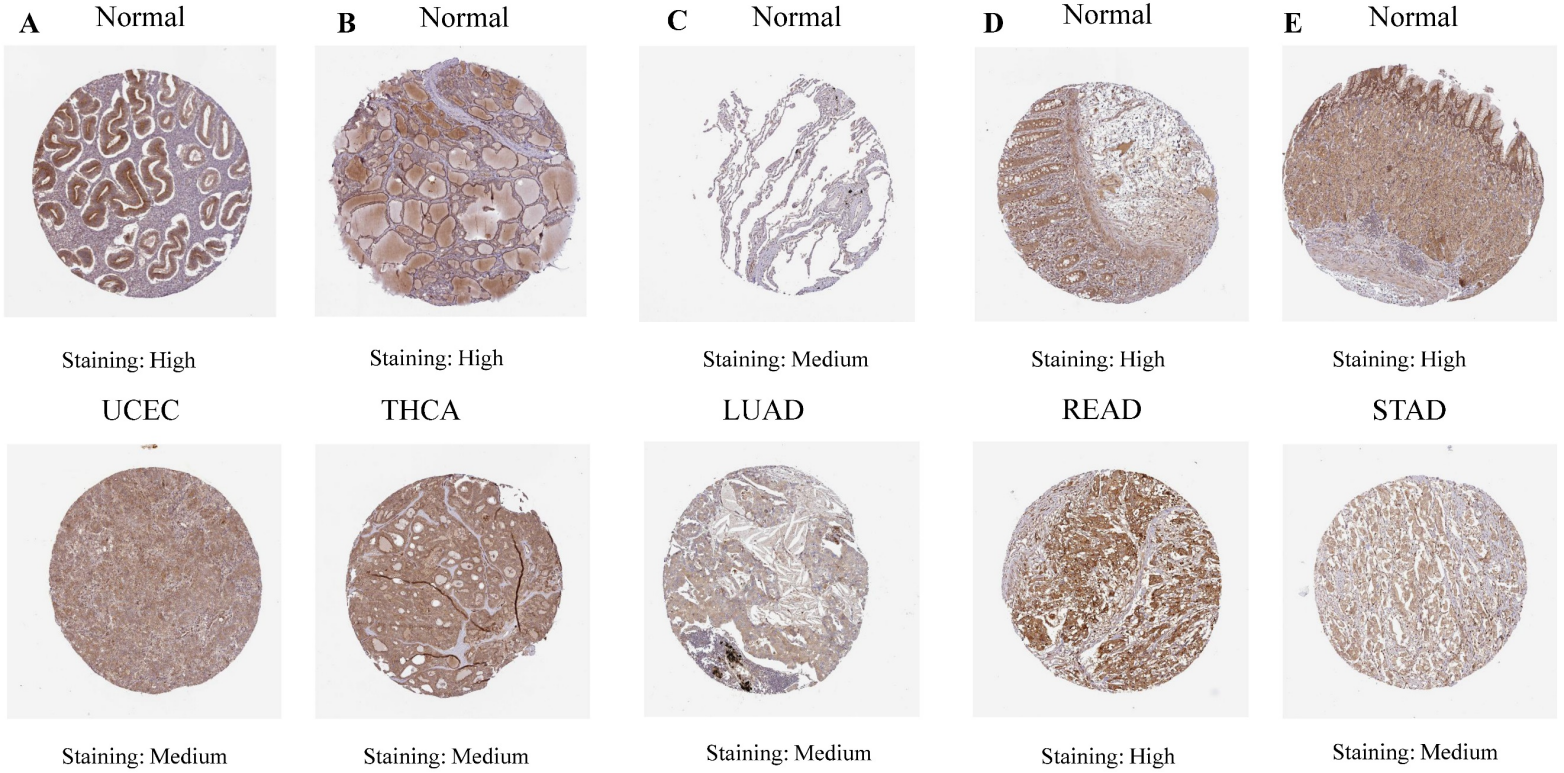

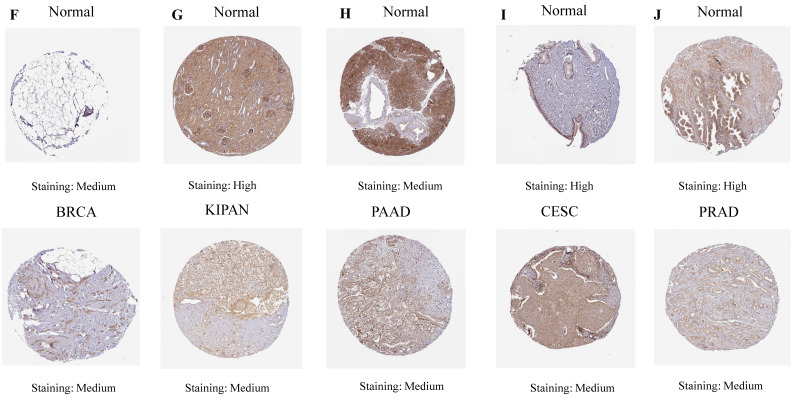
IHC images of ART1 protein expression in HPA database.

Our investigation unveiled a notable upregulation of ART1 mRNA expression in several cancer types including GBMLGG, LGG, BRCA, ESCA, STES, STAD, PAAD, and LAML (*P* < 0.05). In contrast, a pronounced downregulation of ART1 mRNA expression was observed in GBM, CESC, LUAD, KIRP, KIPAN, PRAD, HNSC, KIRC, LUSC, WT, THCA, TGCT, UCS, ACC, and KICH (*P* < 0.05) (Figure 2). Additionally, insights gleaned from the HPA delineated elevated levels of ART1 protein expression specifically in UCEC, STAD, THCA, KIPAN, CESC, and PRAD (Figure 3A-J). These compelling observations underscore the nuanced differential expression patterns of ART1 across a spectrum of cancers, suggesting a potential pivotal role for ART1 in the intricate landscape of carcinogenesis.

#### 3.1.2 Prognosis Value of ART1

**Figure 4:**
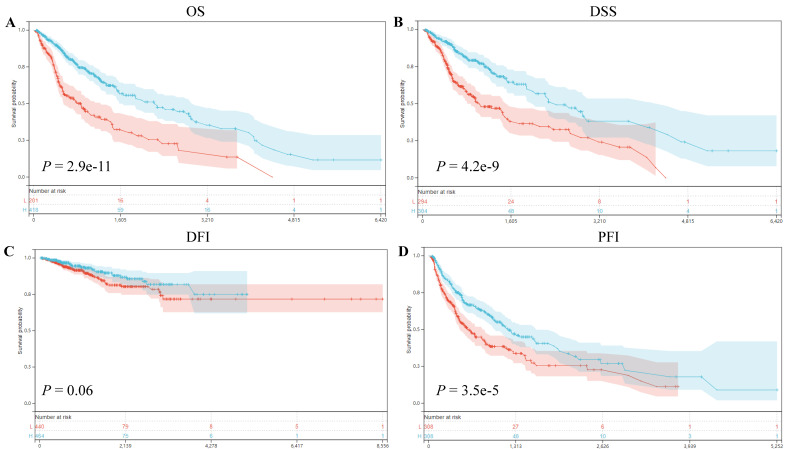
**Correlation between ART1 and Survival analysis.** (A) Kaplan-Meier analysis of ART1 expression and OS in pan cancers. (B) Kaplan-Meier analysis of ART1 expression and DSS in pan cancers. (C) Kaplan-Meier analysis of ART1 expression and DFI in pan cancers. (D) Kaplan-Meier analysis of ART1 expression and PFI in pan cancers. The red represents the low ART1 expression group, and the blue represents the high ART1 expression group.

Our prognostic analyses unveiled significant associations between ART1 expression levels and clinical outcomes in several cancer types. Specifically, elevated ART1 expression was notably linked to poorer prognoses in SKCM (*P* = 0.04, OR = 1.12, 95%CI 1.00-1.25), LAML (*P* = 0.02, OR = 1.07, 95%CI 1.01-1.13), and DLBC (*P* < 0.001, OR = 1.43, 95%CI 1.06-1.94). Conversely, augmented ART1 expression correlated with favorable prognoses in GBMLGG (*P* < 0.001, OR = 0.87, 95%CI 0.84-0.91), BLCA (*P* = 0.02, OR = 0.90, 95%CI 0.81-0.99), and KICH (*P* < 0.001, OR = 0.55, 95%CI 0.32-0.94). Concerning disease-specific survival (DSS), heightened ART1 expression significantly correlated with favorable disease-free survival (DFS) in GBMLGG (*P* < 0.001, OR = 0.87, 95%CI 0.84-0.91), KIRC (*P* = 0.02, OR = 0.94, 95%CI 0.88-0.99), BLCA (*P* = 0.02, OR = 0.86, 95%CI 0.76-0.98), and KICH (*P* = 0.02, OR = 0.58, 95%CI 0.34-1.01), thus positioning ART1 as a protective factor for GBMLG, KIRC, BLCA, and KICH. In terms of disease-free interval (DFI) analysis, our findings revealed that heightened ART1 expression significantly correlated with favorable DFI in CESC (*P* = 0.01, OR = 1.19, 95%CI 1.03-1.37), while diminished ART1 expression was associated with poorer DFI in THCA (P = 0.03, OR = 0.87, 95%CI 0.76-0.99), thus positioning ART1 as a risk factor for CESC and a protective factor for THCA. Regarding progression-free interval (PFI) analysis, our outcomes indicated that elevated ART1 expression significantly correlated with favorable PFI in SKCM (*P* < 0.001, OR = 1.17, 95%CI 1.04-1.31), whereas reduced ART1 expression was associated with poorer PFI in GBMLGG (*P* < 0.001, OR = 0.93, 95%CI 0.89-0.96), KIRC (*P* < 0.001, OR = 0.93, 95%CI 0.89-0.98), THCA (*P* = 0.01, OR = 0.89, 95%CI 0.81-0.98), and KICH (*P* < 0.001, OR = 0.66, 95%CI 0.46-0.94), positioning ART1 as a risk factor for SKCM and a protective factor for GBMLGG, KIRC, THCA, and KICH (Supplementary Figure 1). In addition, OS, DSS, DFI, and PFI Kaplan-Meier curve analysis showed that underscored ART1 as a protective factor across all cancers (Figure 4).

#### 3.1.3 Mutation landscape

**Figure 5:**
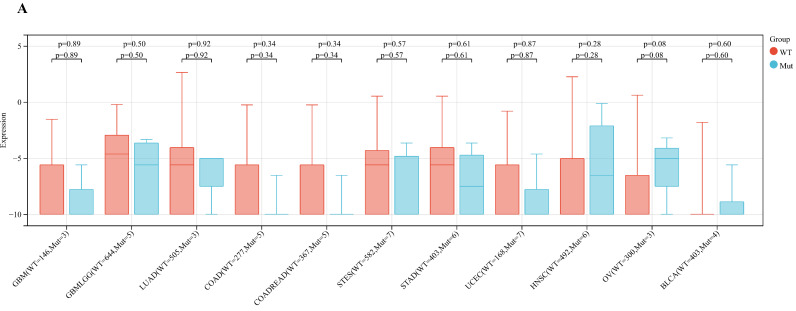

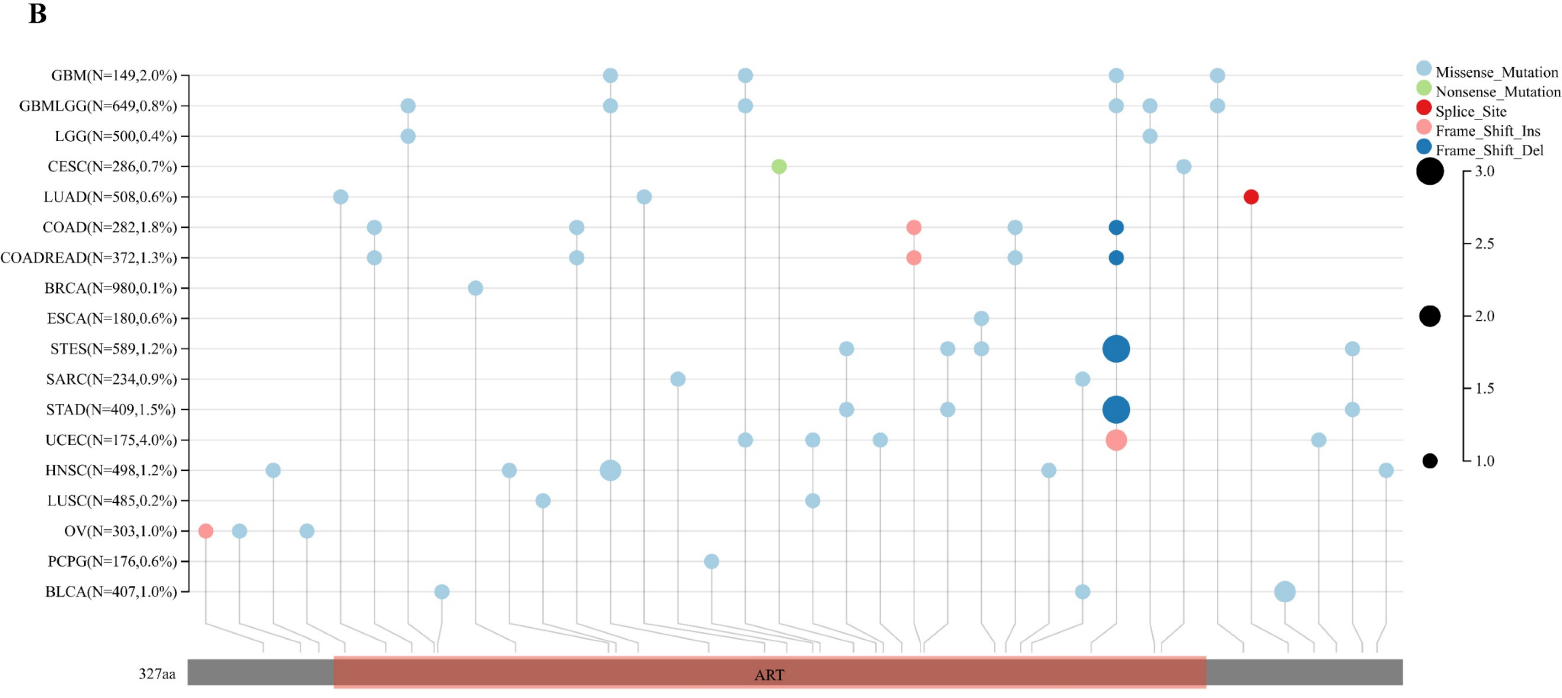

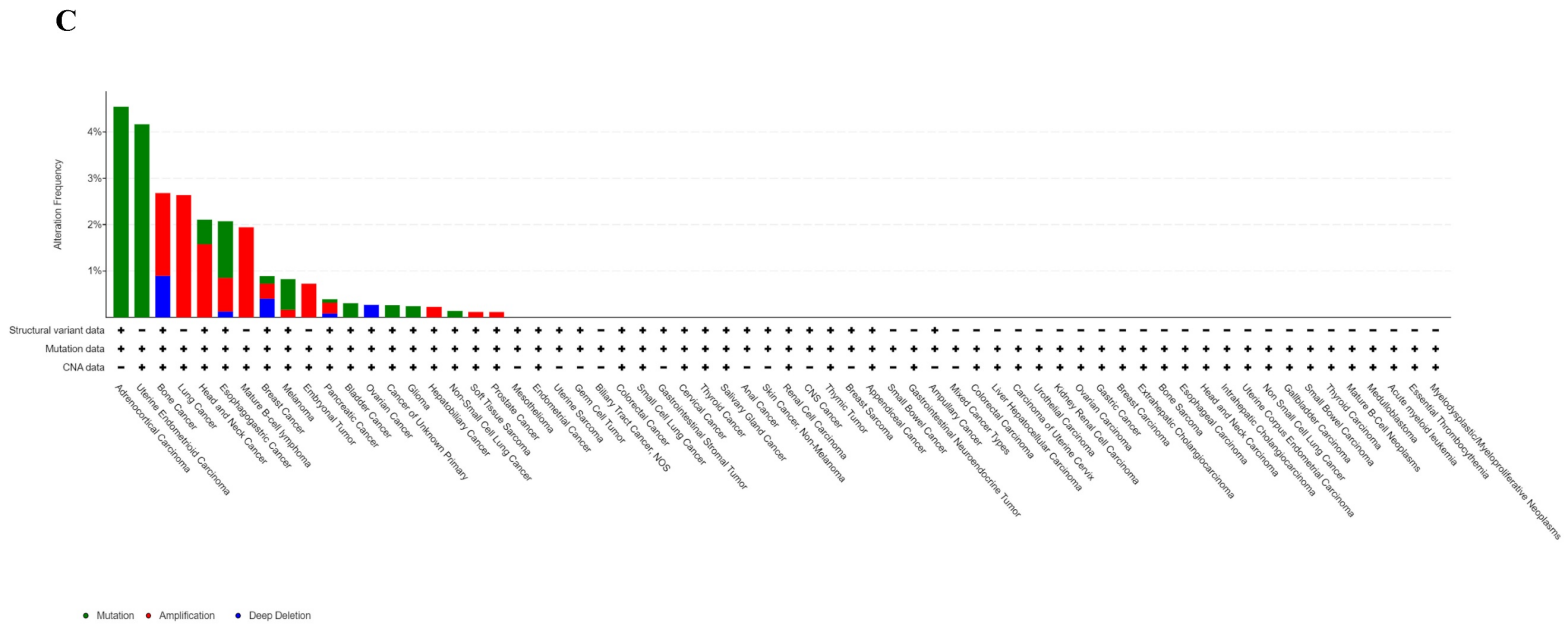
** Mutation profiles of ART1 in pan cancers.** (A) Mutation and expression analysis of ART1. (B) ART1 mutation landscape. (C) The cBioPortal application displays the frequency of ART1 mutations with mutation type in TCGA cancers.

Analyzing ART1 expression across diverse clinical samples within each tumor type yielded no discernible differences in ART1 levels among the 11 tumor types (Figure 5A). Furthermore, the acquisition of protein domain information unveiled a relatively elevated mutation rate (2%) specifically in GBM (Figure 5B). Leveraging the cBioPortal tool facilitated the assessment of the genetic modification status of ART1, revealing that mutations were predominantly prevalent in adrenocortical carcinoma (> 4%) (Figure 5C). These comprehensive analyses provide valuable insights into the genetic landscape and mutation patterns associated with ART1 across various cancer types.

#### 3.1.4 ART1 immune role identification

**Figure 6:**
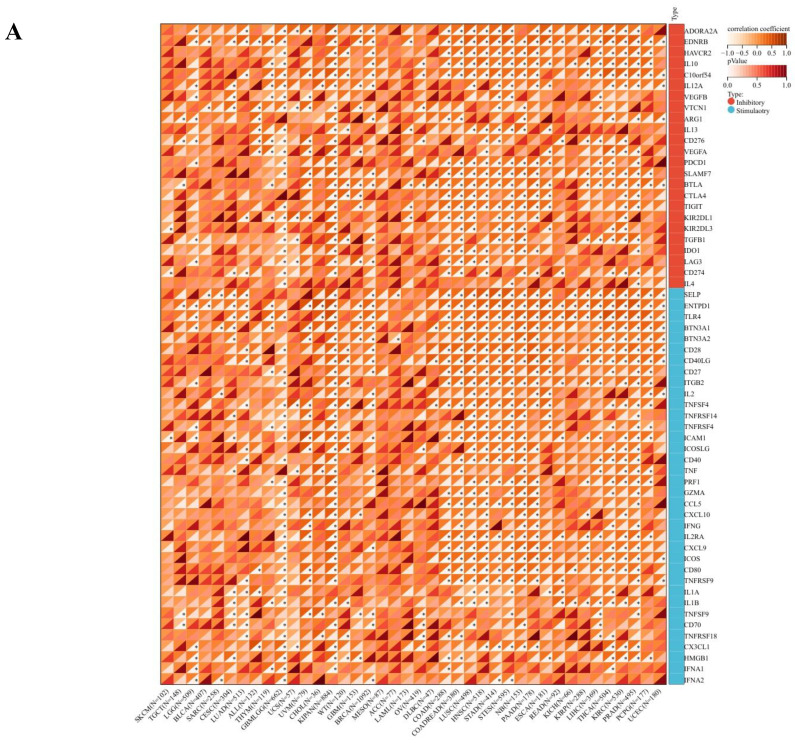

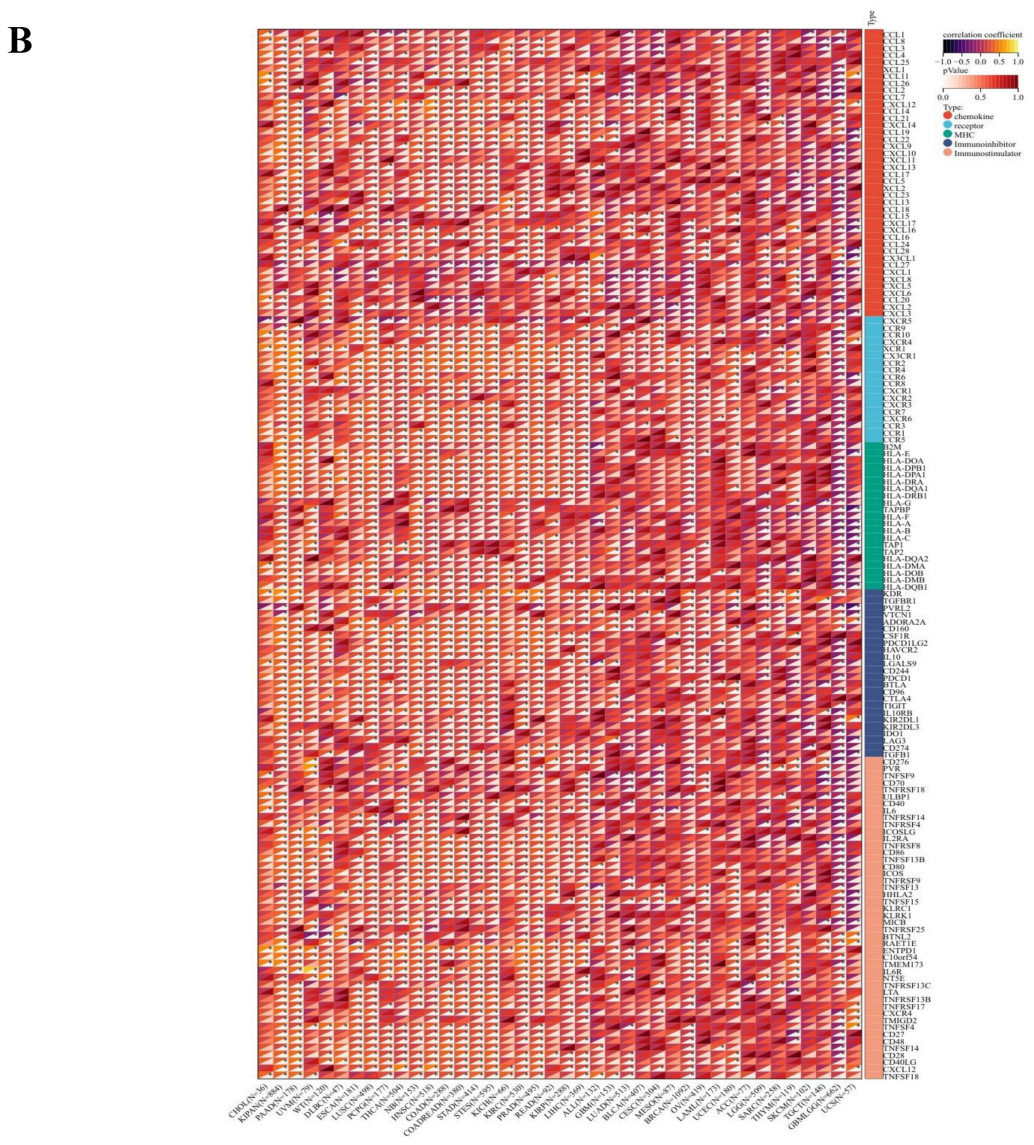
** Immune status of ART1 in pan cancers.** (A) Correlation between ART1 and immunomodulatory genes (chemokine, receptor, MHC, immune inhibitor, and immune stimulator). (B) Correlation between ART1 and immune checkpoint genes (inhibitory and stimulatory). The asterisks indicate statistically significant *P*-values calculated using spearman correlation analysis (*P < 0.05; ** P < 0.01; *** P < 0.001).

The comprehensive analysis conducted utilizing the TISMO database (http://tismo.cistrome.org/) yielded compelling results, indicating that across 64 immunotherapy cohorts, ART1 emerged as a significant predictor of immunotherapy responses in 5 specific cohorts (STAD, COAD, BRCA, LUAD, OSCC). Notably, individuals exhibiting elevated ART1 expression levels were more inclined to exhibit favorable responses to immunotherapy (*P* < 0.05) (Supplementary Figure 2), thereby accentuating the pivotal role of ART1 in the context of tumor immunotherapy. These findings underscore the potential utility of ART1 as a prognostic biomarker and therapeutic target in the realm of cancer immunotherapy. The TME represents a complex milieu intricately intertwined with both tumor progression and the immune system 34, 35. Hence, we embarked on a thorough investigation into the correlation of ART1 expression within the TME. Our comprehensive analyses revealed notable associations between ART1 and a plethora of immune-related genes, with the majority exhibiting a negative correlation with ART1 across diverse cancers (Figure 6A). Furthermore, the robust correlation observed between ART1 and immune checkpoints suggests that ART1 holds promising potential as an optimal target for tumor immunotherapy (Figure 6B). These findings underscore the pivotal role of ART1 in shaping the immunological landscape within the TME, thereby shedding light on its significance in the context of cancer immunotherapy.

Our investigation into the association between ART1 expression and immune cell infiltration encompassed analysis from three distinct sources. The results unveiled intriguing correlations: ART1 expression exhibited negative correlations with THYM (*P* = 0.03), ALL (*P* = 0.02), and GBM LGG (*P* < 0.001), while showcasing positive correlations with NB (*P* = 0.03), LAML (*P* < 0.001), WT (*P* = 0.04), PAAD (*P* = 0.001), BRCA (*P* = 0.01), KIRP (*P* = 0.01), COAD (*P* < 0.001), COADREAD (*P* < 0.001), PRAD (*P* < 0.001), LUSC (*P* < 0.001), STAD (*P* < 0.001), STES (*P* < 0.001), KIPAN (*P* < 0.001), and HNSC (*P* < 0.001) (Supplementary Figure 3A). Additionally, concerning the Immune Score, ART1 demonstrated negative correlations with CESC (*P* = 0.03), GBMLGG (*P* < 0.001), LGG (*P* < 0.001), LAML (*P* < 0.001), and THYM (*P* < 0.001), while displaying positive correlations with LIHC (*P* = 0.02), NB (*P* = 0.04), STES (*P* < 0.001), HNSC (*P* < 0.001), PAAD (*P* < 0.001), KIPAN (*P* = 0.17), COAD (*P* < 0.001), LUSC (*P* < 0.001), STAD (*P* < 0.001), and PRAD (*P* = 0.04) (Supplementary Figure 3B). Moreover, in terms of the Stromal Score, ART1 manifested negative correlations with LGG (*P* = 0.02), GBMLGG (*P* < 0.001), and LAML (*P* < 0.001), whereas displaying positive correlations with STES (*P* < 0.001), STAD (*P* < 0.001), KIRC (*P* < 0.001), LUSC (*P* < 0.001), LAML (*P* < 0.001), THCA (*P* < 0.001), COAD (*P* < 0.001), KIRP (*P* < 0.001), ESCA (*P* = 0.02), WT (*P* < 0.001), PAAD (*P* < 0.001), KIPAN (*P* < 0.001), BRCA (*P* < 0.001), PRAD (*P* < 0.001), COADREAD (*P* < 0.001), HNSC (*P* < 0.001), and LIHC (*P* < 0.001) (Supplementary Figure 3C). These intricate correlations provide valuable insights into the multifaceted interplay between ART1 expression and immune cell infiltration across diverse cancer types.

**Figure 7:**
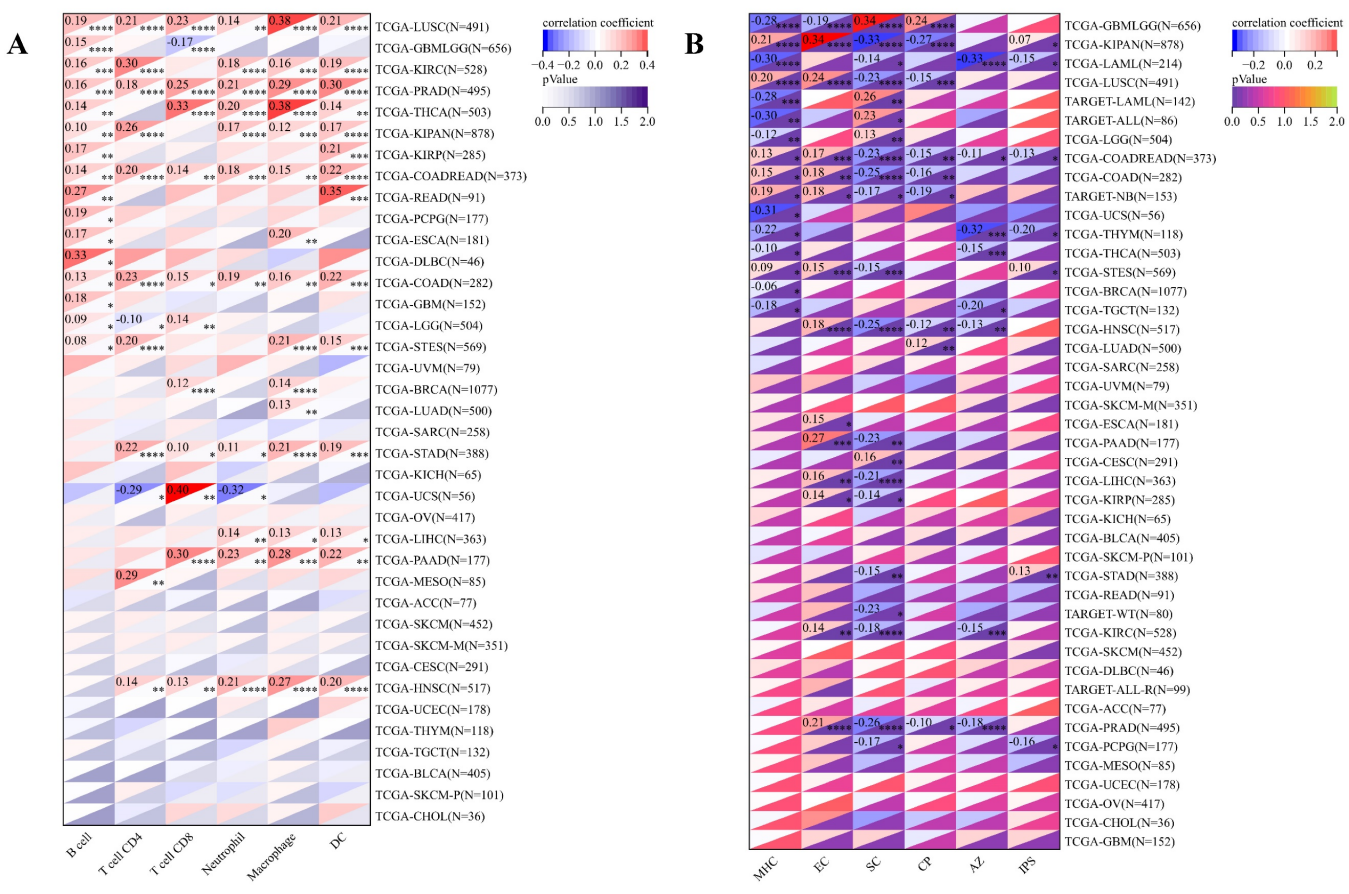

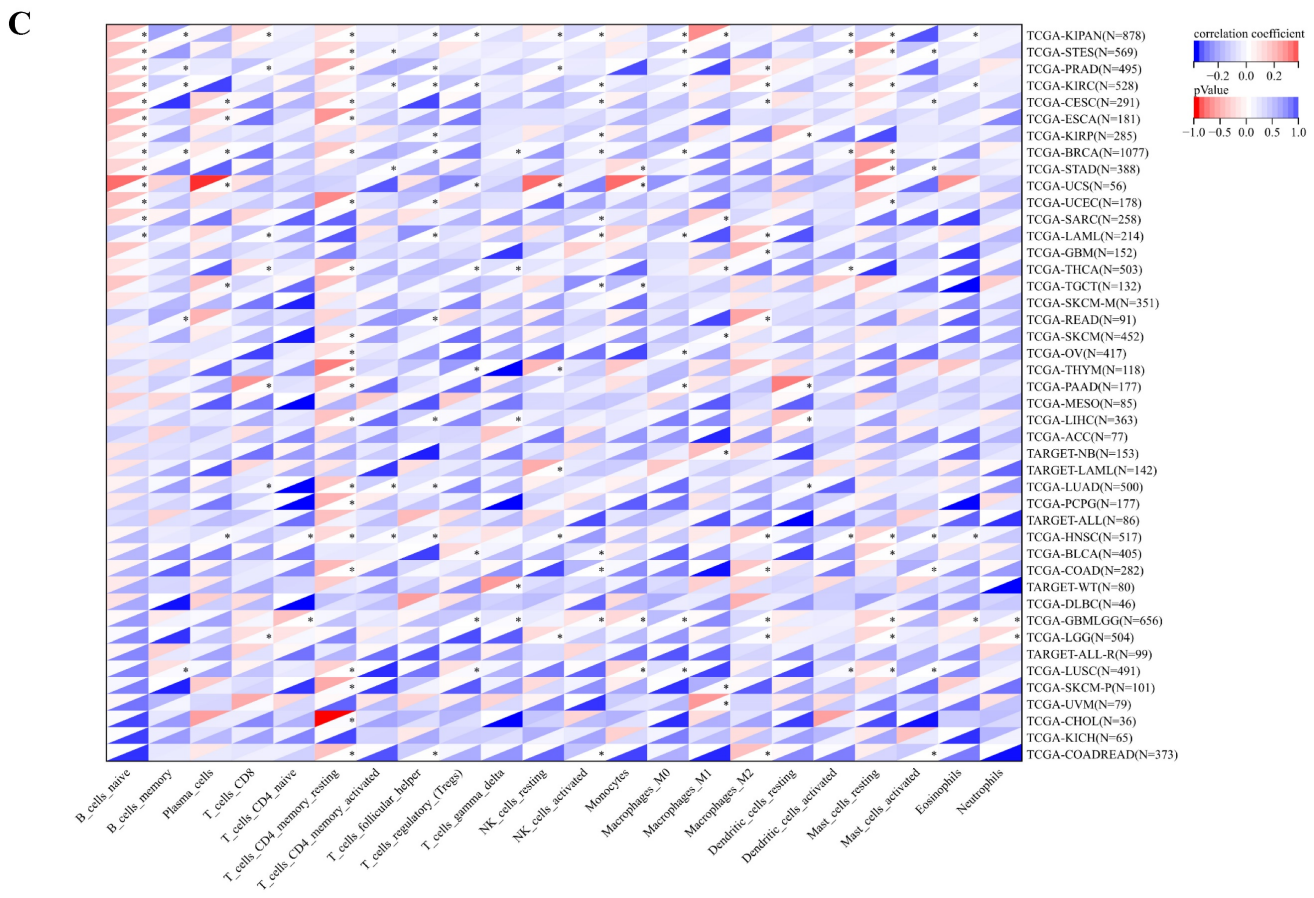
** Immune cell infiltration state of ART1 calculated by CIBERSORT, TIMER, and IPS Algorithm in pan cancers.** (A) Immune cell infiltration state of ART1 calculated by TIMER Algorithm in pan cancers. (B) Immune cell infiltration state of ART1 calculated by IPS Algorithm in pan cancers. (C) Immune cell infiltration state of ART1 calculated by CIBERSORT Algorithm in pan cancers. The asterisks indicate statistically significant P-values calculated using spearman correlation analysis (*P < 0.05; ** P < 0.01; *** P < 0.001, and **** P < 0.0001).

**Figure 8:**
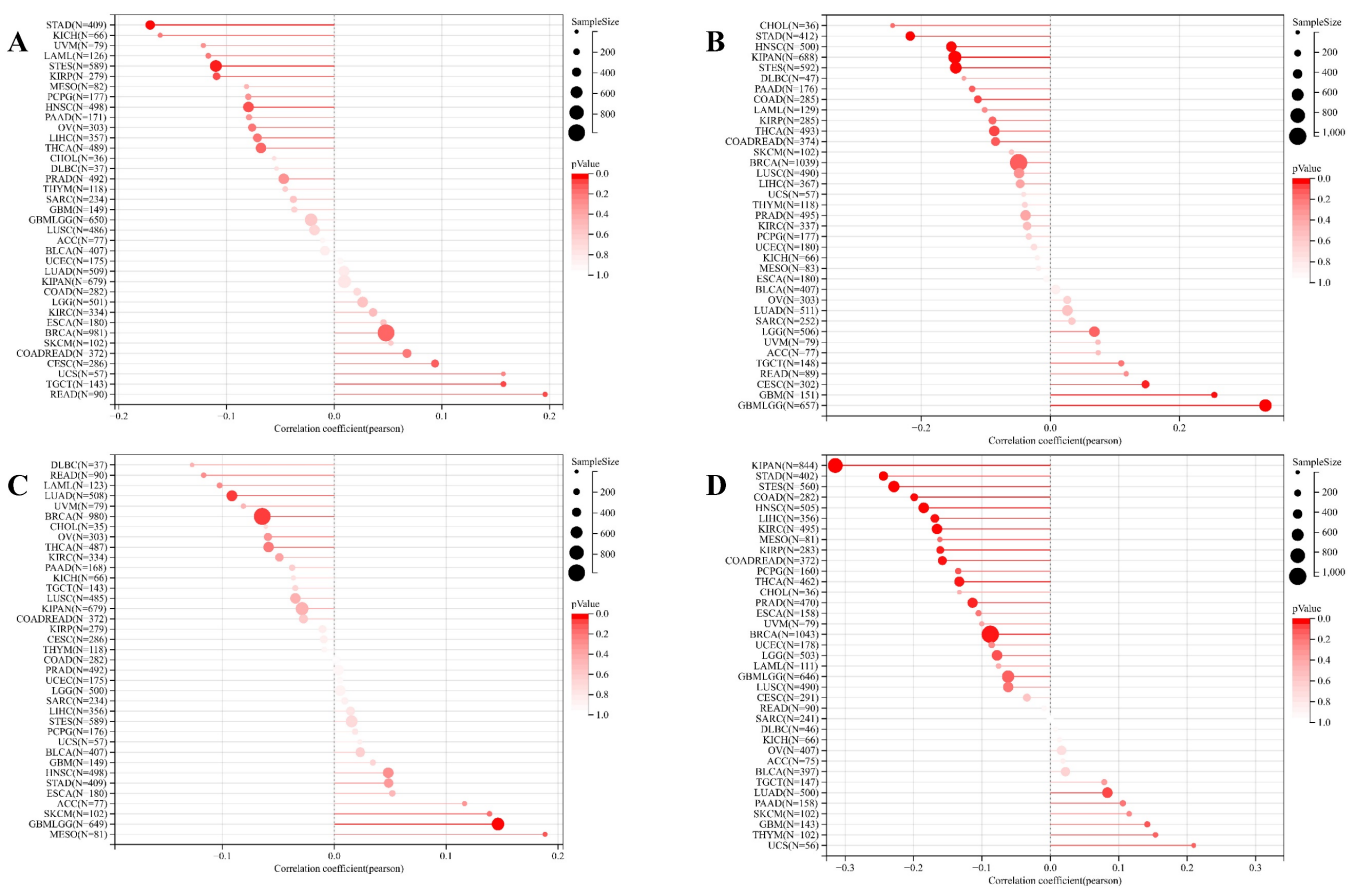
** Correlation of ART1 with TMB, MSI, MATH and purity.** (A) Lollipop picture of correlation between ART1 expression and TMB. (B) Lollipop picture of correlation between ART1 expression and MSI. (C) Lollipop picture of correlation between ART1 expression and MATH. (D) Lollipop picture of correlation between ART1 expression and purity.

Concurrently, we uncovered a notable correlation between ART1 expression and immunologic infiltration spanning 22 cancers. Specifically, 17 cancers (BRCA, ESCA, STES, KIRP, KIPA, COAD, COAD READ, PRAD, STAD, HNSC, KIRC, LUSC, LIHC, WT, NB, PAAD, LAML) exhibited a significant positive correlation, while 5 cancers (GBMLG, LGG, LAML, THYM, ALL) displayed a significant negative correlation. Utilizing data from the TIMER database, we discerned a positive correlation between ART1 expression and the infiltration levels of B cells, Macrophages, CD8+ T cells, and DCs, while noting a negative correlation with the infiltration levels of CD4+ T cells and Neutrophils (Figure 7A). Additionally, employing the IPS Algorithm, we observed a negative correlation of ART1 with MHC, SC, CP, AZ, and IPS, alongside a positive correlation with EC (Figure 7B). Further analysis via CIBERSORT results delineated a positive correlation between ART1 expression and naïve B cells, Plasma cells, CD8+ T cells, memory resting CD4+ T cells, resting NK cells, and Macrophages, while revealing a negative correlation with memory B cells, memory activated CD4+ T cells, and Tregs (Figure 7C). Given the sensitivity of TMB, MSI, MATH, and purity as indicators for immune checkpoint inhibitors 36, we delved into their associations with ART1 expression. Remarkably, ART1 expression exhibited a negative correlation with TMB in STES and STAD (Figure 8A). Moreover, positive correlations emerged between ART1 expression and MSI in seven cancer types, including GBM, GBMLGG, and CESC, while negative correlations were observed in STES, KIPAN, STAD, and HNSC (Figure 8B). Additionally, ART1 expression showcased a negative correlation with MATH in LUAD and BRCA, and a positive correlation in GBMLGG (Figure 8C). Furthermore, ART1 expression was negatively associated with tumor purity in 12 cancers, encompassing COAD, COADREAD, BRCA, STES, KIRP, KIPAN, STAD, PRAD, HNSC, KIRC, LIHC, and THCA (Figure 8D). These intricate correlations unveil the multifaceted interplay between ART1 expression and crucial immune-related parameters across various cancer types, underscoring its potential significance in the realm of cancer immunotherapy.

#### 3.1.5 Correlation Analysis between ART1 Expression and RNA modification genes

The expression of m1A genes displayed a positive association with ART1 across various cancers, including UVM, PRAD, CESC, SARC, READ, KIPA, and GBM LGG. Conversely, in WT, COAD, and STAD, ART1 exhibited a negative correlation with m1A genes. Notably, the association between ART1 expression and m5C genes remained consistent across different cancers.

**Figure 9:**
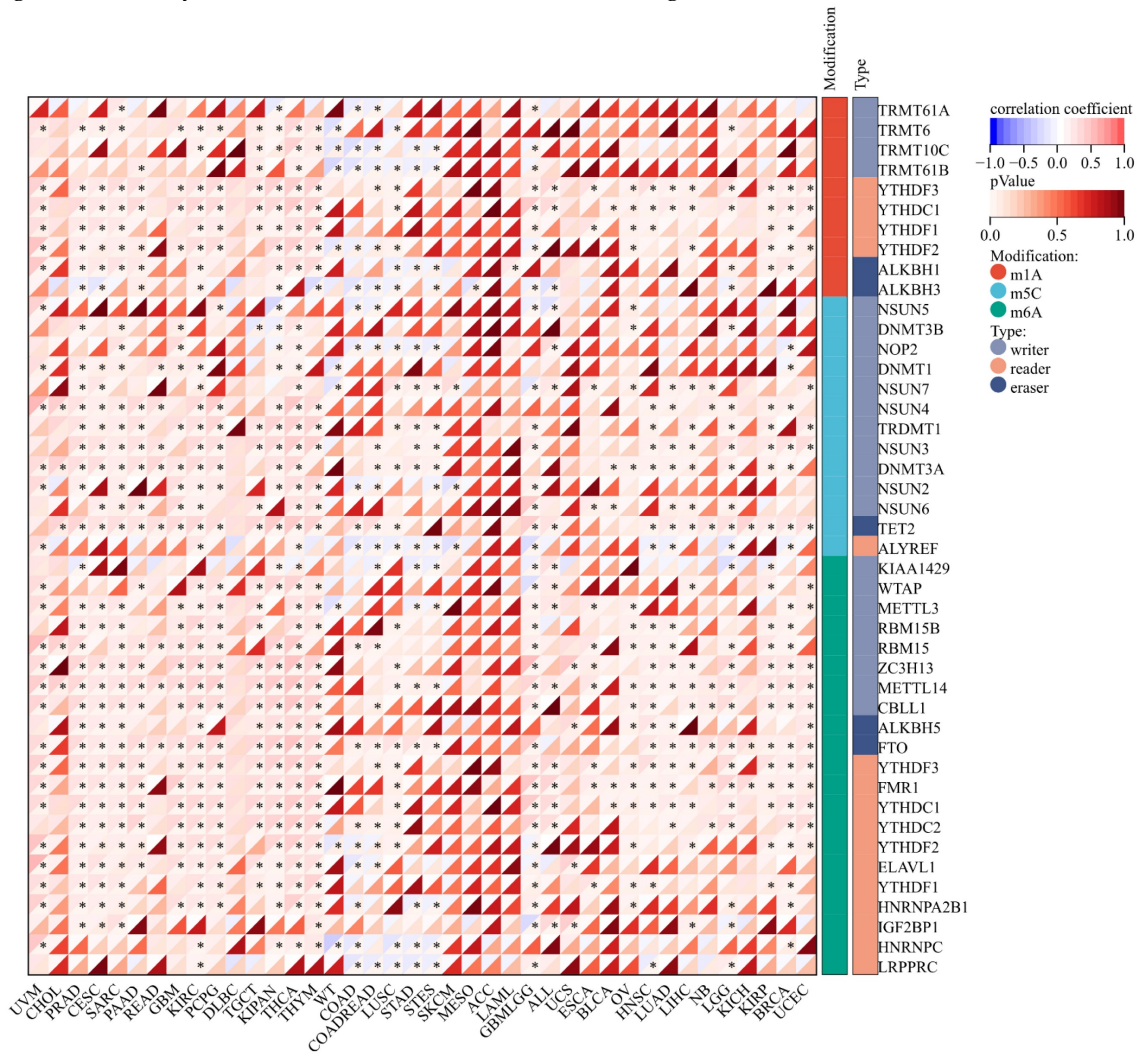
**Correlation between ART1 and RNA modification genes in pan cancers.** The asterisks indicate statistically significant P-values calculated using spearman correlation analysis (*P < 0.05; ** P < 0.01; *** P < 0.001).

Remarkably, ART1 demonstrated significant associations with m6A gene expressions in several cancers, such as UVM, PRAD, CESC, SARC, PAAD, GBM, KIRC, PCPG, TGCT, KIPAN, THYM, LUSC, GBMLG, KIRP, BRCA, and UCEC (Figure 9). These intricate associations highlight the potential regulatory role of ART1 in modulating diverse RNA modification pathways across various cancer types, suggesting its involvement in the complex landscape of cancer pathogenesis and progression.

#### 3.1.6 Relationship between ART1 and clinical features, tumor stemness

**Figure 10:**
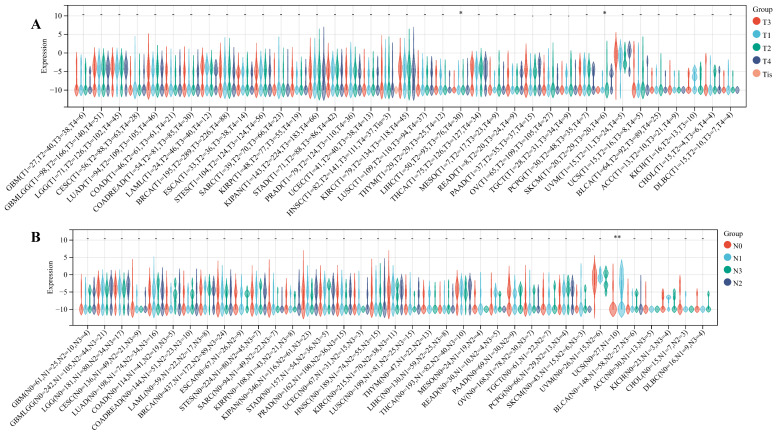

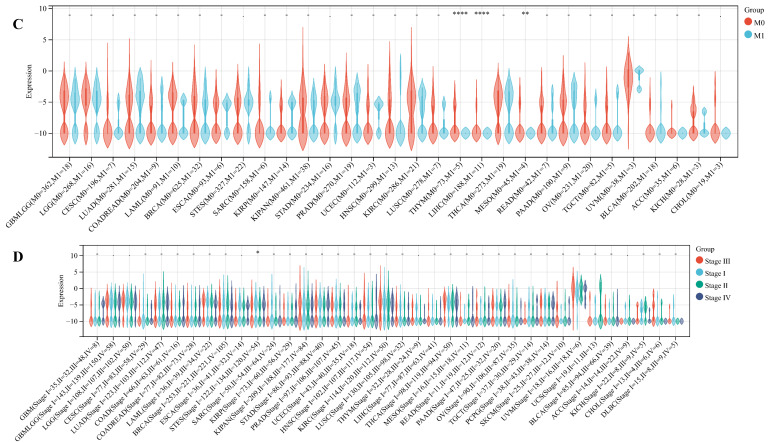

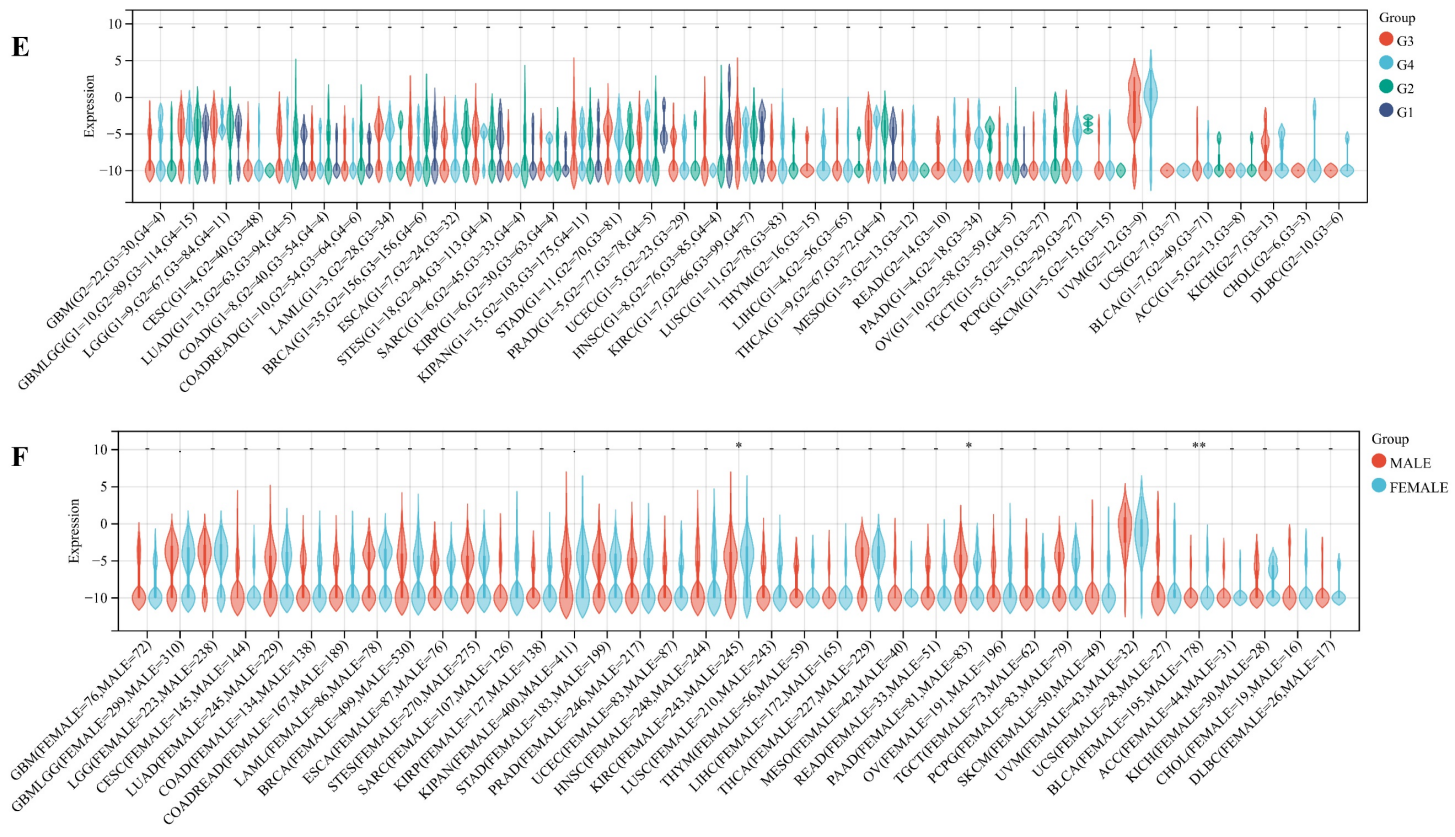
**Relationship between ART1 and clinical features in pan cancers.** (A) The relationship between ART1 and T in pan cancers. (B) The relationship between ART1 and N in pan cancers. (C) The relationship between ART1 and M in pan cancers. (D) The relationship between ART1 and Stage in pan cancers. (E) The relationship between ART1 and Grade in pan cancers. (F) The relationship between ART1 and Gender in pan cancers. The asterisks indicate statistically significant P-values calculated using spearman correlation analysis (*P < 0.05; ** *P* < 0.01; *** *P* < 0.001, and **** *P* < 0.0001; -, not significant).

We delved into the correlation between ART1 expression and clinicopathological characteristics, including gender, stage, grade, and TNM, utilizing clinical data from diverse tumor samples. Our analysis revealed significant associations of ART1 expression with various parameters. Specifically, ART1 expression correlated with T and N status in LIHC (*P* < 0.05) and SKCM (*P* < 0.05), and with M status in THYM (*P* < 0.0001), LIHC (*P* < 0.0001), and MESO (*P* < 0.01) (Figure 10A-C). Additionally, a significant correlation was observed between ART1 expression and cancer stage in STES (*P* < 0.05) (Figure 10D). However, ART1 did not exhibit significant differences in relation to tumor grade across pan cancers (Figure 10E). Furthermore, our analysis revealed associations between ART1 expression and gender in KIRC (*P* < 0.05), PAAD (*P* < 0.05), and BLCA (*P* < 0.01) (Figure 10F).

**Figure 11:**
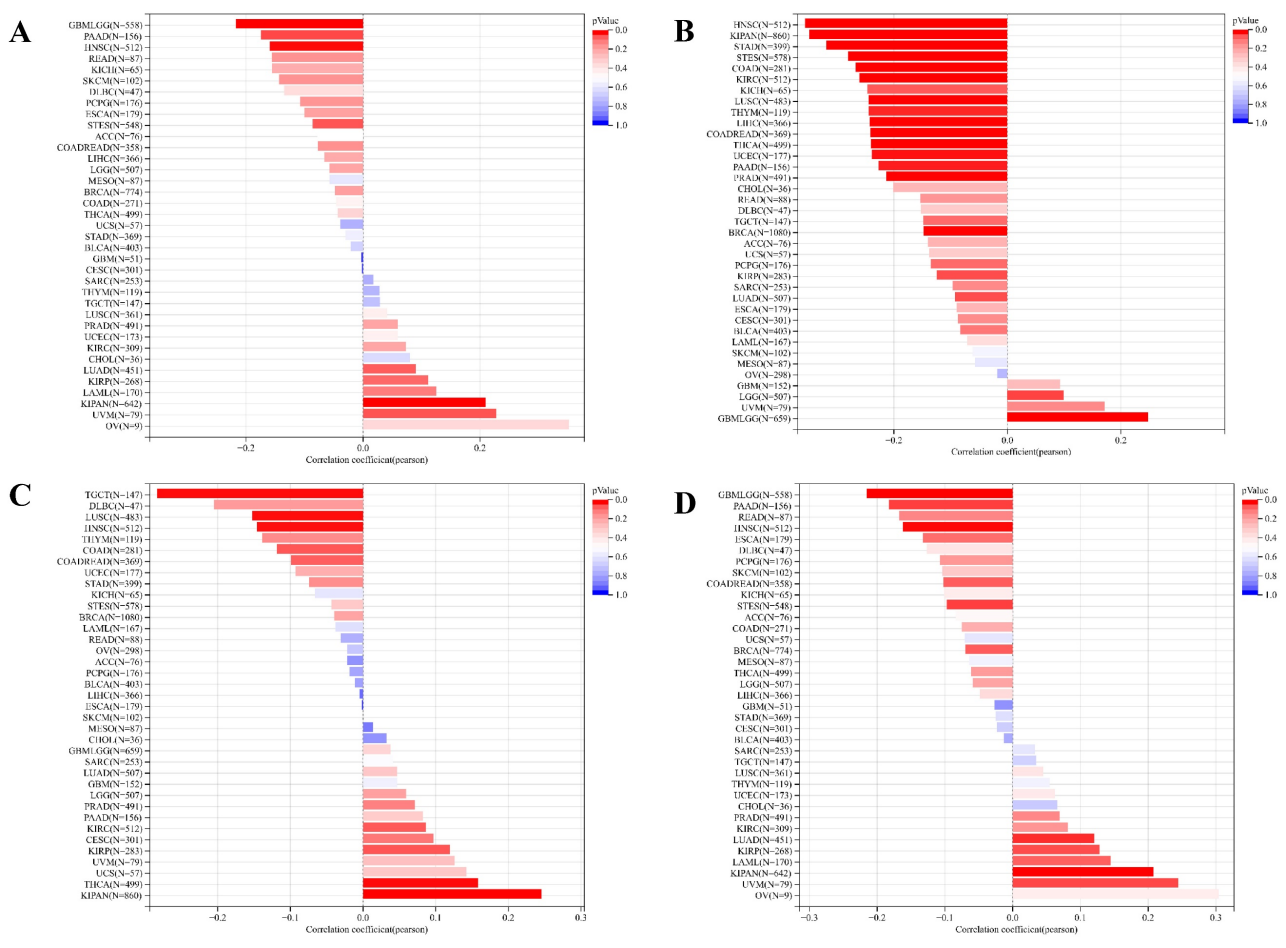

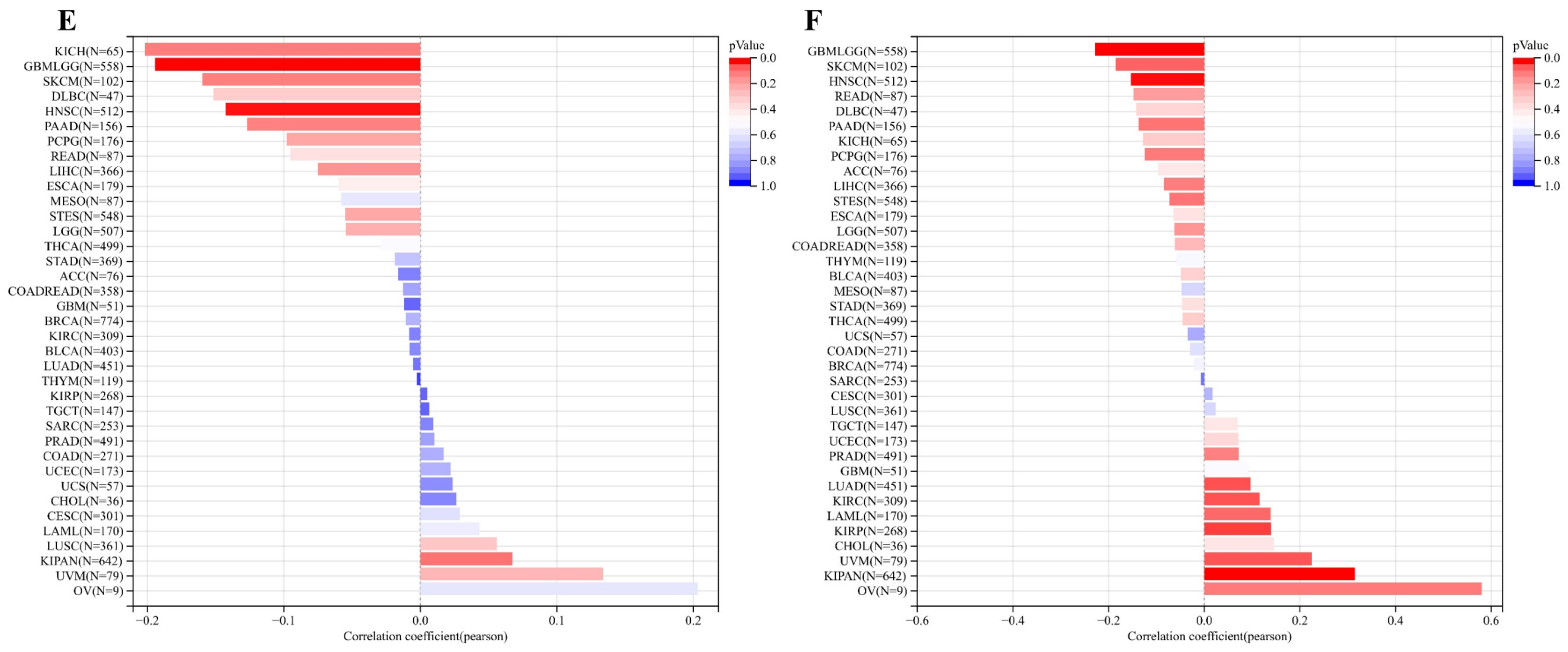
**Analysis of the relationship between ART1 and tumor stemness.** (A) Histogram of correlation between ART1 and DNAss in pan cancers. (B) Histogram of correlation between ART1 and RNAss in pan cancers. (C) Histogram of correlation between ART1 and EREG.EXPss in pan cancers. (D) Histogram of correlation between ART1 and EREG-METHss in pan cancers. (E) Histogram of correlation between ART1 and DMPss in pan cancers. (F) Histogram of correlation between ART1 and ENHss in pan cancers.

As cancer progresses, tumor cells undergo phenotypic changes, resembling progenitor and stem cells. Assessing tumor stemness using RNA-based metrics is crucial in understanding cancer biology. Our exploration of the correlation between ART1 and six dimensions of tumor stemness unearthed intriguing associations. Specifically, we found a positive correlation of ART1 with DNAss in KIPAN and UVM, while displaying a negative correlation in GBMLGG, STES, HNSC, and PAAD (Figure 11A). Similarly, ART1 exhibited a positive correlation with RNAss in GBMLGG and LGG (Figure 11B).

Moreover, ART1 demonstrated associations with EREG.EXPss, showcasing a positive correlation in KIRP, KIPAN, and THCA, and a negative correlation in COAD, HNSC, LUSC, and TGCT (Figure 11C). Our findings further elucidated associations between ART1 expression and EREG-METHss, DMPss, and ENHss (Figure 11D-F). These intricate correlations highlight the potential involvement of ART1 in modulating tumor stemness dynamics, shedding light on its role in cancer progression and metastasis.

#### 3.1.7 Functional Enrichment Analysis

**Figure 12:**
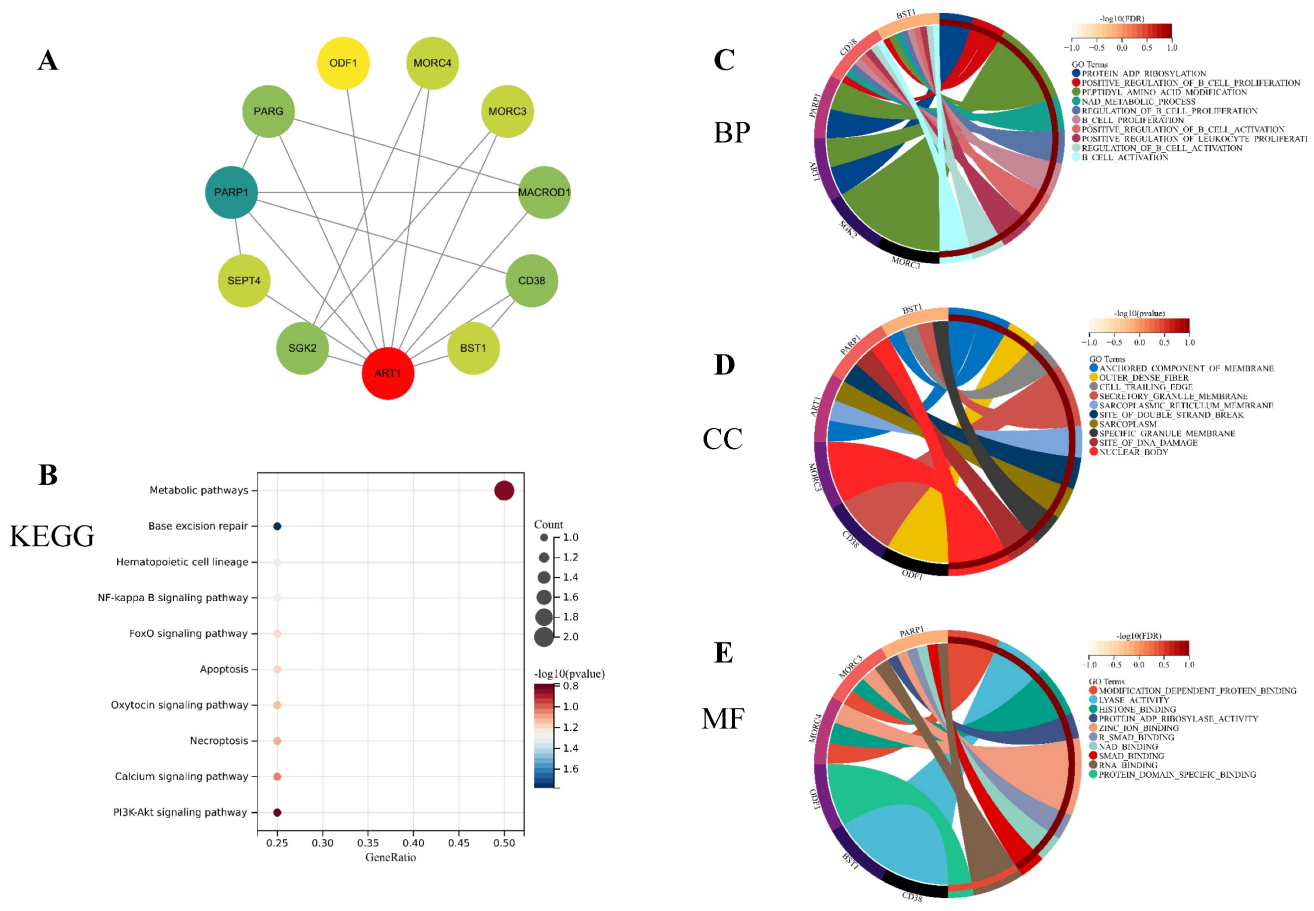
** Enrichment analysis of genes related to ART1** (top 10 enrichment were visualized). (A) Protein-protein interaction network of ART1 related genes. (B) KEGG pathway enriched with ART1 related genes. (C) BP enrichment with ART1 related genes. (D) CC enrichment with ART1 related genes. (E) MF enrichment with ART1 related genes.

To unravel the potential mechanisms underlying ART1, we constructed a PPI network for ART1 (Figure 12A). Subsequently, GO and KEGG enrichment analyses were conducted for genes associated with ART1. The KEGG analysis unveiled significant involvement of the NF-kB, Foxo, and PI3K-Akt signaling pathways in mediating the tumorigenic effects of ART1 (Figure 12B). In terms of Biological processes (BP), enrichment was predominantly observed in the regulation of B cell proliferation, B cell activation, and positive regulation of B cell activation (Figure 12C). As for Cellular components (CC), enrichment primarily occurred in the cell trailing edge, sarcoplasm, and nuclear body (Figure 12D). Moreover, Molecular functions (MF) were mainly enriched in lyase activity, SMAD binding, and RNA binding (Figure 12E). Collectively, these findings suggest that ART1 contributes to tumorigenesis by modulating the immune response, particularly through its involvement in the immunoregulatory effects of the B lymphocyte pathway.

### 3.2 Bayesian co-localization analysis

**Figure 13:**
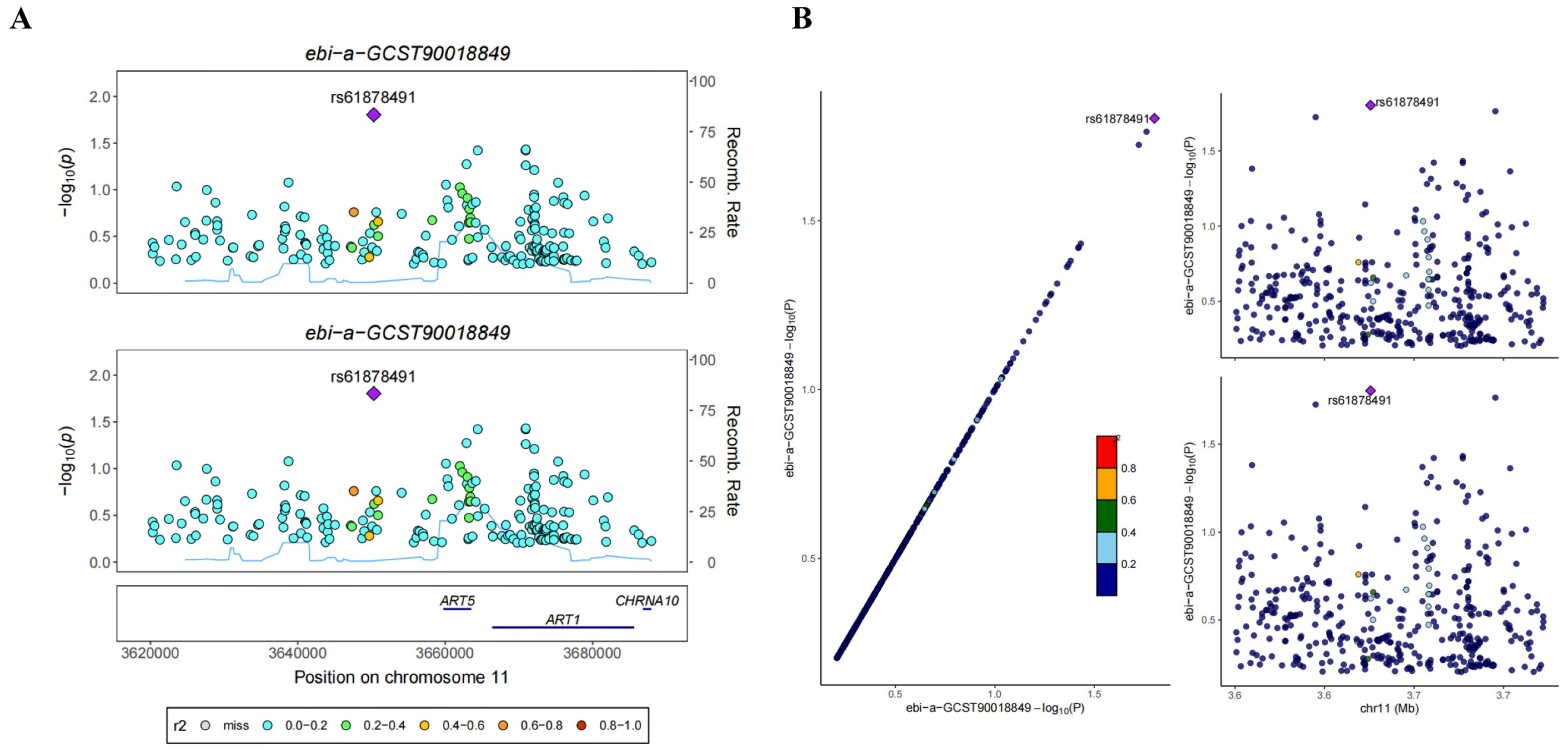

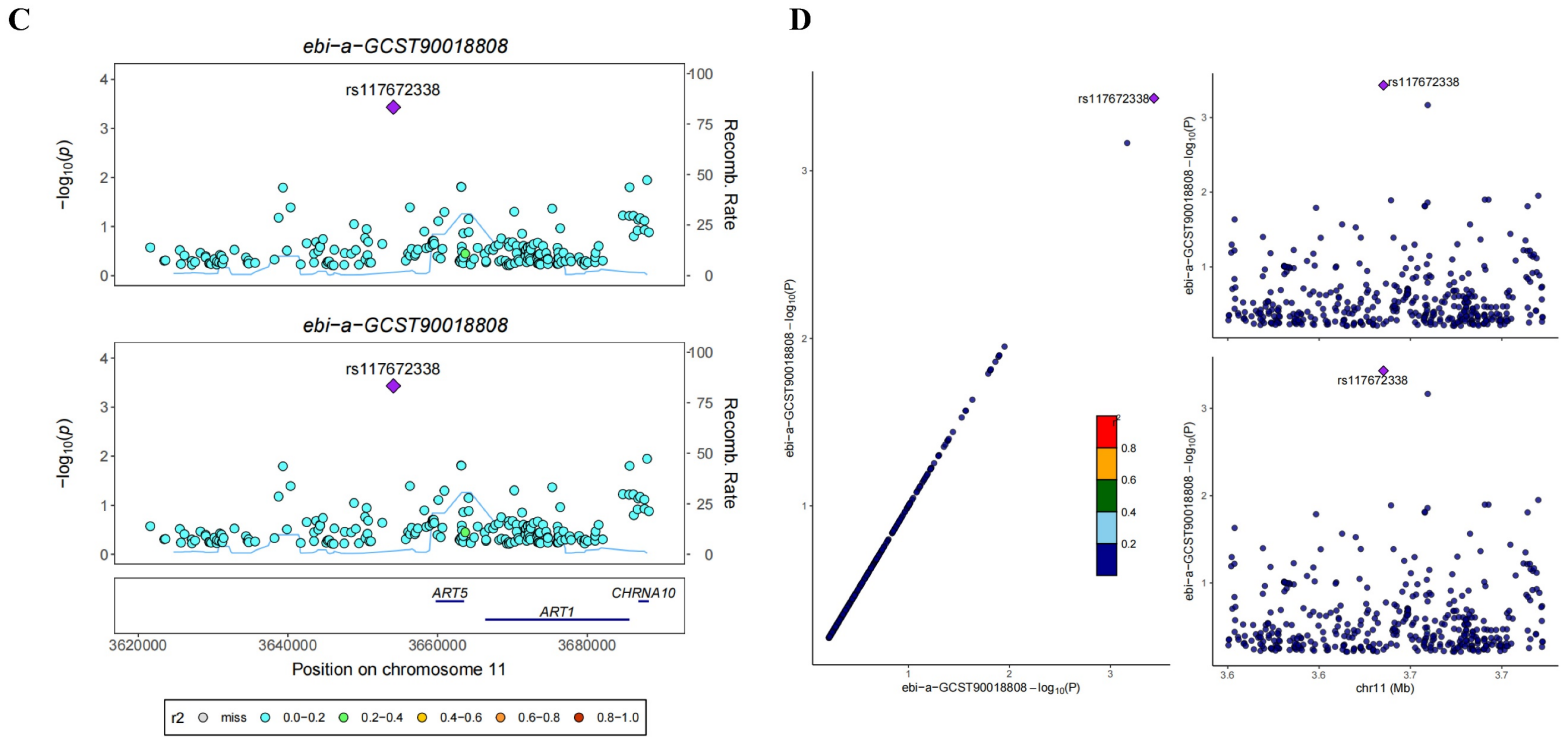
** Bayesian co-localization analysis.** (A-B) Bayesian co-localization analysis of ART1 and STAD. (C-D) Bayesian co-localization analysis of ART1 and CRC.

In the GWAS co-location analysis of ART1 and STAD, rs61878491 emerges as colocated with a posterior probability of 0.82, as depicted in Figure 13A-B. This finding suggests a potential genetic overlap between ART1 and STAD, underscoring the importance of further investigation into the shared genetic mechanisms underlying these conditions. Similarly, in the GWAS co-location analysis of ART1 and CRC, rs117672338 is identified as colocated with a posterior probability of 0.89. Notably, both variants are located on chromosome 11, as illustrated in Figure 13C-D. This observation hints at a potential genetic link between ART1 and CRC, warranting deeper exploration into their shared genetic loci and their implications in colorectal cancer pathogenesis.

### 3.3 Experimental analysis

**Figure 14:**
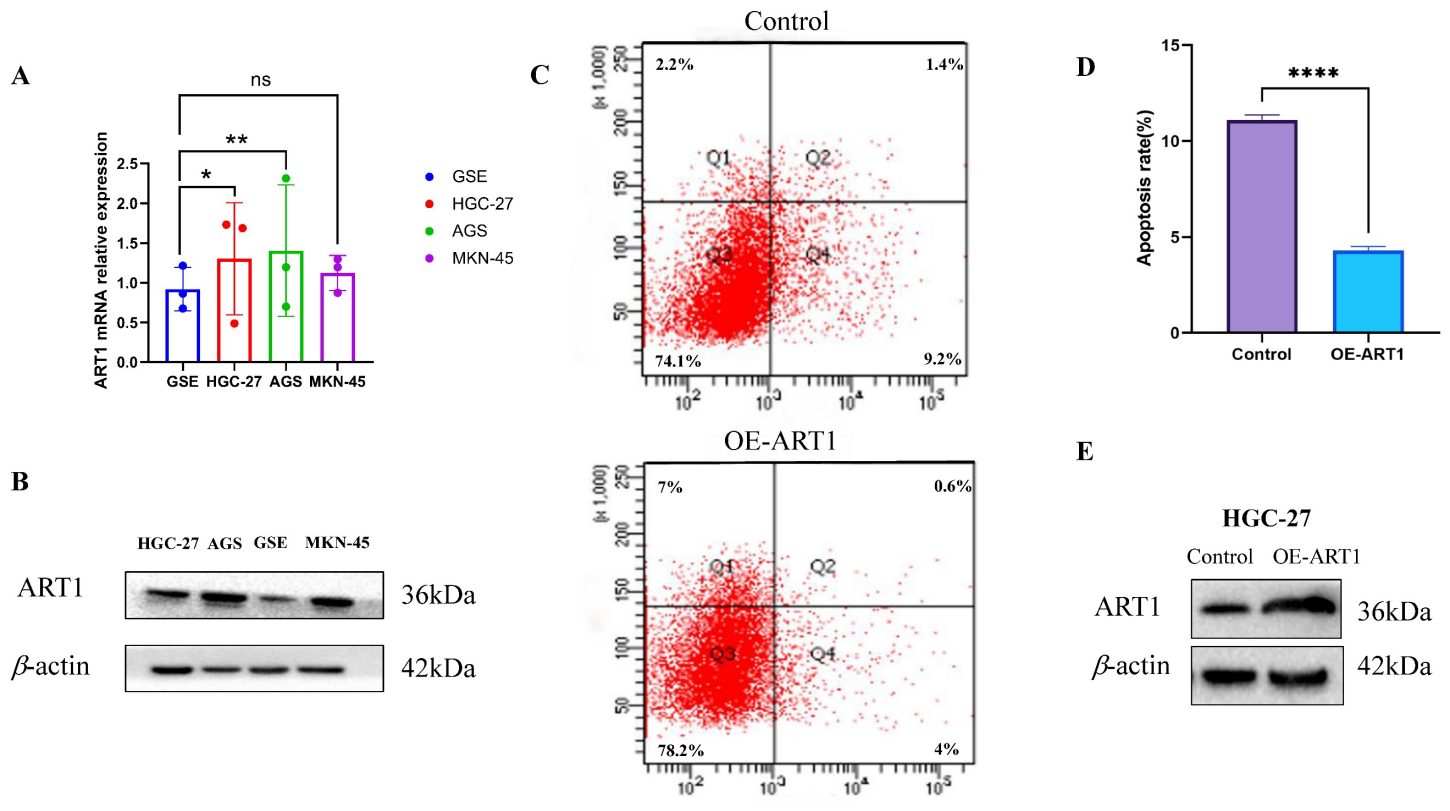
** Experimental Validation of ART1 in GC cells.** (A) mRNA expression level of ART1 in HGC-27, MKN-45, AGS and GSE (*P < 0.05; ** P < 0.01; ns, not significant). (B) Protein level of ART1 in HGC-27, MKN-45, AGS and GSE. (C-D) Apoptosis rate in HGC-27 by flow cytometry (**** P < 0.0001). (E) Protein level of OE-ART1 in the control group and OE-ART1 group.

The findings unveiled a notable upregulation in mRNA expression levels of ART1 in MKN-45, AGS, and HGC-27 cell lines in comparison to GSE (Figure 14A). Concurrently, an increase in protein levels was observed in the GC cell lines MKN-45, AGS, and HGC-27 relative to GSE (Figure 14B). Furthermore, apoptosis assessment via flow cytometry in HGC-27 cells revealed an apoptosis rate of 10.6% in the control group and 4.6% in the OE-ART1 group, as demonstrated in Figure 14C-D. Notably, the apoptosis level in HGC-27 cells was significantly diminished in the OE-ART1 group compared to the control group (*P* < 0.0001), indicating that overexpression of ART1 impedes apoptosis in HGC-27 cells. These findings underscore the potential role of ART1 in promoting cancer and suggest its utility as a prognostic biomarker in GC. Figure 14E showed that the expression of ART1 protein in HGC-27 cells of OE-ART1 group is significantly increased.

## 4. Discussion

Numerous studies have underscored the involvement of ART1 in tumorigenesis and progression. Elevated expression levels of ART1 have been noted in colorectal cancer and glioblastoma, with its heightened expression correlating with unfavorable prognosis 37. Furthermore, ART1 is implicated in bolstering the signal transduction pathways associated with epithelial-mesenchymal transition and cell proliferation. In a murine model of colorectal cancer boasting a robust immune milieu, inhibition of ART1 has demonstrated efficacy in impeding tumor growth 38. The target receptor of ART1, namely the P2X7 receptor, is expressed across various immune cell subsets, including T cells, and plays a pivotal role in orchestrating inflammatory responses and anti-tumor immunity 39. Nevertheless, despite these insights, a noticeable gap persists in our understanding of ART1 expression across diverse cancers, particularly concerning its implications for patient prognosis, survival, and its interplay within the TIME. Comprehensive analysis encompassing the entire gene set of various tumor types holds promise in uncovering genes intricately linked with cancer onset and progression, thereby furnishing invaluable insights for cancer diagnosis and therapeutic interventions.

In this comprehensive study, we conducted a thorough assessment of the prognostic implications of ART1 across a diverse spectrum of cancers, shedding light on its potential as a robust prognostic biomarker for various malignancies. Recognizing the pivotal role of genomic mutations in shaping the TME and driving tumor progression 40, we delved into the mutational landscape and identified a notably heightened mutation burden in GBM. Leveraging the powerful cBioPortal tool, we meticulously explored the mutation patterns of ART1 across a myriad of human cancers, uncovering its involvement in mutational events spanning multiple cancer types. Moreover, we turned our attention to RNA modification genes, recognizing their indispensable role in the initiation and progression of tumors. Through meticulous analysis, we elucidated a close interplay between ART1 and RNA modification genes, notably m1A, m5C, and m6A, across a broad spectrum of cancers. This underscores the pivotal role of ART1 in mediating tumorigenesis through the intricate regulation of RNA modification genes. Subsequently, our investigation delved into the correlations between ART1 expression and various clinicopathological characteristics, aiming to unravel the intricate associations between ART1 and the clinical manifestations of cancer. ART1's associations with key markers of tumor proliferation and growth processes, including DNAss, RNAss, EREG.EXPss, EREG-METHss, DMPss, and ENHss, underscore its intricate involvement in tumorigenesis and tumor progression. Given the pivotal role of the immune system in cancer development and treatment response, conducting a comprehensive pan-cancer analysis to elucidate the immunologic effects of ART1 becomes imperative for identifying cancer types that could potentially benefit from anti-ART1 immunotherapy. Our findings reveal a compelling correlation between ART1 expression and nearly all immune-related genes, with a particularly robust association observed with immune checkpoints. This underscores ART1 as a promising candidate for targeted tumor immunotherapy. The predictive value of ART1 in immunotherapy responses was consistently evident across multiple cohorts, wherein high expression levels of ART1 correlated with more favorable treatment responses. This emphasizes the pivotal role of ART1 in shaping the landscape of tumor immunotherapy. Recognizing the critical importance of immune cell infiltration in the TME, encompassing pivotal players such as CD4+ T cells, CD8+ T cells 41, 42, and macrophages, our results shed light on the potential mechanism by which ART1 modulates immune responses within the TME. Specifically, ART1-mediated acidification of P2X7R on CD8+ T cells appears to contribute to tumor immune resistance 43, 44, while P2X7R activation in antigen-presenting dendritic cells enhances neoplastic immunogenicity and amplifies the antitumor effects of anti-PD-1 antibodies 45. Moreover, the expression of ART1 in CD39 Treg cells may further contribute to resistance against cell death 46, 47. These compelling findings collectively underscore the intricate involvement of ART1 in modulating immune responses within the TME and position it as a promising target for enhancing the efficacy of immunotherapeutic interventions in cancer treatment.

Macrophages, known for their bidirectional regulatory effect on tumors, play a pivotal role in the intricate interplay within the TME 48. Our research unveils a positive correlation between ART1 expression and various immune cell types, including naïve B cells, CD8+ T cells, memory resting CD4+ T cells, resting NK cells, and macrophages. This intriguing association suggests that ART1 might exert influence over the immune landscape within the TME, positioning it as a potential target for novel tumor immunotherapeutic strategies. However, elucidating the precise mechanisms by which ART1 modulates the TME dynamics necessitates further in-depth investigation.

In the context of immune checkpoint inhibitors 36, 49, TMB and MSI emerge as critical biomarkers that hold prognostic significance. Elevated TMB and MSI levels have been correlated with improved survival outcomes, underlining their importance in guiding treatment decisions 29, 50.

Our findings underscore the substantial influence of ART1 on TMB and MSI, providing novel insights that could inform the development of more effective immunotherapeutic strategies. The intricate interplay observed between ART1 expression, immune cell types, and genomic features further accentuates the potential significance of ART1 as a central player in tumor immunology and a promising target for optimizing the outcomes of immunotherapy interventions. Undoubtedly, ART1 emerges as a significant contributor to tumorigenesis, yet the precise mechanistic underpinnings remain elusive. Functional enrichment analysis has shed light on potential pathways through which ART1 may exert its effects on tumorigenesis, implicating the NF-kB, Foxo, and PI3K-Akt signaling pathways. NF-kB, a pivotal nuclear transcription factor, governs genes involved in cell proliferation and apoptosis, and aberrations in NF-kB activation can perturb cellular homeostasis 51. Likewise, the PI3K/Akt and FoxO signaling cascades are recognized for their roles in mediating tumor cell proliferation and invasion during oncogenesis 52. Existing evidence suggests that downregulation of ART1 via the PI3K/Akt/NF-κB pathway promotes apoptosis in murine CT26 cells, indicating a potential mechanistic link between ART1 and apoptosis regulation 53. These initial insights into the molecular pathways influenced by ART1 underscore the imperative need for further exploration to unravel the intricate cellular processes modulated by ART1 within these signaling networks. Building upon these analyses, we conducted Bayesian co-localization analysis focusing on the relationship between ART1 and STAD. The results revealed a causal association between ART1 and STAD at the genetic level, further corroborating the potential role of ART1 in gastric cancer progression. Subsequently, to substantiate the cancer-promoting role of ART1 in gastric cancer, we performed experimental validation *in vitro*. Our results demonstrated elevated mRNA expressions of ART1 in MKN-45, AGS, and HGC-27 cell lines compared to normal GSE, accompanied by increased protein levels in the GC cell lines. Furthermore, the apoptosis level in HGC-27 cells was significantly lower in the OE-ART1 group compared to the control group, suggesting that ART1 overexpression inhibits apoptosis in GC cells. These experimental validations provide additional support for the notion that ART1 plays a pivotal role in promoting GC progression and may serve as a potential prognostic biomarker in GC.

This study marks the pioneering bioinformatic exploration of ART1 across pan-cancer, focusing on prognosis and the TME for the first time. The findings generated from this investigation bear significant translational implications for tumor diagnosis and therapeutic interventions. However, despite its contributions, the study is not without limitations. Firstly, the reliance on publicly available datasets imposes constraints associated with sample size limitations and inherent biases within the data sources. This limitation underscores the necessity for larger-scale studies and validation cohorts to corroborate our findings robustly. Secondly, while our bioinformatic analyses offer valuable insights into the potential roles of ART1 in tumor onset and progression, further *in vitro* experiments across diverse tumor types and clinical trials are imperative to elucidate the specific molecular mechanisms underlying ART1's involvement in cancer pathogenesis comprehensively. Thirdly, although our study has established correlations between ART1 expression, immune activity, and clinical survival across various cancer types, definitive confirmation of whether ART1 directly impacts clinical outcomes through immune-mediated pathways requires additional experimental validation. Lastly, the intricate interplay between ART1 and the tumor microenvironment necessitates further exploration through rigorous experimental studies. Future research endeavors will focus on elucidating the intricate molecular mechanisms underlying ART1's interactions within the TME to unveil its full therapeutic potential in cancer management.

In summary, our study sheds light on the intricate relationship between ART1 expression levels, tumor prognosis, and the extent of immune infiltration across a spectrum of cancers. These findings underscore the potential utility of ART1 as a biomarker for identifying targets in tumor immunotherapy. Moreover, our results highlight the role of ART1 in promoting GC growth, emphasizing the importance of further investigation into the interplay between ART1 and the TIME. This prospective inquiry holds substantial promise, laying a novel foundation for advancing immunotherapeutic strategies in cancer treatment.

## 5. Conclusion

This study marks the first comprehensive exploration of ART1's involvement across diverse cancer types. Our findings revealed distinct expression patterns of ART1 between tumor and normal tissues, highlighting its close correlation with clinical prognosis, immune-related genes, RNA modification genes, tumor stemness, and clinical phenotypes. Additionally, *in vitro* experiments provided validation of ART1's oncogenic role in GC.

## Supplementary Material

Supplementary figures and tables.

## Abbreviations

irAE: immune-related adverse event
ART1: Mono-ADP-ribosyltransferases-1
ICI: immune checkpoint inhibitors
ACC: Adrenocortical carcinoma
BLCA: Bladder Urothelial Carcinoma
BRCA: Breast invasive carcinoma
CESC: Cervical squamous cell carcinoma and endocervical adenocarcinoma
CHOL: Cholangiocarcinoma
COAD: Colon adenocarcinoma
COADREAD: Colon adenocarcinoma/Rectum adenocarcinoma Esophageal carcinoma
DLBC: Lymphoid Neoplasm Diffuse Large B-cell Lymphoma
ESCA: Esophageal carcinoma
GBM: Glioblastoma multiforme
GBMLGG: Glioma
HNSC: Head and Neck squamous cell carcinoma
KICH: Kidney Chromophobe
KIPAN: Pan-kidney cohort
KIRC: Kidney renal clear cell carcinoma
KIRP: Kidney renal papillary cell carcinoma
LAML: Acute Myeloid Leukemia
LGG: Brain Lower Grade Glioma
LIHC: Liver hepatocellular carcinoma
LUAD: Lung adenocarcinoma
LUSC: Lung squamous cell carcinoma
MESO: Mesothelioma
OV: Ovarian serous cystadenocarcinoma
OSCC: Oral squamous cell carcinoma
PAAD: Pancreatic adenocarcinoma
PCPG: Pheochromocytoma and Paraganglioma
PRAD: Prostate adenocarcinoma
READ: Rectum adenocarcinoma
SARC: Sarcoma
SKCM: Skin Cutaneous Melanoma
STAD: Stomach adenocarcinoma
STES: Stomach and Esophageal carcinoma
TGCT: Testicular Germ Cell Tumors
THCA: Thyroid carcinoma
THYM: Thymoma
UCEC: Uterine Corpus Endometrial Carcinoma
UCS: Uterine Carcinosarcoma
UVM: Uveal Melanoma
OS: Osteosarcoma
ALL: Acute Lymphoblastic Leukemia
NB: Neuroblastoma
WT: High-Risk Wilms Tumor
TMB: Tumor mutation burden
TME: tumor microenvironment
MSI: Microsatellite instability
MATH: Mutant-allele tumor heterogeneity
BP: Biological process
CC: Cellular component
MF: Molecular function
FDR: Error Detection Rate
KEGG: Kyoto Encyclopedia of Genes and Genomes
PPI: Protein-protein interaction
