# Supplementary material for: Pan-Cancer Analysis of ART1 and its Potential Value in Gastric Cancer
Source: J Cancer. 2024 May 13;15(12):3684–707. doi: 10.7150/jca.96033 (PMC11190775; doi:10.7150/jca.96033)

Supplementary Figure 1 Correlation between ART1 and Survival analysis

A Forest map associated with OS in pan cancers

CancerCode	pvalue	Hazard Ratio(95%CI)
TCGA-DLBC(N=44)	9.6e-3	1.43(1.06,1.94)
TCGA-LAML(N=209)	0.02	1.07(1.01,1.13)
TCGA-SKCM-P(N=97)	0.04	1.12(1.00,1.25)
TCGA-OV(N=407)	0.16	1.04(0.99,1.09)
TCGA-STAD(N=372)	0.20	1.04(0.98,1.09)
TCGA-SKCM(N=444)	0.21	1.03(0.98,1.09)
TCGA-STES(N=547)	0.25	1.03(0.98,1.07)
TCGA-THYM(N=117)	0.30	1.15(0.88,1.50)
TCGA-LUSC(N=468)	0.38	1.02(0.97,1.08)
TCGA-LIHC(N=341)	0.38	1.04(0.95,1.15)
TCGA-HNSC(N=509)	0.42	1.02(0.98,1.06)
TCGA-ACC(N=77)	0.42	1.10(0.88,1.37)
TCGA-SKCM-M(N=347)	0.44	1.02(0.97,1.08)
TARGET-NB(N=151)	0.47	1.03(0.95,1.12)
TCGA-THCA(N=501)	0.58	1.05(0.88,1.25)
TARGET-ALL-R(N=99)	0.72	1.02(0.90,1.17)
TCGA-ESCA(N=175)	0.78	1.01(0.93,1.10)
TCGA-CHOL(N=33)	0.81	1.02(0.84,1.24)
TCGA-BRCA(N=1044)	0.91	1.00(0.95,1.06)
TCGA-PRAD(N=492)	0.96	1.01(0.82,1.24)
TCGA-GBMLGG(N=619)	1.3e-11	0.87(0.84,0.91)
TCGA-KICH(N=64)	5.0e-3	0.55(0.32,0.94)
TCGA-BLCA(N=398)	0.02	0.90(0.81,0.99)
TCGA-UCS(N=55)	0.08	0.91(0.81,1.01)
TCGA-KIRC(N=515)	0.09	0.96(0.92,1.01)
TARGET-WT(N=80)	0.16	0.89(0.76,1.05)
TCGA-PCPG(N=170)	0.16	0.78(0.55,1.13)
TCGA-CESC(N=273)	0.20	0.93(0.82,1.04)
TARGET-LAML(N=142)	0.28	0.90(0.74,1.09)
TCGA-TGCT(N=128)	0.40	0.01(0.0e+0,NaN)
TCGA-KIPAN(N=855)	0.51	0.99(0.95,1.03)
TCGA-UVM(N=74)	0.66	0.97(0.84,1.12)
TCGA-UCEC(N=166)	0.71	0.97(0.85,1.12)
TCGA-COAD(N=278)	0.73	0.98(0.89,1.08)
TARGET-ALL(N=86)	0.75	0.98(0.85,1.13)
TCGA-COADREAD(N=368)	0.76	0.99(0.91,1.08)
TCGA-SARC(N=254)	0.81	0.99(0.92,1.07)
TCGA-KIRP(N=276)	0.84	0.99(0.87,1.12)
TCGA-PAAD(N=172)	0.85	0.99(0.93,1.07)
TCGA-LGG(N=474)	0.86	0.99(0.93,1.06)
TCGA-GBM(N=144)	0.88	0.99(0.93,1.07)
TCGA-MESO(N=84)	0.88	0.99(0.89,1.11)
TCGA-READ(N=90)	0.93	0.99(0.82,1.20)
TCGA-LUAD(N=490)	0.98	1.00(0.95,1.05)

log2(Hazard Ratio(95%CI))

B Forest map associated with DSS in pan cancers

CancerCode	pvalue	Hazard Ratio(95%CI)
TCGA-OV(N=378)	0.06	1.05(1.00,1.11)
TCGA-SKCM-P(N=97)	0.12	1.11(0.97,1.26)
TCGA-SKCM(N=438)	0.31	1.03(0.97,1.09)
TCGA-DLBC(N=44)	0.32	1.26(0.78,2.04)
TCGA-STAD(N=351)	0.34	1.03(0.97,1.11)
TCGA-HNSC(N=485)	0.36	1.02(0.97,1.08)
TCGA-STES(N=524)	0.39	1.03(0.97,1.08)
TCGA-SKCM-M(N=341)	0.50	1.02(0.96,1.08)
TCGA-LUAD(N=457)	0.54	1.02(0.96,1.08)
TCGA-ACC(N=75)	0.58	1.08(0.83,1.40)
TCGA-READ(N=84)	0.63	1.08(0.80,1.45)
TCGA-CHOL(N=32)	0.70	1.04(0.85,1.26)
TCGA-ESCA(N=173)	0.74	1.02(0.92,1.12)
TCGA-PAAD(N=166)	0.91	1.00(0.93,1.09)
TCGA-COADREAD(N=347)	0.97	1.00(0.89,1.13)
TCGA-GBMLGG(N=598)	2.5e-10	0.87(0.84,0.91)
TCGA-KIRC(N=504)	0.02	0.94(0.88,0.99)
TCGA-BLCA(N=385)	0.02	0.86(0.76,0.98)
TCGA-KICH(N=64)	0.02	0.58(0.34,1.01)
TCGA-KIPAN(N=840)	0.11	0.96(0.91,1.01)
TCGA-UCS(N=53)	0.16	0.92(0.81,1.04)
TCGA-THCA(N=495)	0.19	0.85(0.66,1.09)
TCGA-THYM(N=117)	0.27	6.3e-3(0.0e+0,NaN)
TCGA-KIRP(N=272)	0.37	0.92(0.77,1.10)
TCGA-BRCA(N=1025)	0.39	0.97(0.90,1.04)
TCGA-TGCT(N=128)	0.40	0.01(0.0e+0,NaN)
TCGA-UCEC(N=164)	0.44	0.93(0.78,1.11)
TCGA-PCPG(N=170)	0.46	0.87(0.59,1.28)
TCGA-LUSC(N=418)	0.57	0.98(0.90,1.06)
TCGA-CESC(N=269)	0.58	0.96(0.85,1.09)
TCGA-UVM(N=74)	0.62	0.96(0.83,1.12)
TCGA-PRAD(N=490)	0.67	0.93(0.68,1.28)
TCGA-SARC(N=248)	0.73	0.98(0.90,1.07)
TCGA-LIHC(N=333)	0.75	0.98(0.85,1.12)
TCGA-COAD(N=263)	0.82	0.99(0.87,1.12)
TCGA-GBM(N=131)	0.87	0.99(0.92,1.07)
TCGA-LGG(N=466)	0.92	1.00(0.93,1.07)
TCGA-MESO(N=64)	0.92	0.99(0.86,1.14)

log2(Hazard Ratio(95%CI))

C Forest map associated with DFI in pan cancers

CancerCode	pvalue	Hazard Ratio(95%CI)
TCGA-CESC(N=171)	0.01	1.19(1.03,1.37)
TCGA-HNSC(N=128)	0.05	1.11(1.00,1.23)
TCGA-STAD(N=232)	0.16	1.08(0.97,1.19)
TCGA-COAD(N=103)	0.25	1.10(0.93,1.30)
TCGA-PAAD(N=68)	0.32	1.07(0.93,1.23)
TCGA-KIRP(N=177)	0.38	1.08(0.91,1.28)
TCGA-COADREAD(N=132)	0.44	1.06(0.91,1.24)
TCGA-OV(N=203)	0.50	1.02(0.95,1.10)
TCGA-BLCA(N=184)	0.56	1.05(0.89,1.24)
TCGA-SARC(N=149)	0.70	1.02(0.93,1.11)
TCGA-STES(N=316)	0.77	1.01(0.93,1.10)
TCGA-PRAD(N=337)	0.80	1.01(0.91,1.14)
TCGA-ACC(N=44)	0.89	1.03(0.66,1.62)
TCGA-LUAD(N=295)	0.98	1.00(0.94,1.07)
TCGA-THCA(N=352)	0.03	0.87(0.76,0.99)
TCGA-LGG(N=126)	0.15	0.91(0.79,1.04)
TCGA-GBMLGG(N=127)	0.17	0.91(0.80,1.04)
TCGA-KIPAN(N=319)	0.21	0.94(0.85,1.04)
TCGA-UCS(N=26)	0.21	0.88(0.72,1.09)
TCGA-LIHC(N=294)	0.24	0.94(0.84,1.04)
TCGA-TGCT(N=101)	0.25	0.89(0.73,1.09)
TCGA-PCPG(N=152)	0.27	0.79(0.51,1.24)
TCGA-BRCA(N=904)	0.30	0.96(0.90,1.03)
TCGA-ESCA(N=84)	0.32	0.91(0.76,1.10)
TCGA-MESO(N=14)	0.32	0.81(0.53,1.25)
TCGA-CHOL(N=23)	0.35	0.85(0.59,1.21)
TCGA-KIRC(N=113)	0.38	0.94(0.81,1.08)
TCGA-KICH(N=29)	0.41	0.81(0.48,1.36)
TCGA-DLBC(N=26)	0.46	0.01(0.0e+0,NaN)
TCGA-READ(N=29)	0.47	0.85(0.54,1.34)
TCGA-LUSC(N=292)	0.72	0.98(0.89,1.08)
TCGA-UCEC(N=115)	0.94	0.99(0.81,1.22)

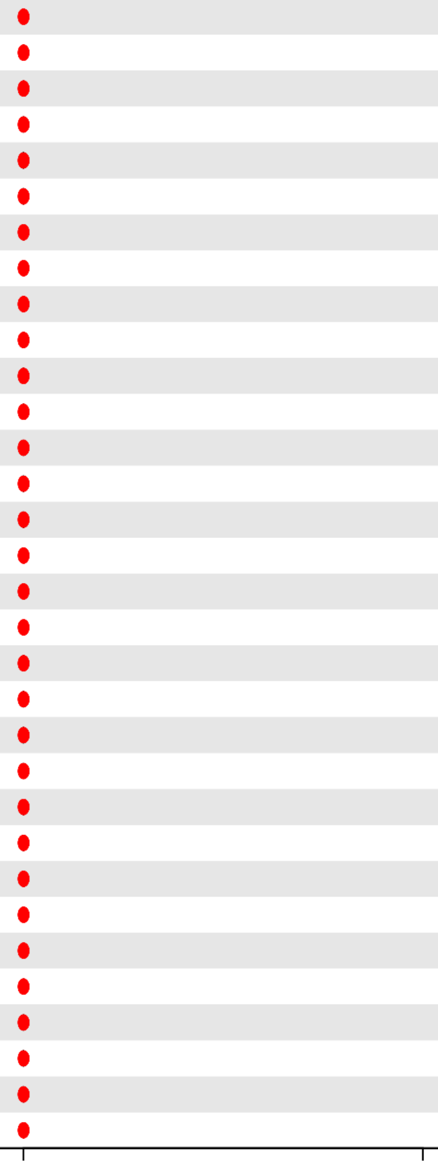

D Forest map associated with PFI in pan cancers

CancerCode	pvalue	Hazard Ratio(95%CI)
TCGA-SKCM-P(N=96)	4.7e-3	1.17(1.04,1.31)
TCGA-STAD(N=375)	0.10	1.05(0.99,1.11)
TCGA-GBM(N=143)	0.15	1.05(0.98,1.12)
TCGA-CESC(N=273)	0.19	1.07(0.97,1.17)
TCGA-OV(N=407)	0.19	1.03(0.98,1.08)
TCGA-HNSC(N=508)	0.28	1.02(0.98,1.07)
TCGA-DLBC(N=43)	0.41	1.16(0.82,1.63)
TCGA-STES(N=548)	0.46	1.02(0.97,1.06)
TCGA-LGG(N=472)	0.53	1.02(0.97,1.07)
TCGA-PRAD(N=492)	0.56	1.02(0.96,1.09)
TCGA-ACC(N=76)	0.64	1.05(0.86,1.27)
TCGA-UVM(N=73)	0.66	1.03(0.90,1.19)
TCGA-SKCM(N=434)	0.74	1.01(0.96,1.06)
TCGA-UCEC(N=166)	0.81	1.01(0.90,1.14)
TCGA-LUAD(N=486)	0.86	1.00(0.96,1.05)
TCGA-LUSC(N=467)	0.94	1.00(0.94,1.07)
TCGA-CHOL(N=33)	0.97	1.00(0.83,1.22)
TCGA-GBMLGG(N=616)	2.7e-5	0.93(0.89,0.96)
TCGA-KIRC(N=508)	5.5e-3	0.93(0.89,0.98)
TCGA-KICH(N=64)	9.0e-3	0.66(0.46,0.94)
TCGA-THCA(N=499)	0.01	0.89(0.81,0.98)
TCGA-KIPAN(N=845)	0.05	0.96(0.92,1.00)
TCGA-UCS(N=55)	0.11	0.92(0.83,1.02)
TCGA-PCPG(N=168)	0.13	0.88(0.74,1.04)
TCGA-BLCA(N=397)	0.24	0.95(0.87,1.04)
TCGA-LIHC(N=340)	0.31	0.95(0.87,1.05)
TCGA-THYM(N=117)	0.38	0.91(0.73,1.13)
TCGA-READ(N=88)	0.46	0.93(0.76,1.13)
TCGA-SARC(N=250)	0.47	0.98(0.91,1.04)
TCGA-COADREAD(N=363)	0.47	0.97(0.90,1.05)
TCGA-TGCT(N=126)	0.51	0.95(0.80,1.11)
TCGA-MESO(N=82)	0.57	0.96(0.84,1.10)
TCGA-COAD(N=275)	0.60	0.98(0.90,1.07)
TCGA-SKCM-M(N=338)	0.70	0.99(0.94,1.04)
TCGA-BRCA(N=1043)	0.72	0.99(0.94,1.04)
TCGA-ESCA(N=173)	0.78	0.99(0.92,1.07)
TCGA-KIRP(N=273)	0.87	0.99(0.88,1.11)
TCGA-PAAD(N=171)	0.93	1.00(0.93,1.07)

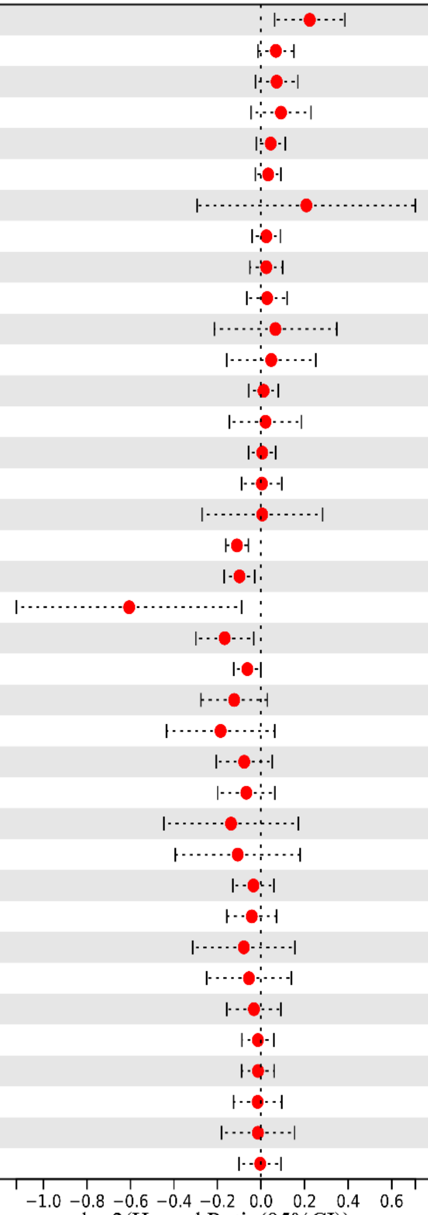

Supplement: Supplementary file 1 — Supplementary figures and tables. [file jcav15p3684s1.zip › Supplementary files/Supplementary Figure 1 Correlation between ART1 and Survival analysis.pdf]
