# Supplementary material for: Pan-Cancer Analysis of ART1 and its Potential Value in Gastric Cancer
Source: J Cancer. 2024 May 13;15(12):3684–707. doi: 10.7150/jca.96033 (PMC11190775; doi:10.7150/jca.96033)
Supplement: Supplementary file 1 — Supplementary figures and tables. [file jcav15p3684s1.zip › Supplementary files/Supplementary Figure 3 Immune Score of ART1 calculated by ESTIMATE Algorithm in pan cancers.pdf]

Supplementary Figure 3 Immune Score of ART1 calculated by ESTIMATE Algorithm in pan cancers

A Estimate Score of ART1 in pan cancers

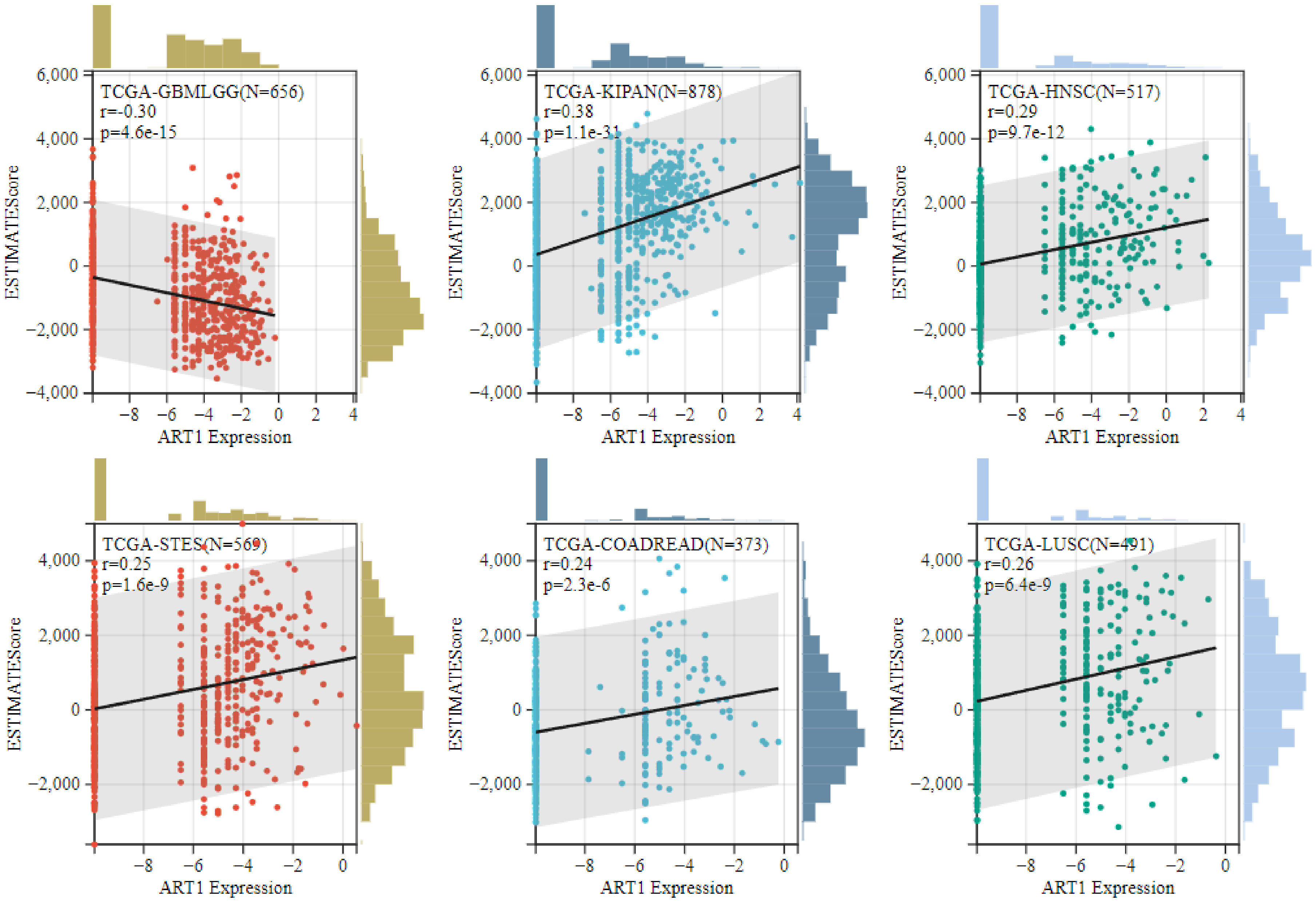

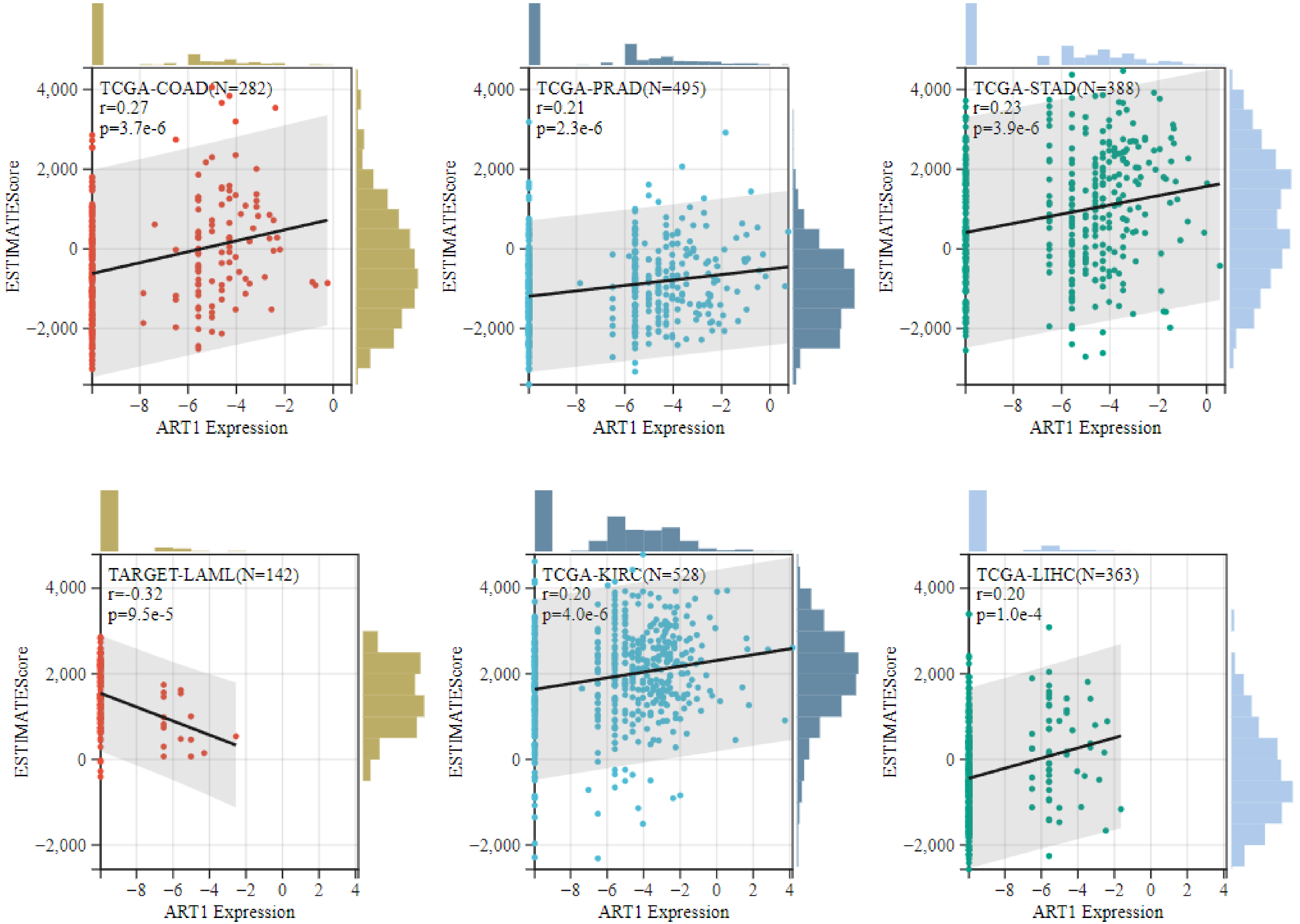

TCGA-COAD(N=282)
r=0.27
p=3.7e-6
TCGA-PRAD(N=495)
r=0.21
p=2.3e-6
TCGA-STAD(N=388)
r=0.23
p=3.9e-6
TARGET-LAML(N=142)
r=-0.32
p=9.5e-5
TCGA-KIRC(N=528)
r=0.20
p=4.0e-6
TCGA-LIHC(N=363)
r=0.20
p=1.0e-4
ESTIMATEScore
ART1 Expression

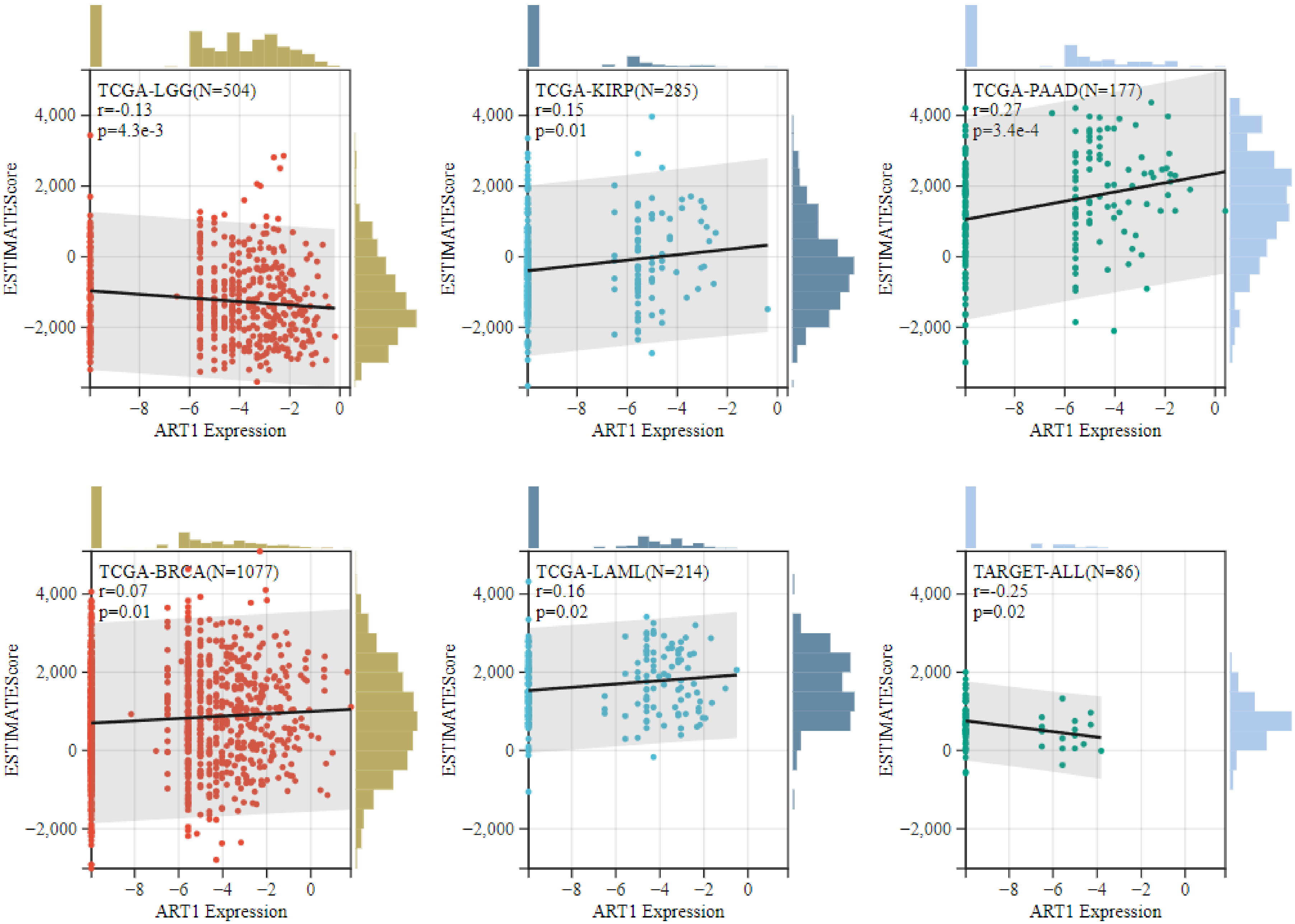

TCGA-LGG(N=504)
r=-0.13
p=4.3e-3
TCGA-KIRP(N=285)
r=0.15
p=0.01
TCGA-PAAD(N=177)
r=0.27
p=3.4e-4
TCGA-BRCA(N=1077)
r=0.07
p=0.01
TCGA-LAML(N=214)
r=0.16
p=0.02
TARGET-ALL(N=86)
r=-0.25
p=0.02
ESTIMATEScore
ART1 Expression

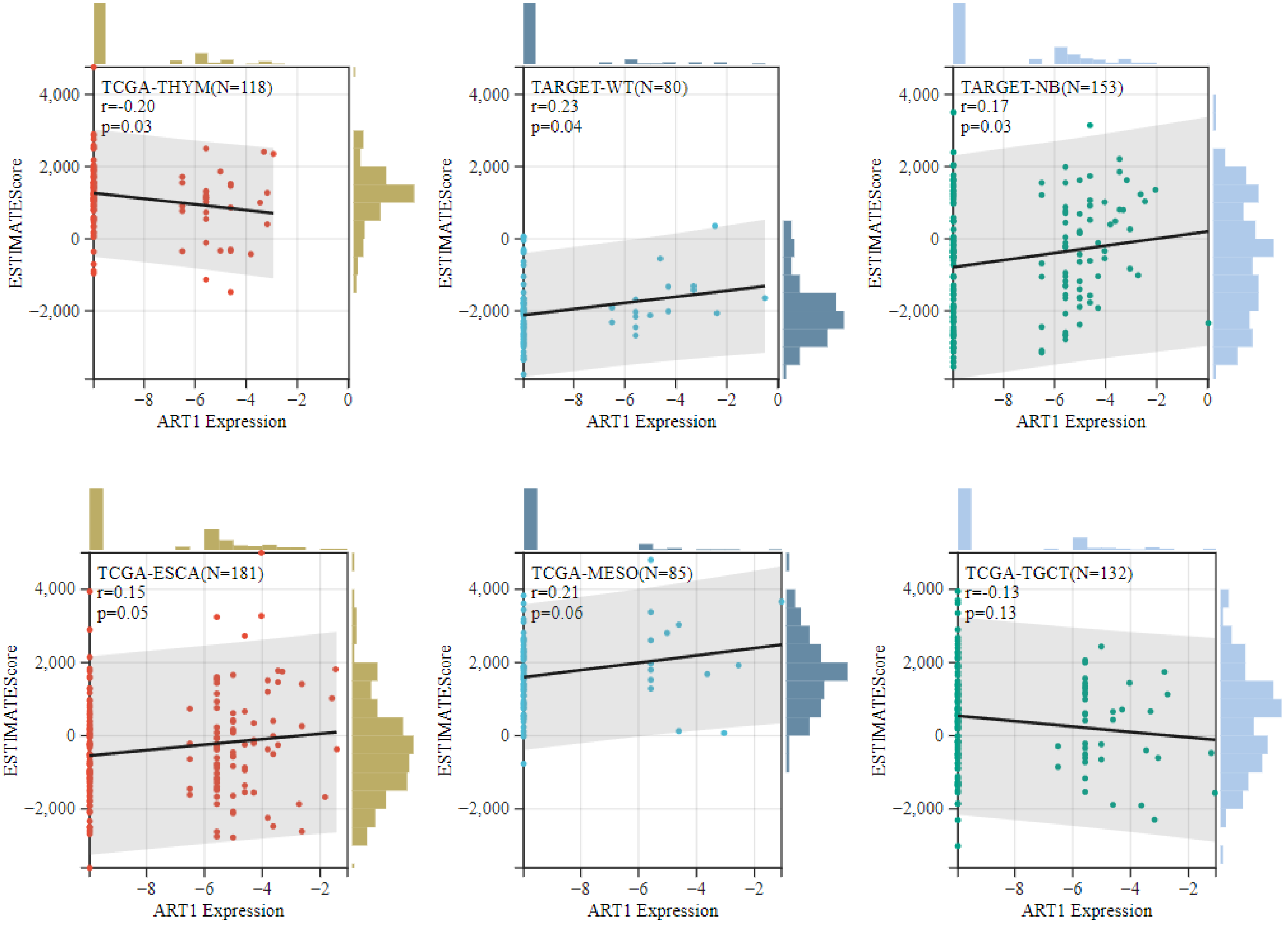
TCGA-THYM(N=118)
r=-0.20
p=0.03
TARGET-WT(N=80)
r=0.23
p=0.04
TARGET-NB(N=153)
r=0.17
p=0.03
TCGA-ESCA(N=181)
r=0.15
p=0.05
TCGA-MESO(N=85)
r=0.21
p=0.06
TCGA-TGCT(N=132)
r=-0.13
p=0.13
ESTIMATEScore
ART1 Expression

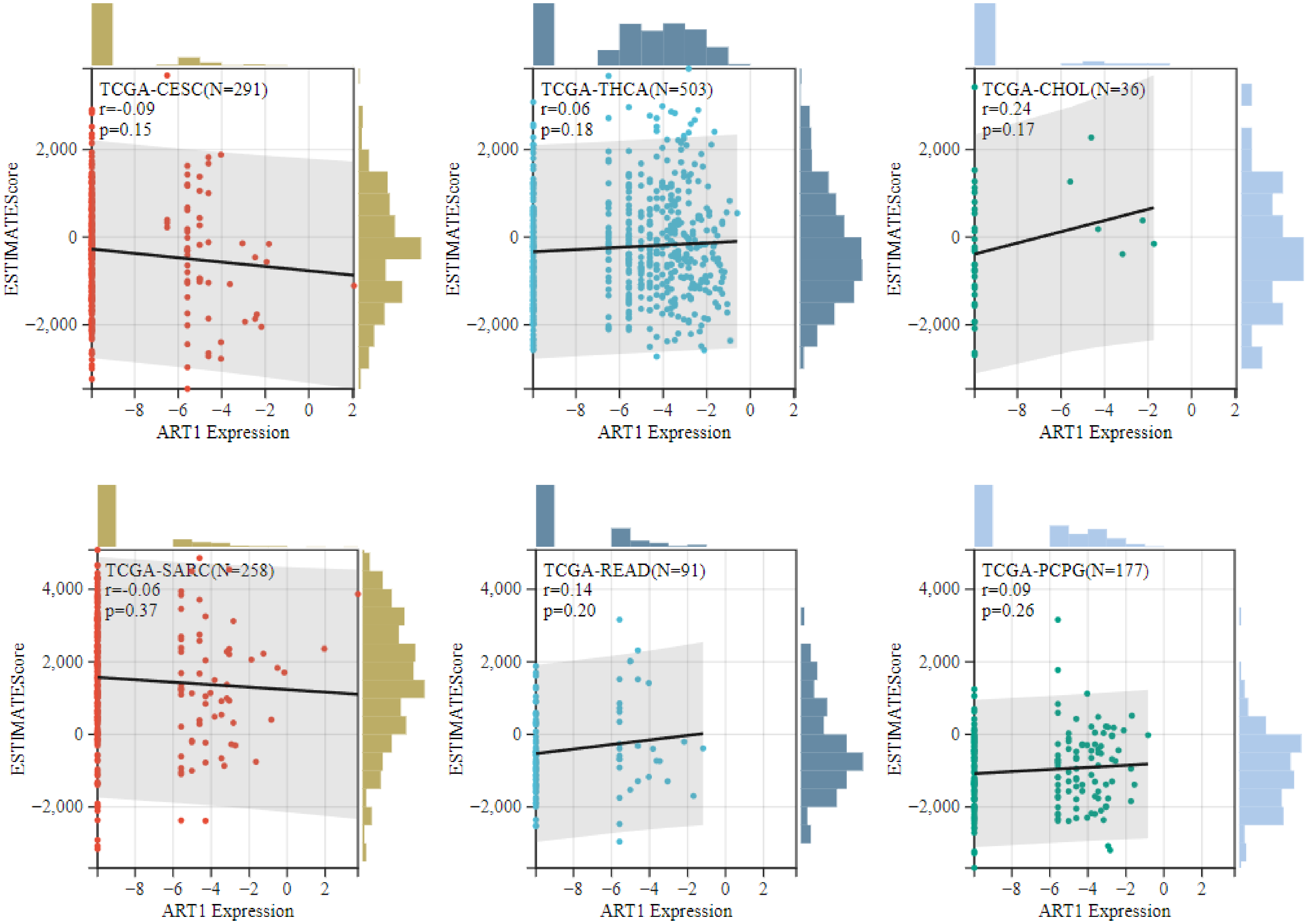

TCGA-CESC(N=291)
r=-0.09
p=0.15
TCGA-THCA(N=503)
r=0.06
p=0.18
TCGA-CHOL(N=36)
r=0.24
p=0.17
TCGA-SARC(N=258)
r=-0.06
p=0.37
TCGA-READ(N=91)
r=0.14
p=0.20
TCGA-PCPG(N=177)
r=0.09
p=0.26
ESTIMATEScore
ART1 Expression

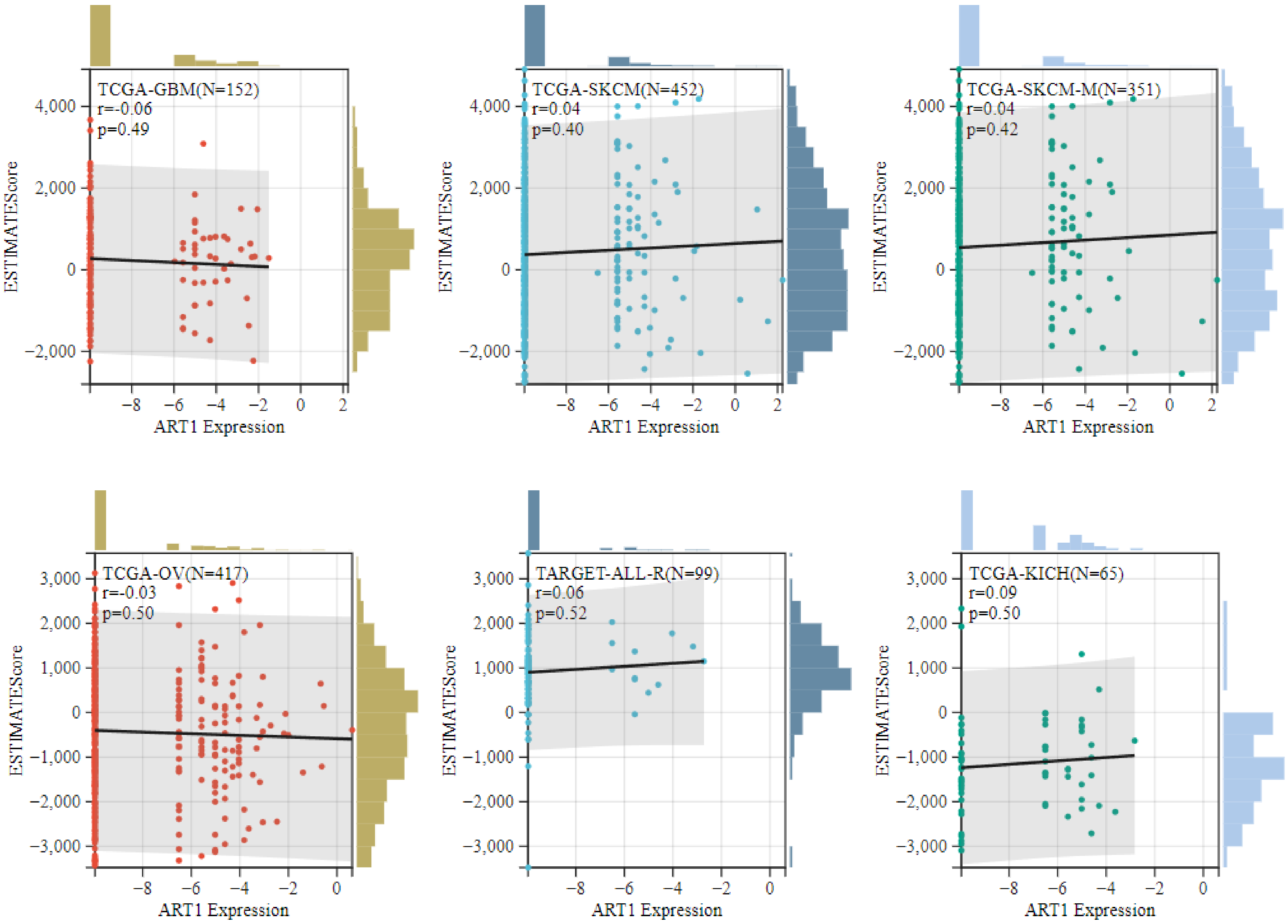
TCGA-GBM(N=152)
r=-0.06
p=0.49
TCGA-SKCM(N=452)
r=0.04
p=0.40
TCGA-SKCM-M(N=351)
r=0.04
p=0.42
TCGA-OV(N=417)
r=-0.03
p=0.50
TARGET-ALL-R(N=99)
r=0.06
p=0.52
TCGA-KICH(N=65)
r=0.09
p=0.50
ESTIMATEScore
ART1 Expression

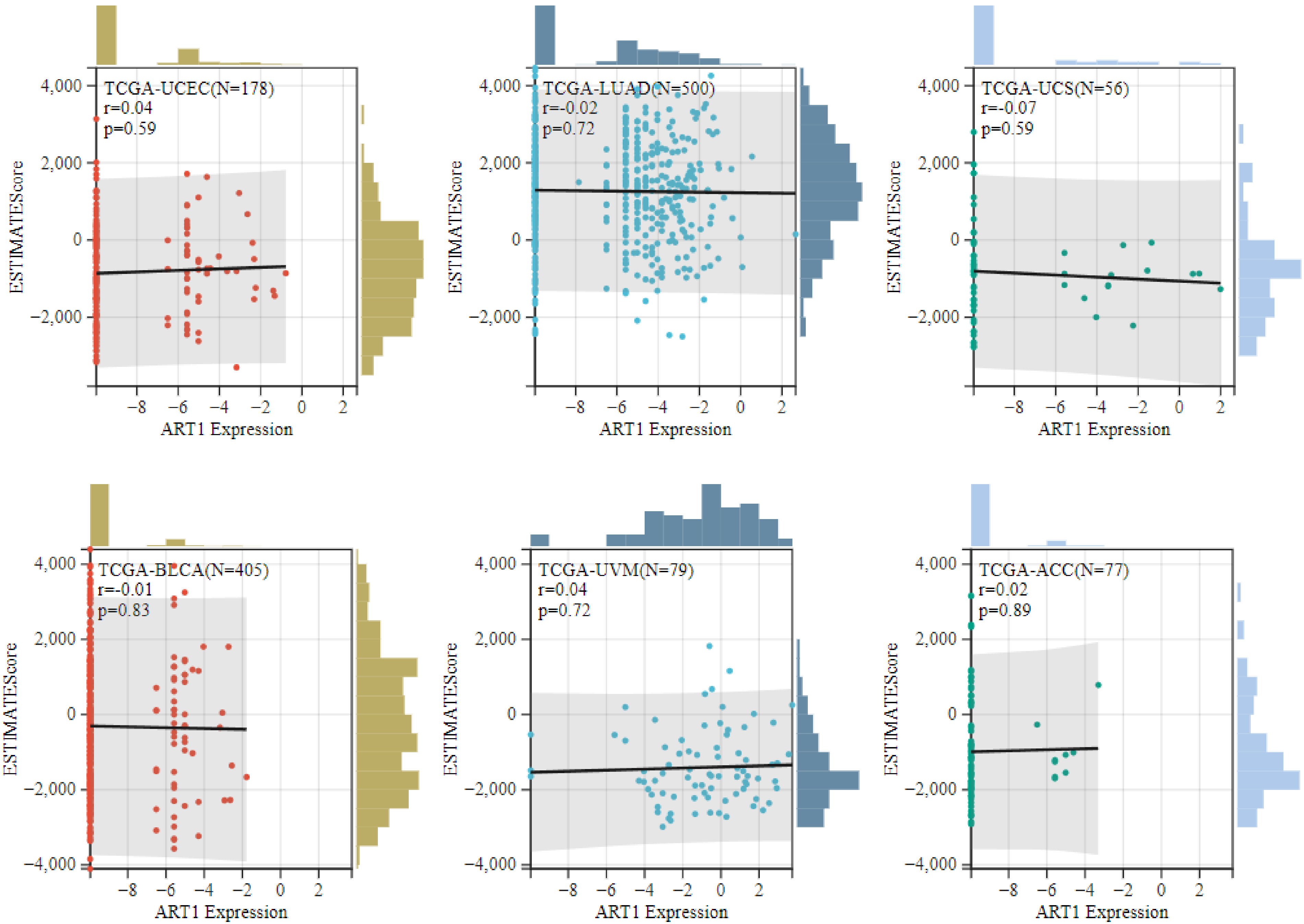

TCGA-UCEC(N=178)
r=0.04
p=0.59
TCGA-LUAD(N=500)
r=-0.02
p=0.72
TCGA-UCS(N=56)
r=-0.07
p=0.59
TCGA-BLCA(N=405)
r=-0.01
p=0.83
TCGA-UVM(N=79)
r=0.04
p=0.72
TCGA-ACC(N=77)
r=0.02
p=0.89
ESTIMATEScore
ART1 Expression

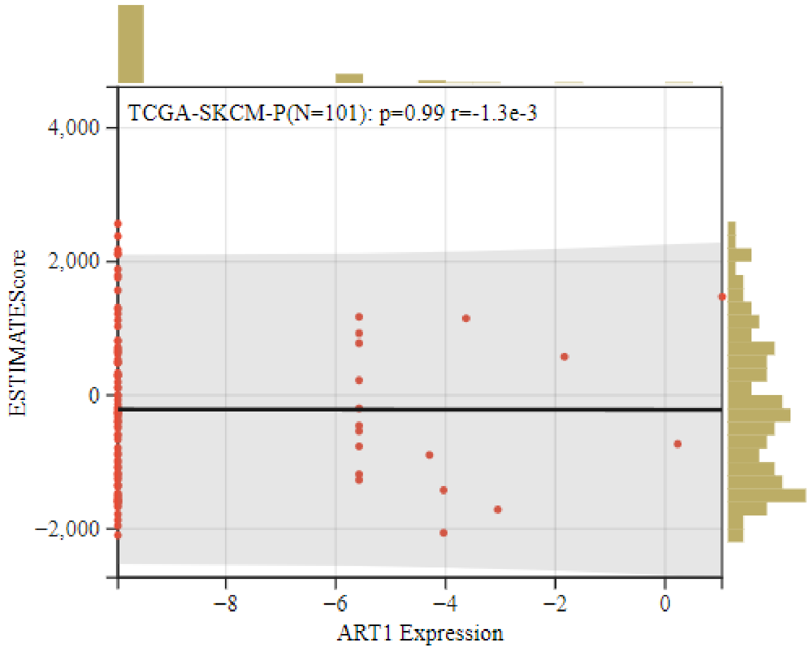

TCGA-SKCM-P(N=101): p=0.99 r=-1.3e-3
ESTIMATEScore
4,000
2,000
0
−2,000
−8
−6
−4
−2
0
ART1 Expression

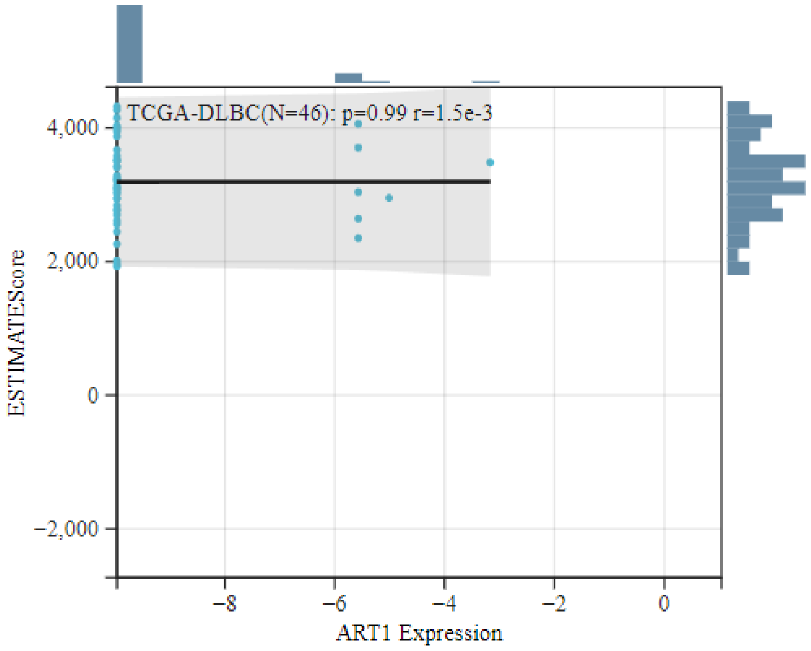

TCGA-DLBC(N=46): p=0.99 r=1.5e-3
ESTIMATEScore
4,000
2,000
0
−2,000
−8
−6
−4
−2
0
ART1 Expression

B Immune Score of ART1 in pan cancers

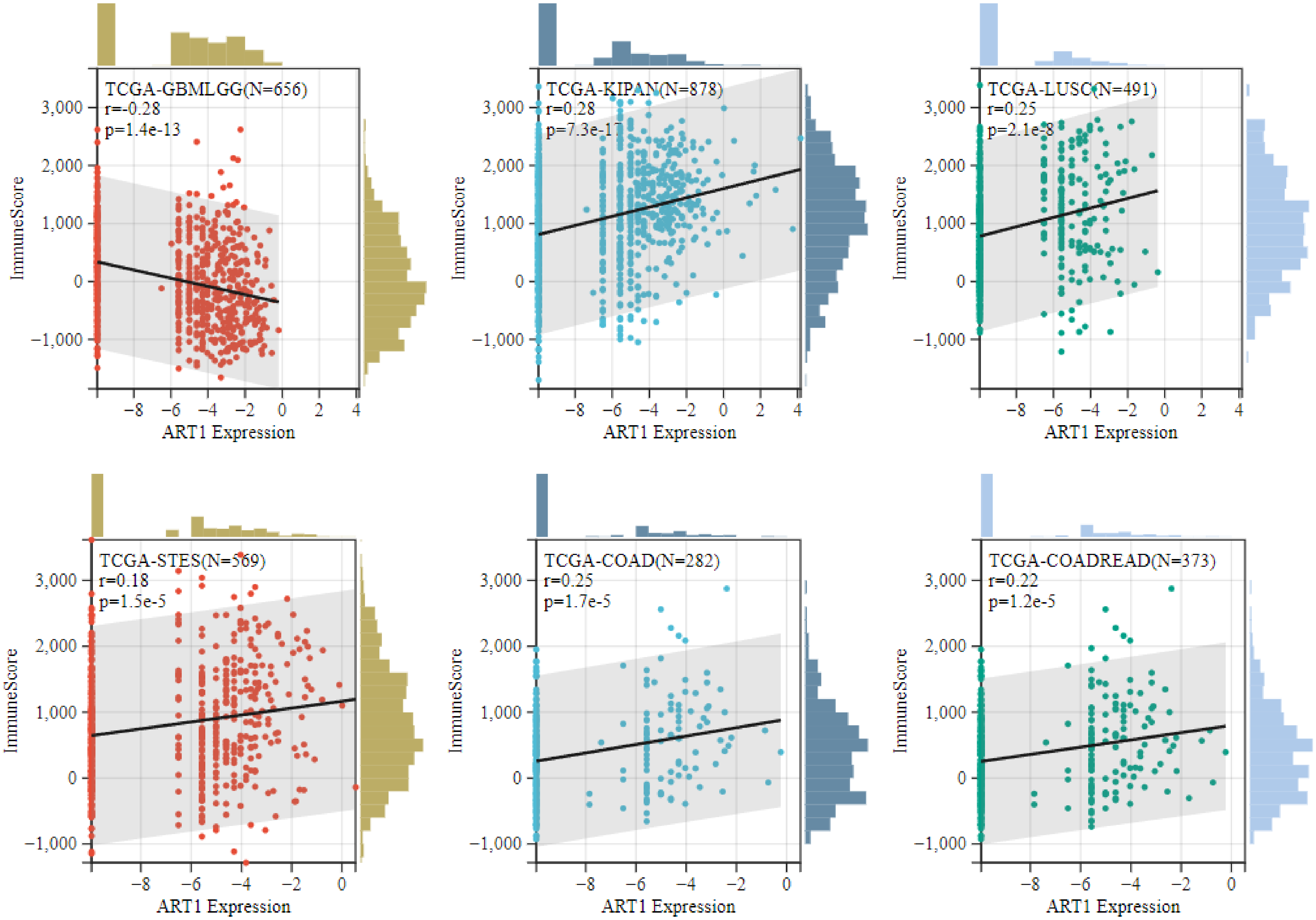

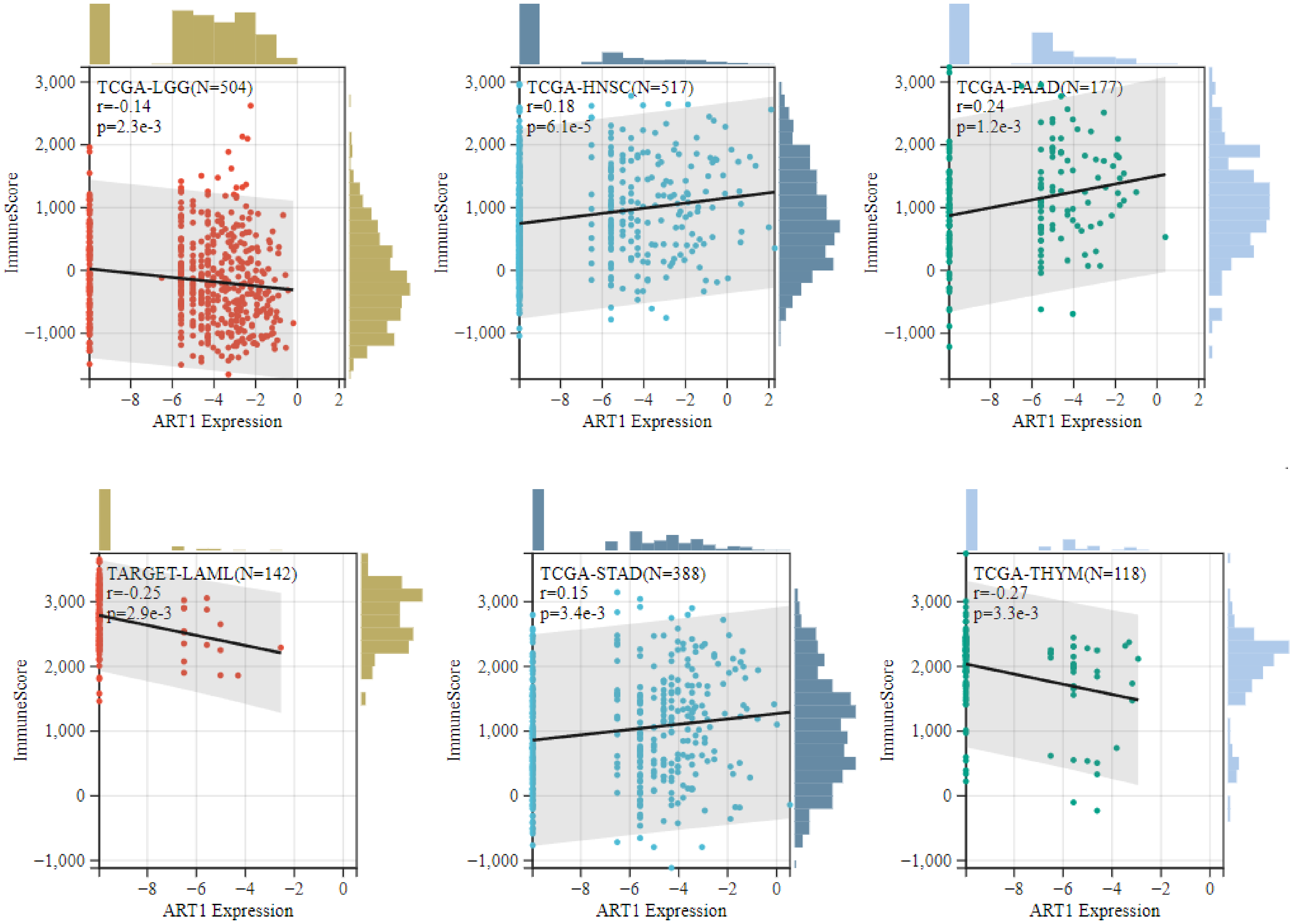
TCGA-LGG(N=504)
r=-0.14
p=2.3e-3
TCGA-HNSC(N=517)
r=0.18
p=6.1e-5
TCGA-PAAD(N=177)
r=0.24
p=1.2e-3
TARGET-LAML(N=142)
r=-0.25
p=2.9e-3
TCGA-STAD(N=388)
r=0.15
p=3.4e-3
TCGA-THYM(N=118)
r=-0.27
p=3.3e-3
ImmuneScore
ART1 Expression

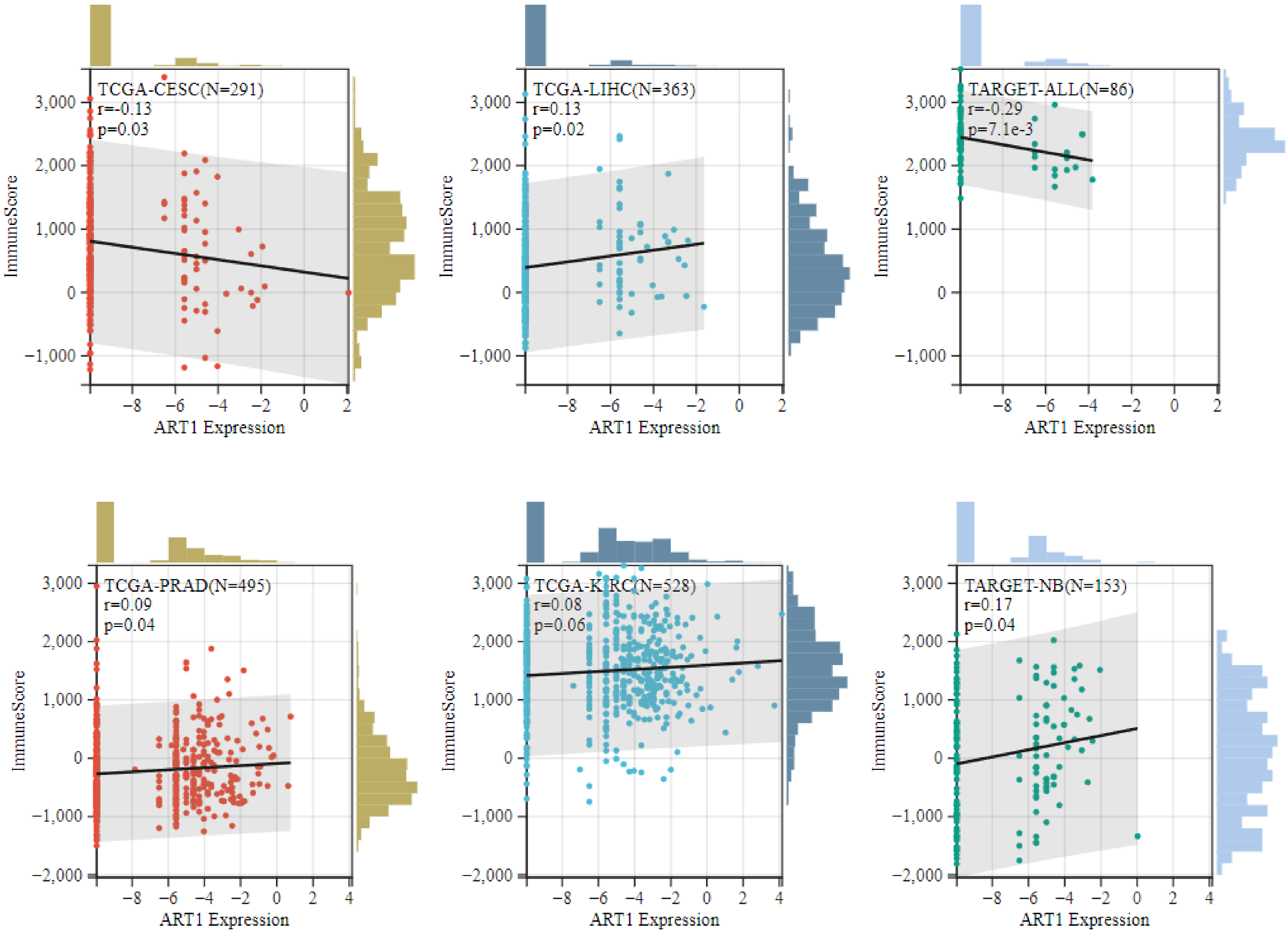

TCGA-CESC(N=291)
r=-0.13
p=0.03
TCGA-LIHC(N=363)
r=0.13
p=0.02
TARGET-ALL(N=86)
r=-0.29
p=7.1e-3
TCGA-PRAD(N=495)
r=0.09
p=0.04
TCGA-K RC(N=528)
r=0.08
p=0.06
TARGET-NB(N=153)
r=0.17
p=0.04
ImmuneScore
ART1 Expression

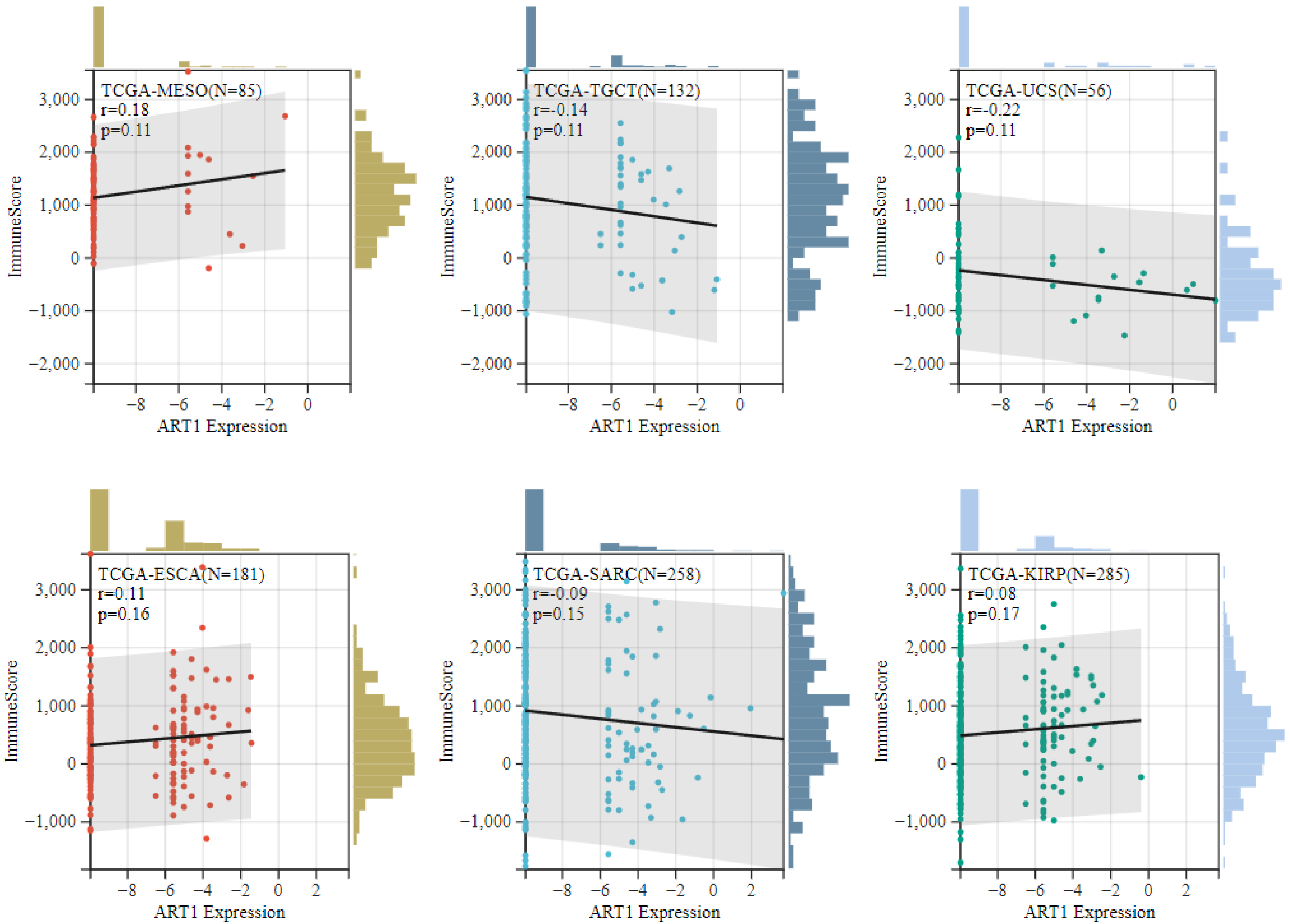

TCGA-MESO(N=85)
r=0.18
p=0.11
TCGA-TGCT(N=132)
r=-0.14
p=0.11
TCGA-UCS(N=56)
r=-0.22
p=0.11
TCGA-ESCA(N=181)
r=0.11
p=0.16
TCGA-SARC(N=258)
r=-0.09
p=0.15
TCGA-KIRP(N=285)
r=0.08
p=0.17
ImmuneScore
ART1 Expression

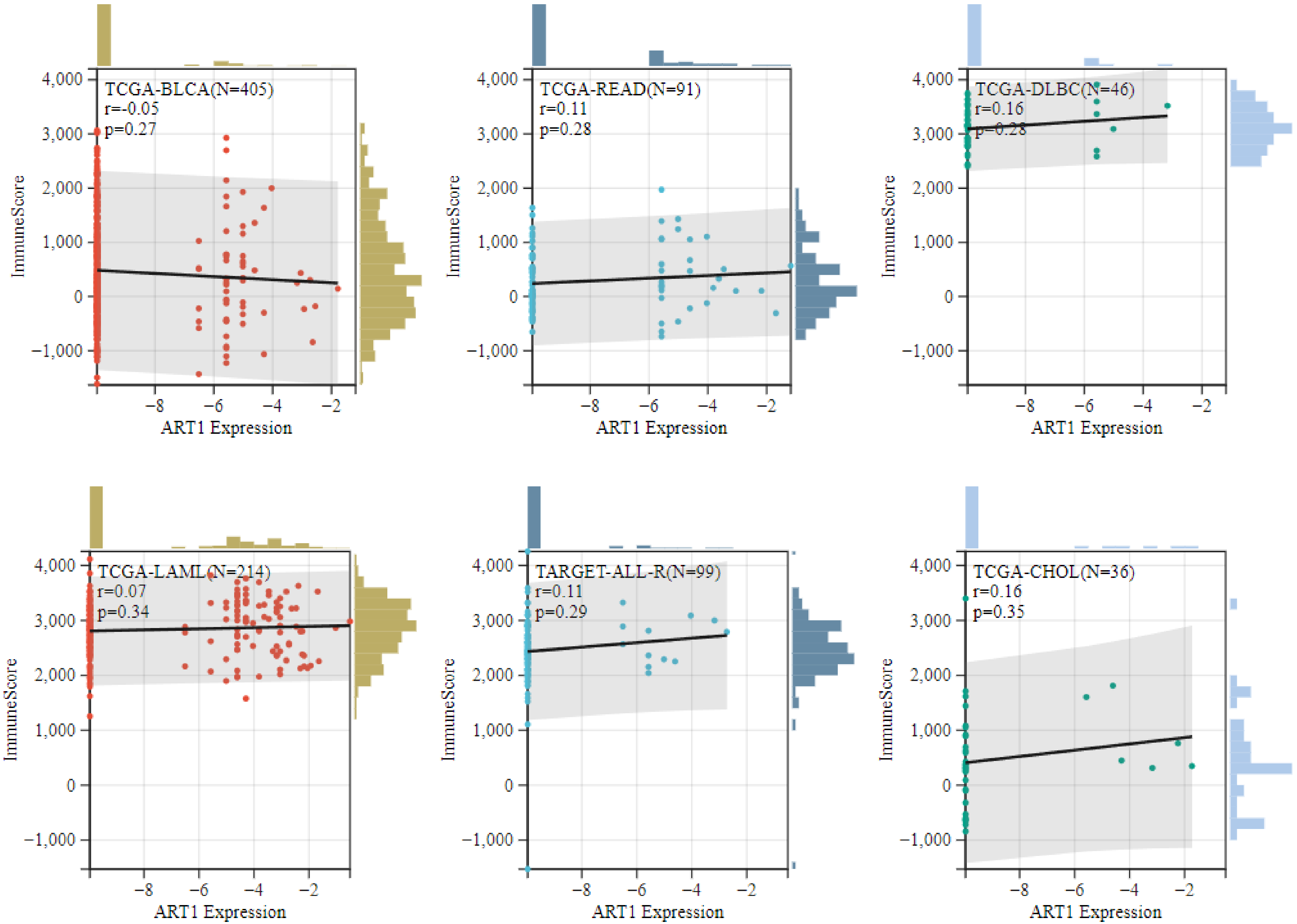
TCGA-BLCA(N=405)
r=-0.05
p=0.27
TCGA-READ(N=91)
r=0.11
p=0.28
TCGA-DLBC(N=46)
r=0.16
p=0.28
TCGA-LAML(N=214)
r=0.07
p=0.34
TARGET-ALL-R(N=99)
r=0.11
p=0.29
TCGA-CHOL(N=36)
r=0.16
p=0.35
ImmuneScore
ART1 Expression

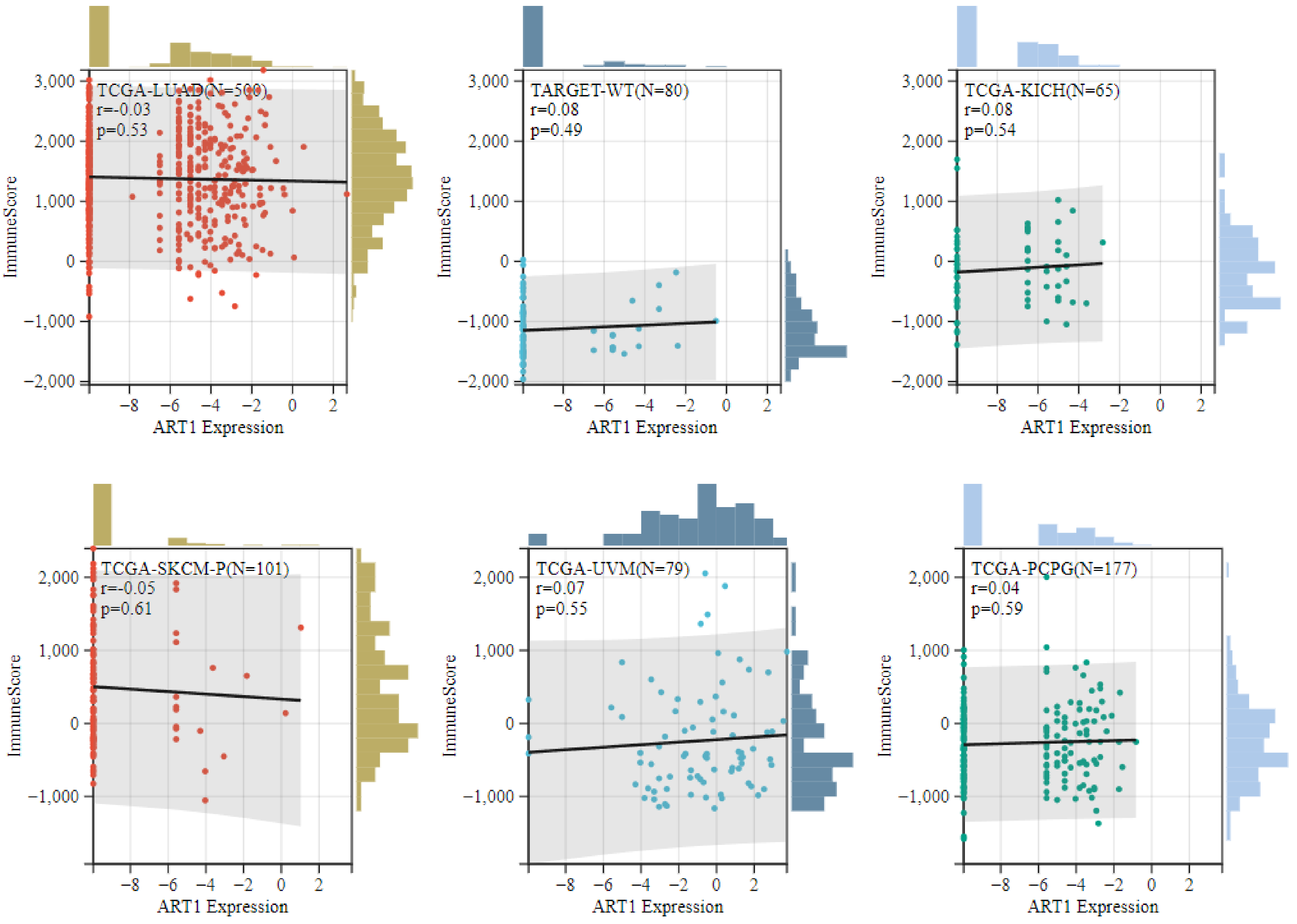

TCGA-LUAD(N=500)
r=-0.03
p=0.53
TARGET-WT(N=80)
r=0.08
p=0.49
TCGA-KICH(N=65)
r=0.08
p=0.54
TCGA-SKCM-P(N=101)
r=-0.05
p=0.61
TCGA-UVM(N=79)
r=0.07
p=0.55
TCGA-PCPG(N=177)
r=0.04
p=0.59
ImmuneScore
ART1 Expression

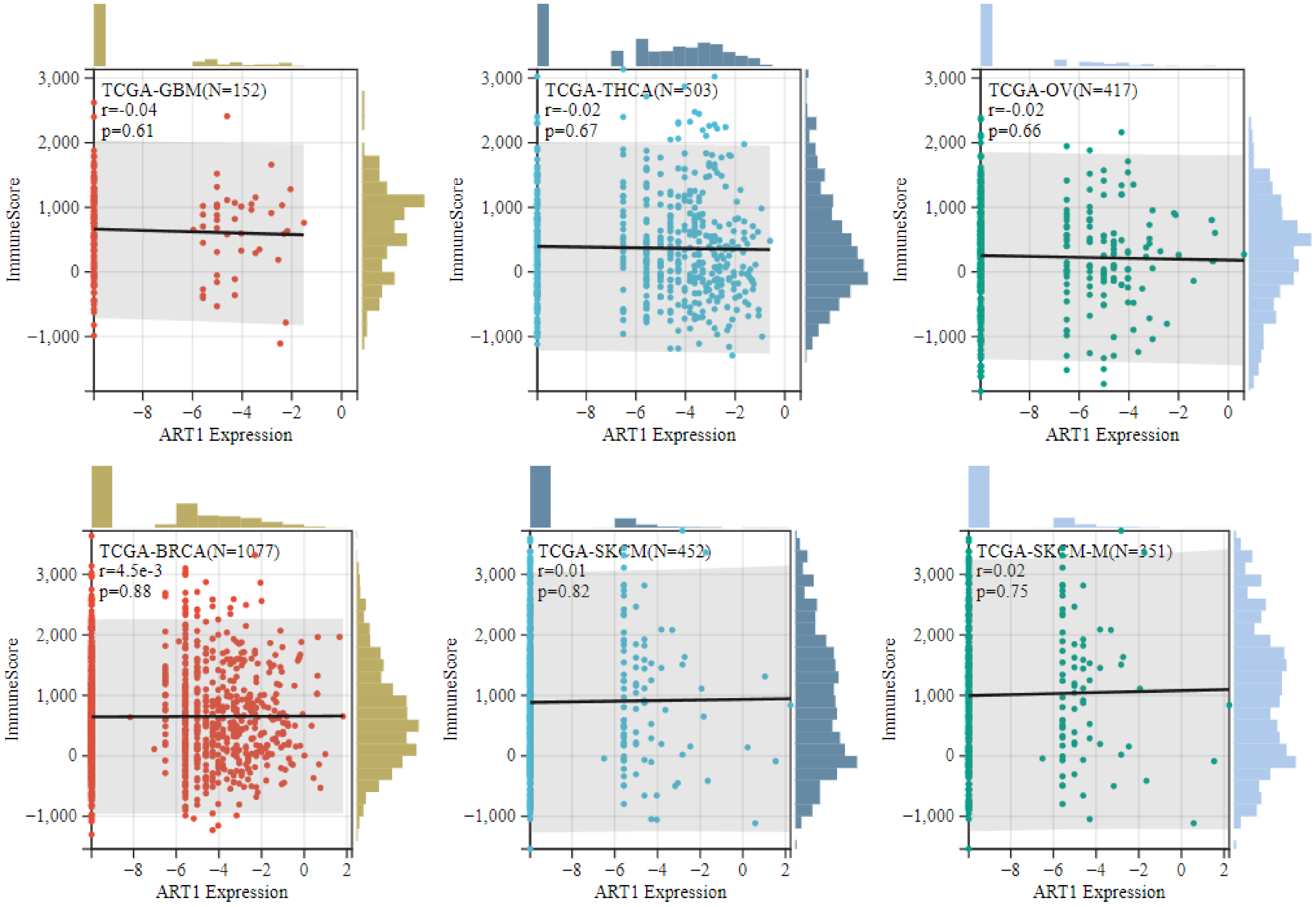

TCGA-GBM(N=152)
r=-0.04
p=0.61
TCGA-THCA(N=503)
r=-0.02
p=0.67
TCGA-OV(N=417)
r=-0.02
p=0.66
TCGA-BRCA(N=1077)
r=4.5e-3
p=0.88
TCGA-SKCM(N=452)
r=0.01
p=0.82
TCGA-SKCM-M(N=351)
r=0.02
p=0.75
ImmuneScore
ART1 Expression

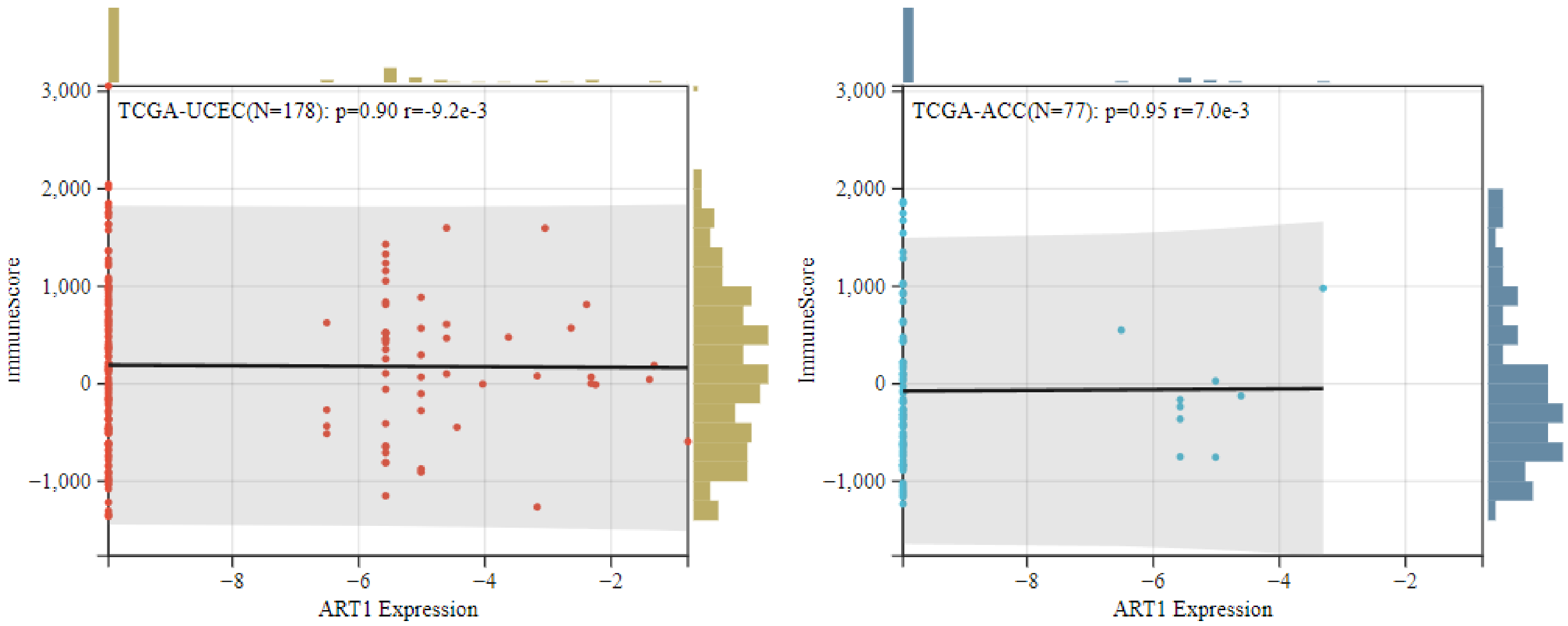

TCGA-UCEC(N=178): p=0.90 r=-9.2e-3
3,000
2,000
1,000
0
−1,000
ImmuneScore
−8
−6
−4
−2
ART1 Expression
TCGA-ACC(N=77): p=0.95 r=7.0e-3
3,000
2,000
1,000
0
−1,000
ImmuneScore
−8
−6
−4
−2
ART1 Expression

C Stromal Score of ART1 in pan cancers

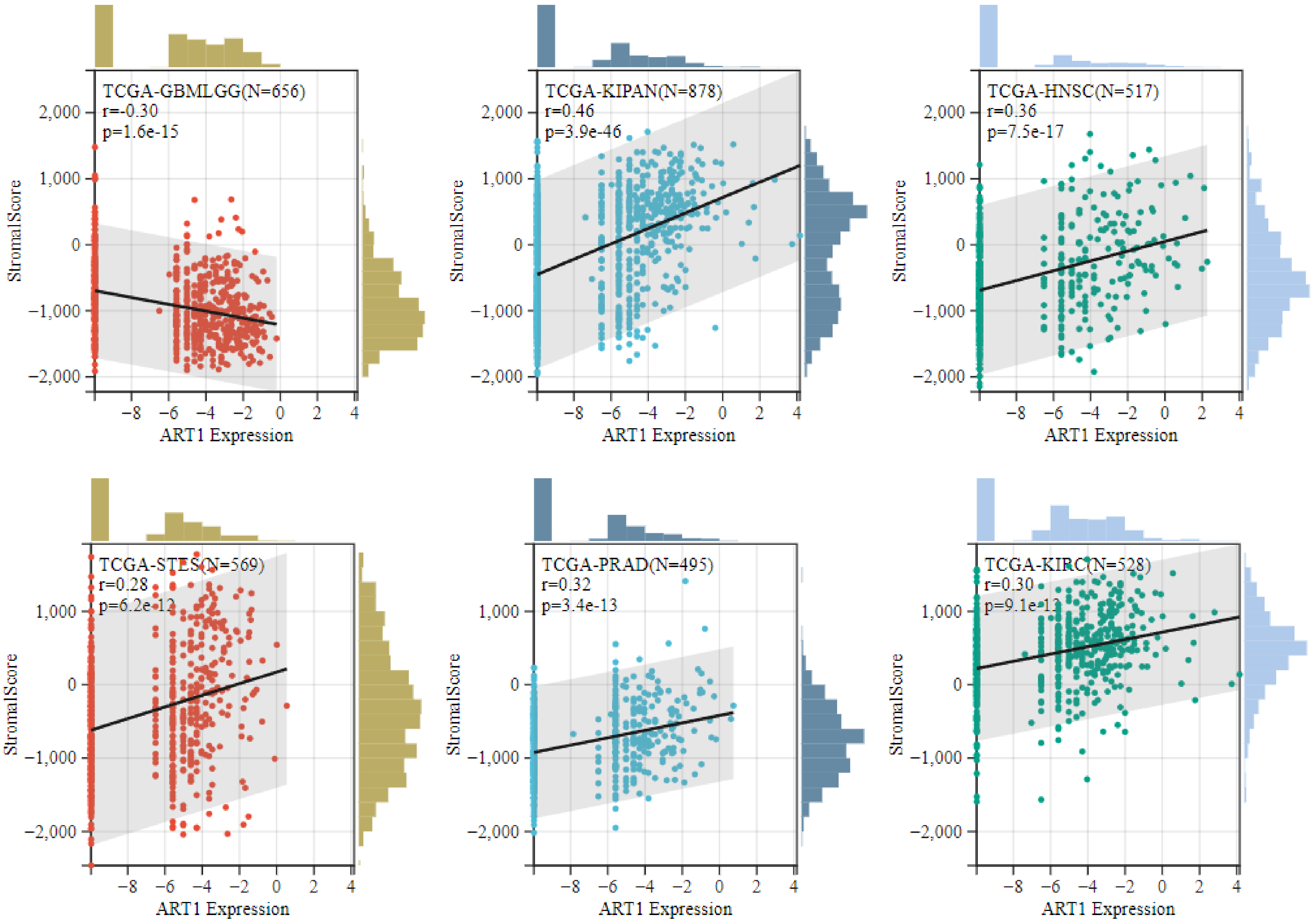

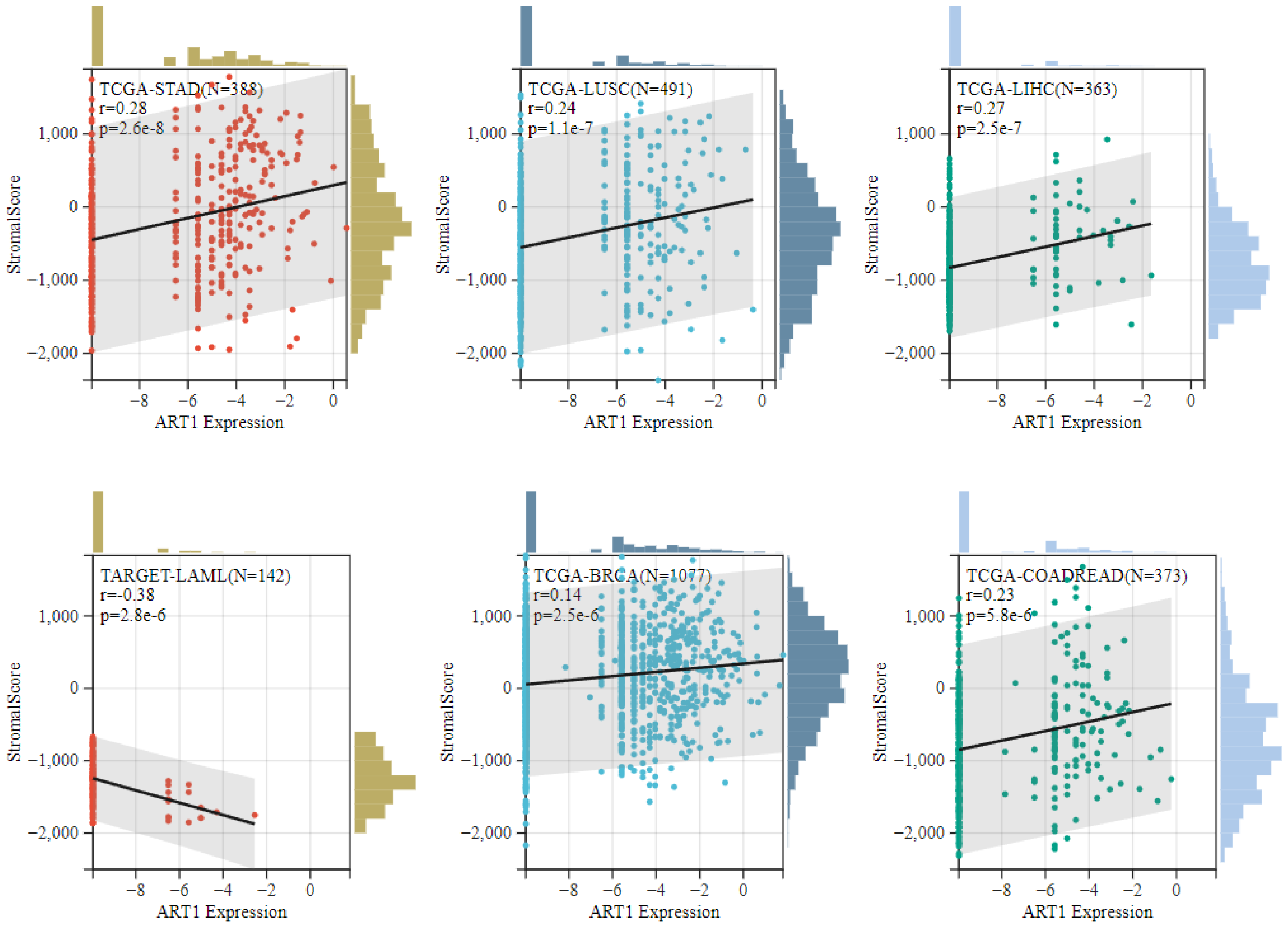
TCGA-STAD(N=388)
r=0.28
p=2.6e-8
TCGA-LUSC(N=491)
r=0.24
p=1.1e-7
TCGA-LIHC(N=363)
r=0.27
p=2.5e-7
TARGET-LAML(N=142)
r=-0.38
p=2.8e-6
TCGA-BRCA(N=1077)
r=0.14
p=2.5e-6
TCGA-COADREAD(N=373)
r=0.23
p=5.8e-6
StromalScore
ART1 Expression

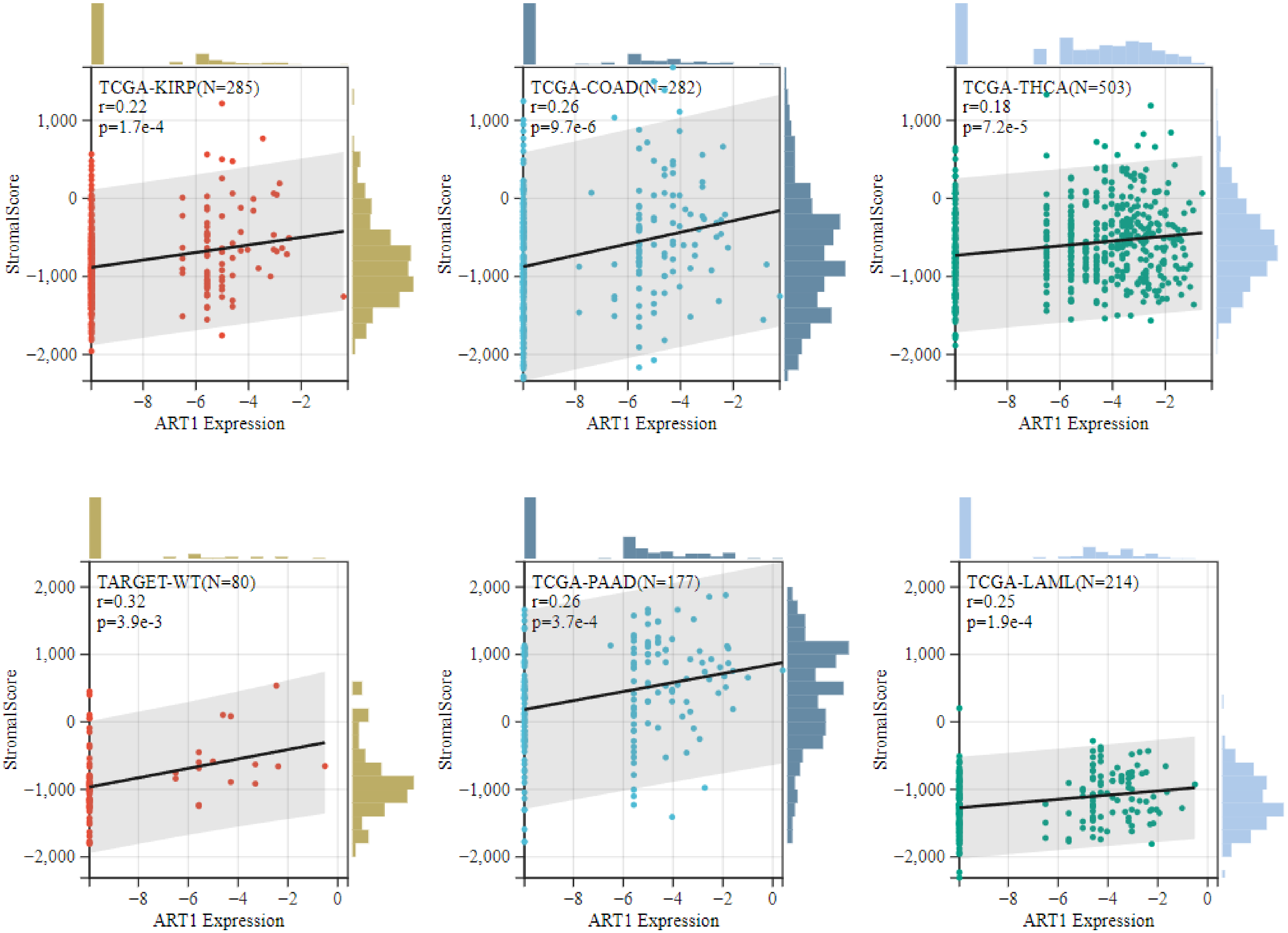
TCGA-KIRP(N=285)
r=0.22
p=1.7e-4
TCGA-COAD(N=282)
r=0.26
p=9.7e-6
TCGA-THCA(N=503)
r=0.18
p=7.2e-5
TARGET-WT(N=80)
r=0.32
p=3.9e-3
TCGA-PAAD(N=177)
r=0.26
p=3.7e-4
TCGA-LAML(N=214)
r=0.25
p=1.9e-4
StromalScore
ART1 Expression

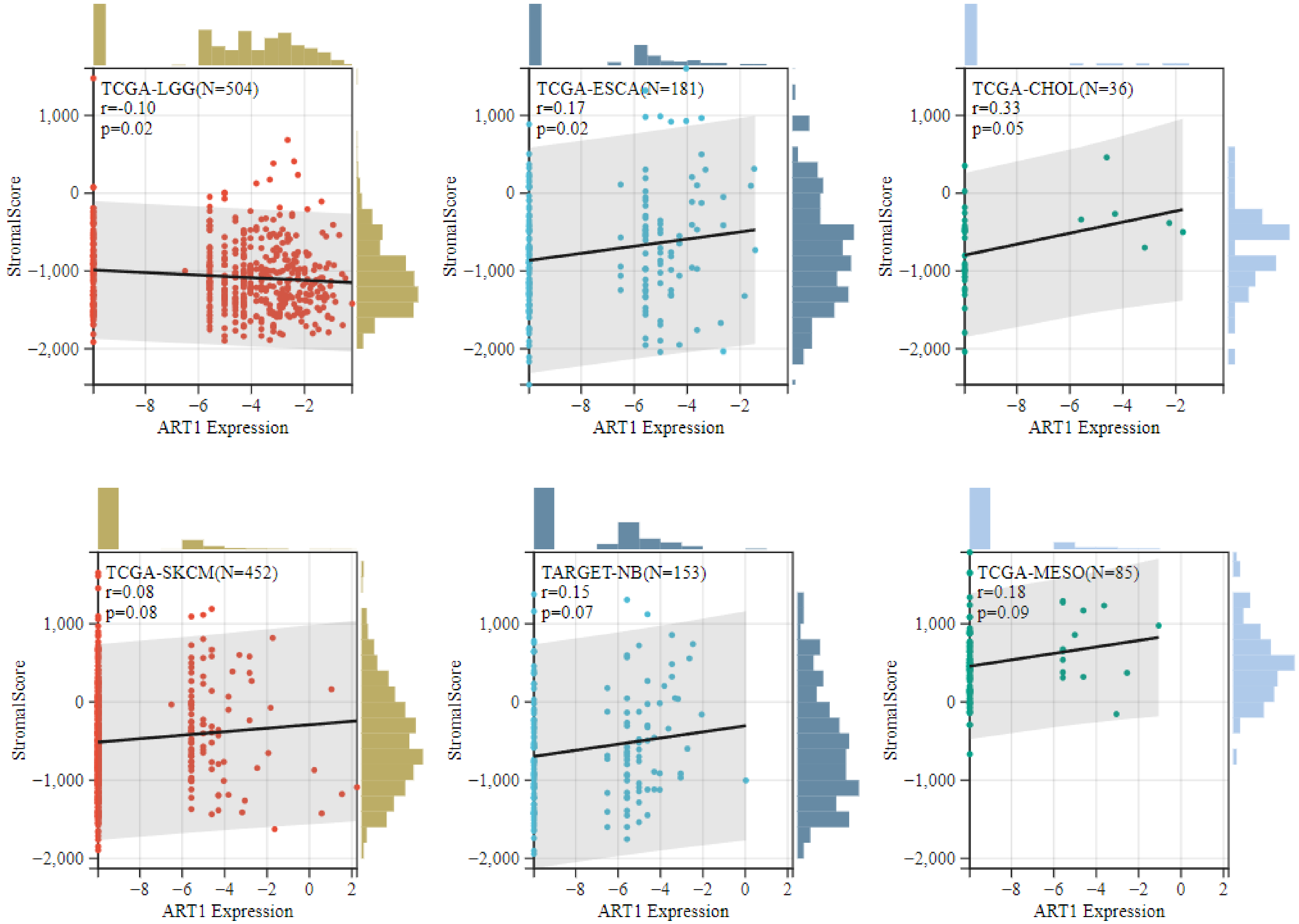

TCGA-LGG(N=504)
r=-0.10
p=0.02
TCGA-ESCA(N=181)
r=0.17
p=0.02
TCGA-CHOL(N=36)
r=0.33
p=0.05
TCGA-SKCM(N=452)
r=0.08
p=0.08
TARGET-NB(N=153)
r=0.15
p=0.07
TCGA-MESO(N=85)
r=0.18
p=0.09
StromalScore
ART1 Expression

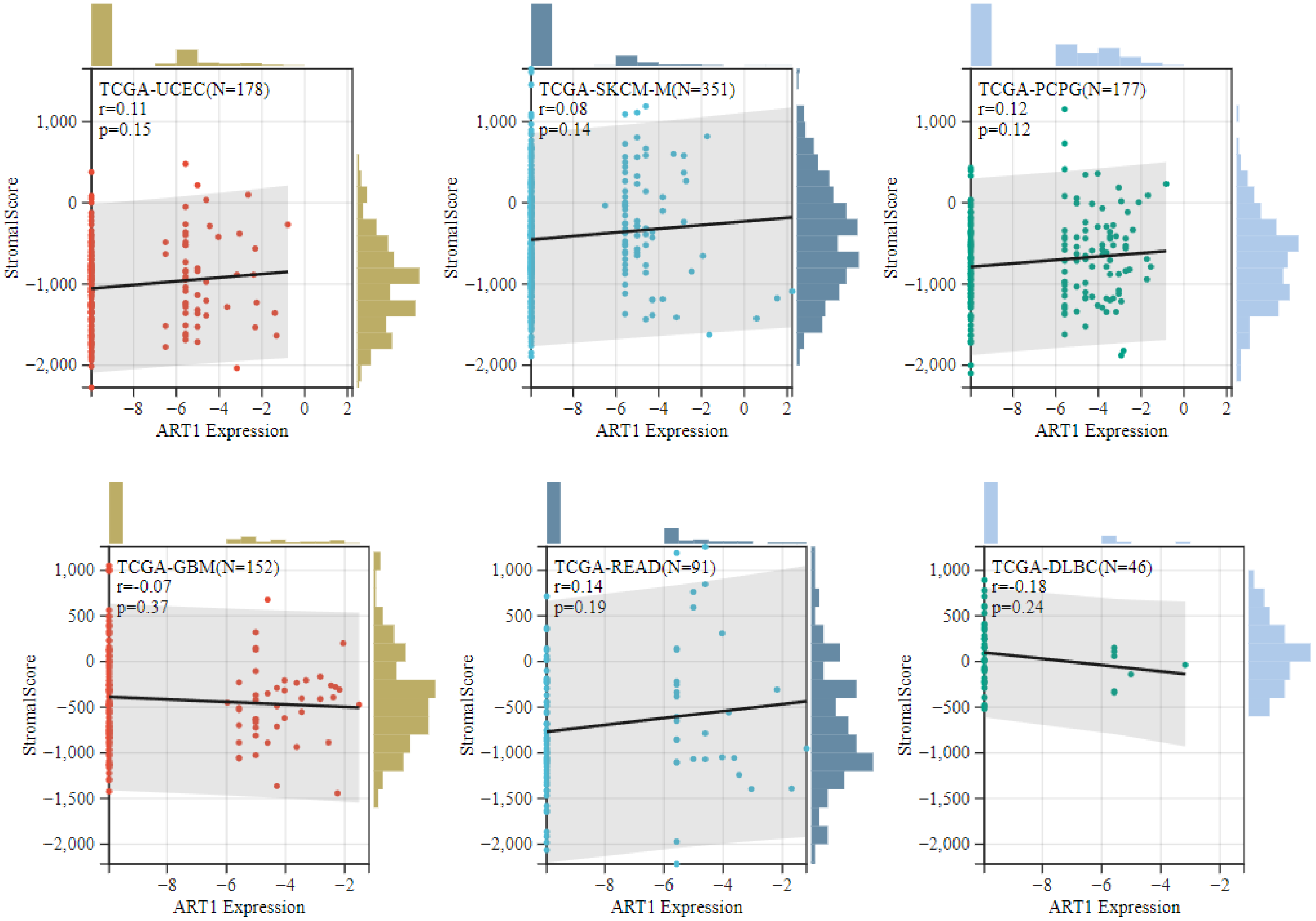
TCGA-UCEC(N=178)
r=0.11
p=0.15
TCGA-SKCM-M(N=351)
r=0.08
p=0.14
TCGA-PCPG(N=177)
r=0.12
p=0.12
TCGA-GBM(N=152)
r=-0.07
p=0.37
TCGA-READ(N=91)
r=0.14
p=0.19
TCGA-DLBC(N=46)
r=-0.18
p=0.24
StromalScore
ART1 Expression

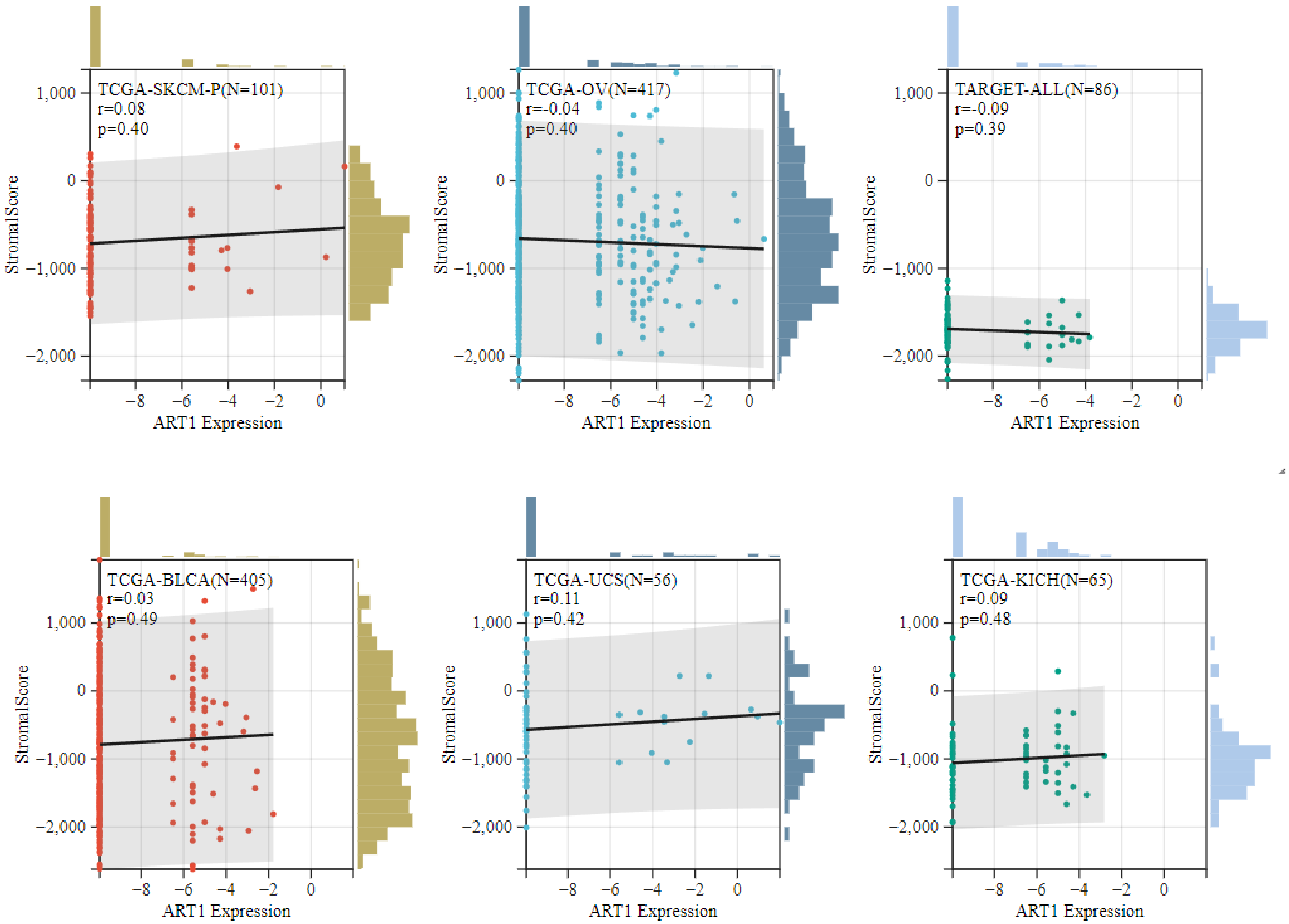

TCGA-SKCM-P(N=101)
r=0.08
p=0.40
StromalScore
ART1 Expression
TCGA-OV(N=417)
r=-0.04
p=0.40
StromalScore
ART1 Expression
TARGET-ALL(N=86)
r=-0.09
p=0.39
StromalScore
ART1 Expression
TCGA-BLCA(N=405)
r=0.03
p=0.49
StromalScore
ART1 Expression
TCGA-UCS(N=56)
r=0.11
p=0.42
StromalScore
ART1 Expression
TCGA-KICH(N=65)
r=0.09
p=0.48
StromalScore
ART1 Expression

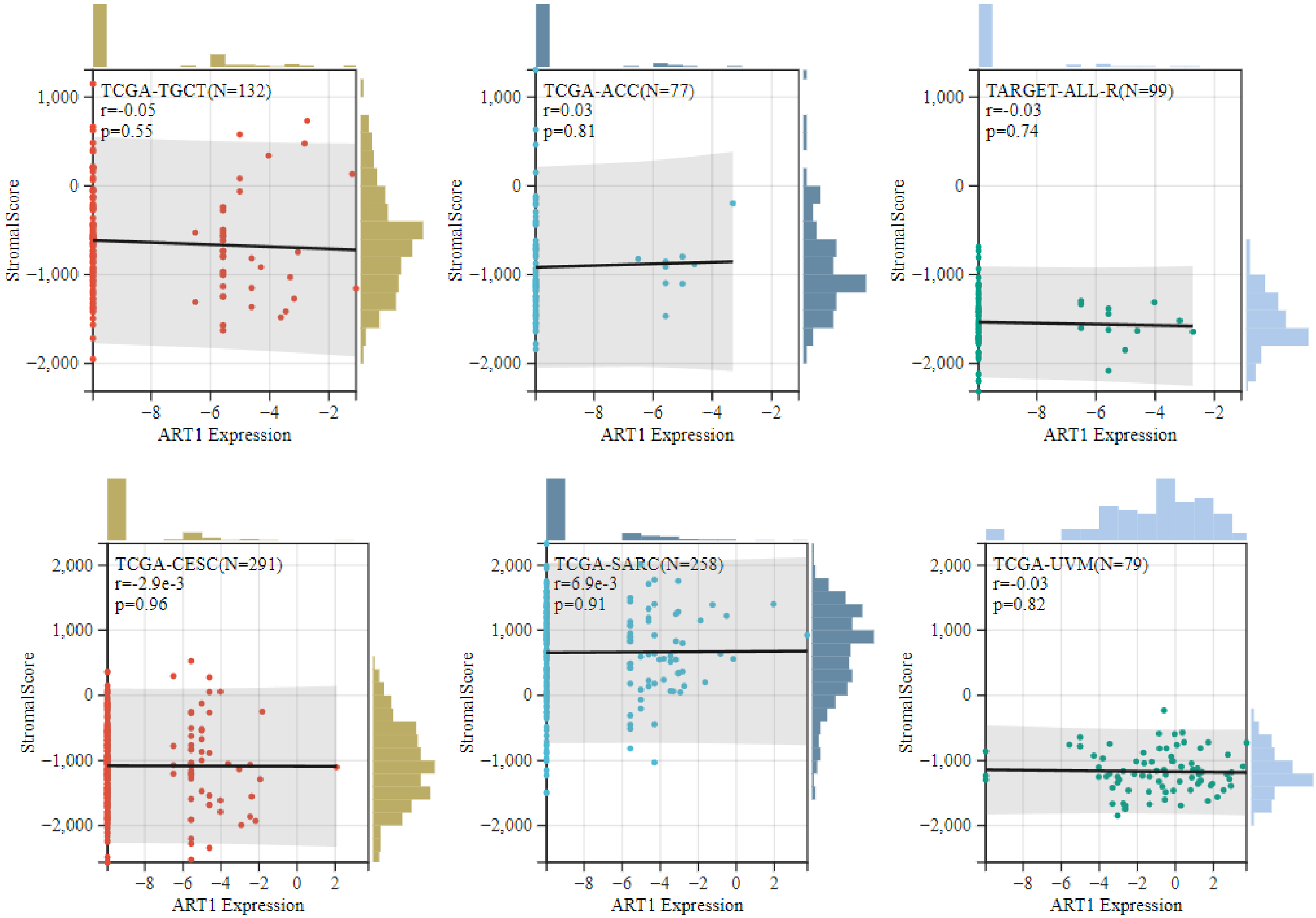
TCGA-TGCT(N=132)
r=-0.05
p=0.55
TCGA-ACC(N=77)
r=0.03
p=0.81
TARGET-ALL-R(N=99)
r=-0.03
p=0.74
TCGA-CESC(N=291)
r=-2.9e-3
p=0.96
TCGA-SARC(N=258)
r=6.9e-3
p=0.91
TCGA-UVM(N=79)
r=-0.03
p=0.82
StromalScore
ART1 Expression

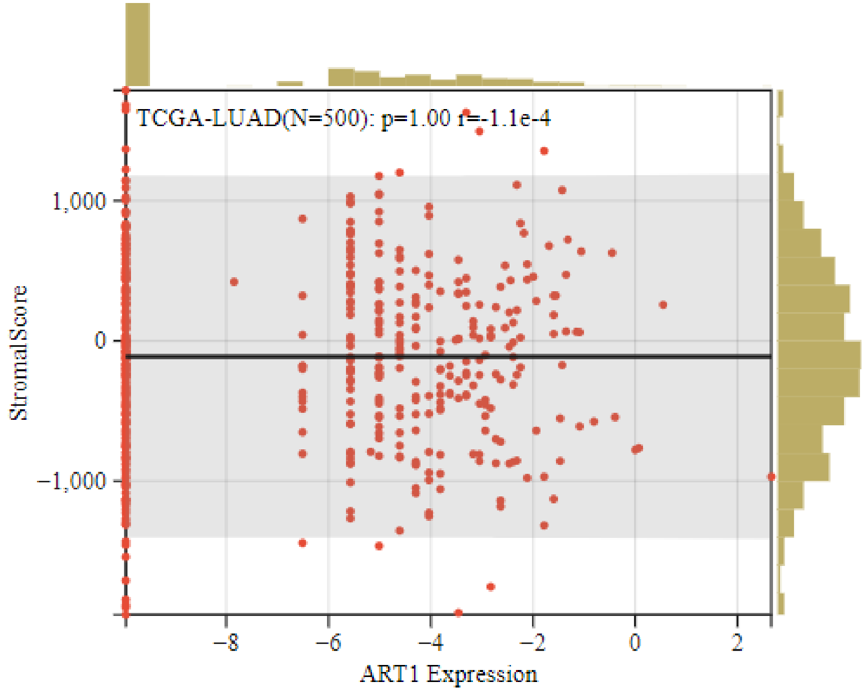

TCGA-LUAD(N=500): p=1.00 r=-1.1e-4
StromalScore
1,000
0
−1,000
−8
−6
−4
−2
0
2
ART1 Expression

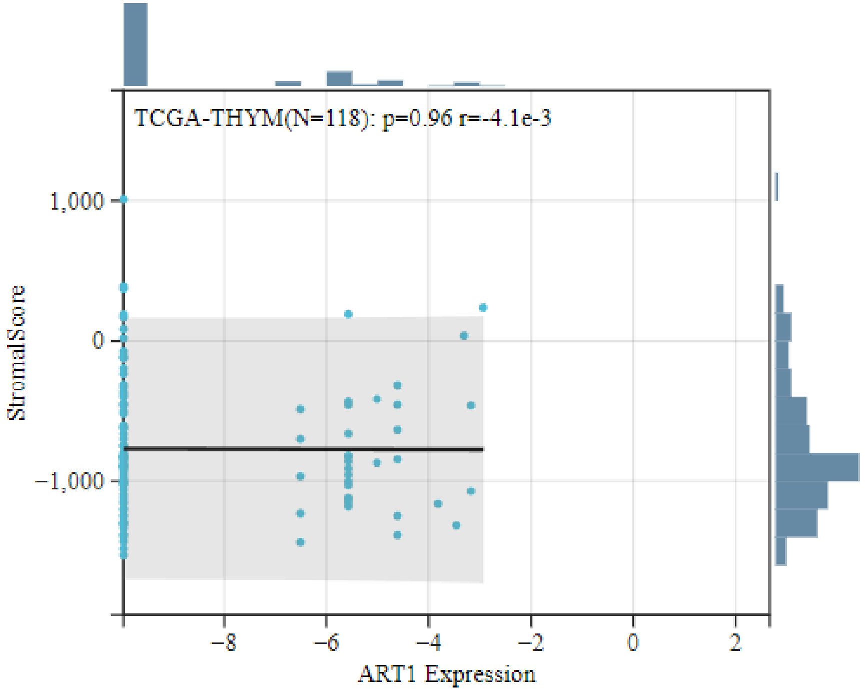

TCGA-THYM(N=118): p=0.96 r=-4.1e-3
StromalScore
1,000
0
−1,000
−8
−6
−4
−2
0
2
ART1 Expression
