# Supplementary figures and images for: Pan-Cancer Analysis of ART1 and its Potential Value in Gastric Cancer
Source: J Cancer. 2024 May 13;15(12):3684–707. doi: 10.7150/jca.96033 (PMC11190775; doi:10.7150/jca.96033)

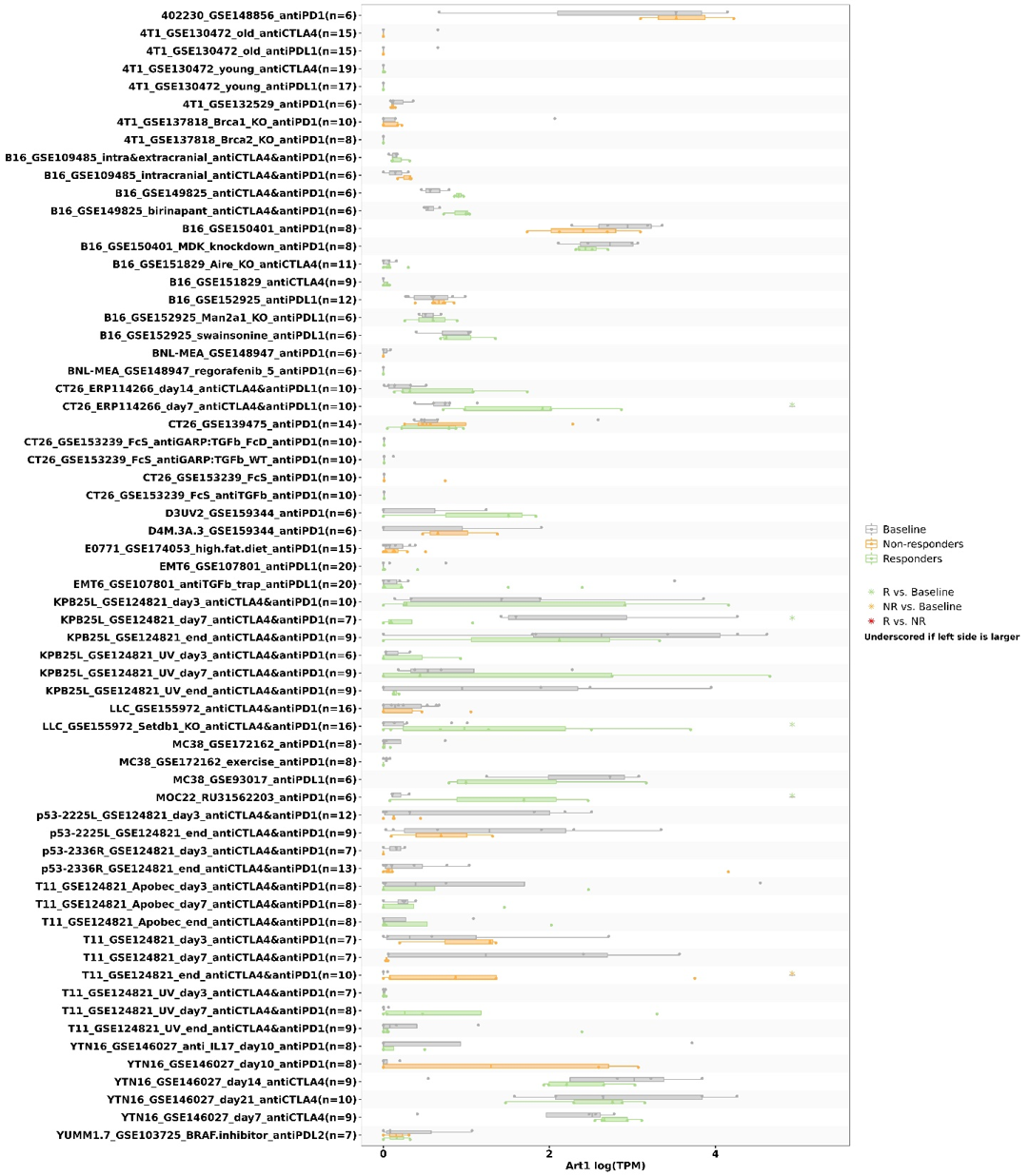

Supplement: Supplementary file 1 — Supplementary figures and tables. [file jcav15p3684s1.zip › Supplementary files/Supplementary Figure 2 Immunotherapy response of ART1 in immunotherapy cohort.tif]
